# Development of Geometry-Controlled All-Orthogonal BODIPY Trimers for Photodynamic Therapy and Phototheragnosis

**DOI:** 10.1021/acs.orglett.2c01169

**Published:** 2022-05-16

**Authors:** Alejandro Prieto-Castañeda, Fernando García-Garrido, Carolina Díaz-Norambuena, Blanca Escriche-Navarro, Alba García-Fernández, Jorge Bañuelos, Esther Rebollar, Inmaculada García-Moreno, Ramón Martínez-Máñez, Santiago de la Moya, Antonia R. Agarrabeitia, María J. Ortiz

**Affiliations:** † Departamento de Química Orgánica, Facultad de Ciencias Químicas, Universidad Complutense de Madrid, Ciudad Universitaria s/n, 28040 Madrid, Spain; ‡ Departamento de Química-Física, Universidad del País Vasco-EHU, Apartado 644, 48080 Bilbao, Spain; § Unidad Mixta UPV-CIPF de Investigación en Mecanismos de Enfermedades y Nanomedicina, Universidad Politécnica de Valencia, Centro de Investigación Príncipe Felipe, Carrer d’Eduardo Primo Yúfera 3, 46012 Valencia, Spain; ∥ Unidad Mixta de Investigación en Nanomedicina y Sensores, IIS La Fe, Universitat Politècnica de Valencia, Avda. de Fernando Abril Martorell 106, 46026 Valencia, Spain; ⊥ Instituto Interuniversitario de Investigación de Reconocimiento Molecular y Desarrollo Tecnológico (IDM), Universitat Politécnica de Valencia, Universitat de Valencia, Camino de Vera s/n, 46022 Valencia, Spain; # CIBER de Bioingeniería, Biomateriales y Nanomedicina (CIBER-BBN), Melchor Fernández Almagro 3, 28029 Madrid, Spain; ∇ Departamento de Sistemas de Baja Dimensionalidad, Superficies y Materia Condensada, Instituto de Química-Física “Rocasolano”, CSIC, Serrano 119, 28006 Madrid, Spain; @ Sección Departamental de Química Orgánica, Facultad de Óptica y Optometría, Universidad Complutense de Madrid, Arcos de Jalón 118, 28037 Madrid, Spain

## Abstract

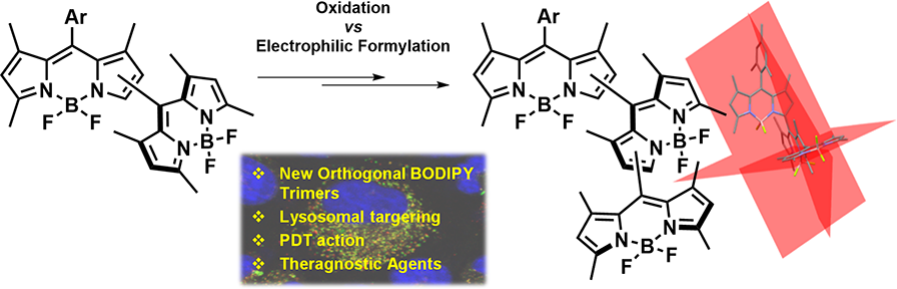

We have established an easy synthetic protocol for selectively developing all-orthogonal BODIPY trimers with unprecedented geometries on the basis of selecting methyl oxidation versus electrophilic formylation of key dimeric precursors. Photophysical characterization together with biological assays unraveled the most suitable BODIPY–BODIPY geometrical arrangements within the trimer, forcing them to serve as molecular platforms for the development of new, advanced heavy-atom-free photosensitizers for photodynamic therapy and phototheragnosis.

Photodynamic therapy (PDT) is a minimally invasive and clinically approved procedure based on the synergistic action of three elements: (i) a photoactivatable agent, the PDT photosensitizer (PS), (ii) light of a specific energy, and (iii) molecular oxygen. These three elements are not toxic by themselves, but their combination triggers a toxic effect on the basis of the generation of reactive oxygen species (ROS).^1,2^ Since its clinical approval in 1993, PDT has proven its efficacy in the treatment of multiple diseases related to high rates of cell proliferation and, especially, in the treatment of neoplastic diseases.^1^ However, the clinical application of PDT as a cancer first-line treatment remains limited and not fully exploited. For this reason, there are currently numerous investigations focused on improving the performance of PDT treatments and agents.^2^

The combination of PDT with diagnostic imaging leads to phototheragnosis, in which a single agent enables such dual phototriggered activity.^3^ It must be noted here that theragnosis constitutes a growing area of research, being considered one of the most promising precision medicine procedures, mainly in cancer.^4^ However, combining both capacities (PDT and imaging) in a biocompatible, simple, monochromophoric system is not easy, because the photonic properties required for each capacity are antagonistic (the higher the fluorescence efficiency, the lower the level of ROS photogeneration).^2,4^ Therefore, both key properties must be finely balanced to allow simultaneous fluorescence signaling for diagnosis and ROS-based cytotoxicity for PDT.^5^ In this scenario, the design of advanced phototheragnostic agents is one of the most challenging goals of modern biomaterials science.^5^

Among the most promising monochomophoric platforms for developing smarter PS for PDT and phototheragnosis, BODIPY dyes are at the forefront. These versatile fluorophores^6^ generally exhibit a negligible triplet state population due to their high quantum fluorescence yield; however, linking heavy atoms to the BODIPY structure is a facile approach for promoting the required intersystem crossing (ISC) populating the triplet manifold involved in ROS (singlet oxygen) formation.^7^ In this context, an appealing alternative to the use of heavy atoms is the design of orthogonal BODIPY dimers, because they are well-known efficient singlet oxygen photogenerators.^8^ Indeed, several orthogonal dimers have been reported to be PDT agents, mainly involving the 2–8′ BODIPY–BODIPY linkage,^8^ and less often the 3–8′ one (Figure 1a).^9^ On the contrary, all-orthogonal BODIPY trimers are rather scarce,^10^ and their performance as PSs for PDT has still not been fully explored (Figure 1b).^10b,10c^

**Figure 1:**
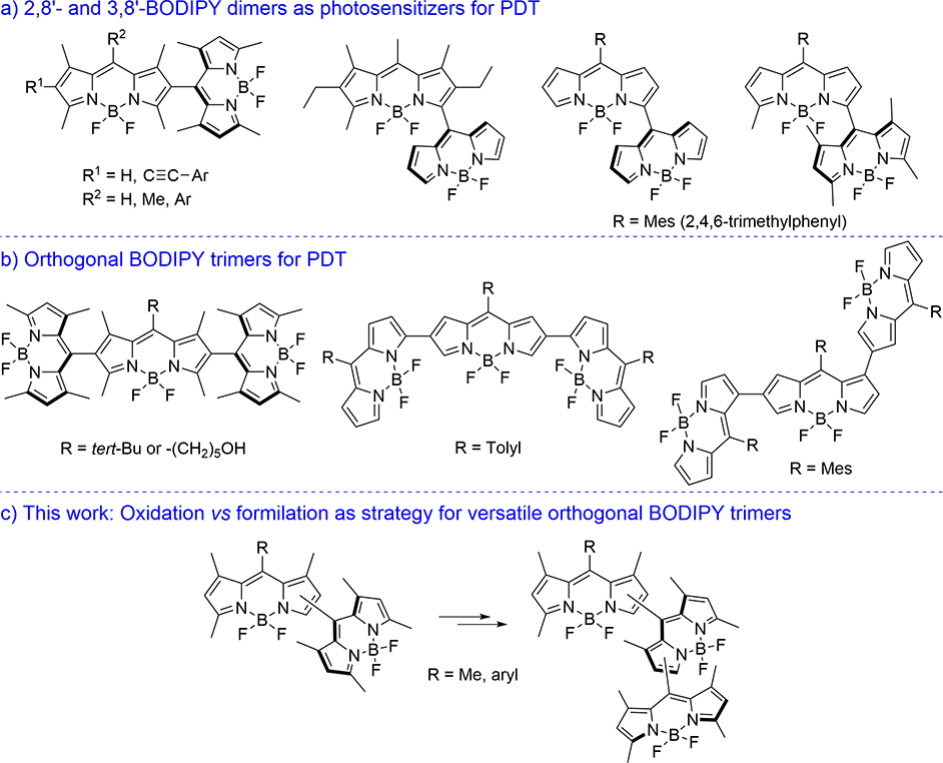
Existing (a) orthogonal BODIPY dimers and (b) all-orthogonal trimers and (c) new all-orthogonal BODIPY trimers developed in this work.

To address this gap and fully unlock the capabilities of all-orthogonal BODIPY trimers, we focused our attention on the possibility of obtaining trimers with different geometries (see Figure 1c) and studying the influence of these geometries (different BODIPY–BODIPY arrangements in the trimer) on the generation of singlet oxygen and fluorescent emission, to determine the privileged new molecular platforms for the development of advanced PDT and phototheragnostic agents. In this context, we hypothesized that a straightforward procedure for accessing all-orthogonal BODIPY trimers could be via regioselective formylation of dimeric precursors. It must be noted here that 2-formylBODIPYs can be straightforwardly and regioselectively obtained by Vilsmeier–Haack reaction,^11^ and recently, we have described an alternative method that allows easy access to 3-formylBODIPYs by oxidation of 3-methylBODIPYs using pyridinium chlorochromate (PCC).^9b^

Taking into account both possibilities (2-formylBODIPYs by electrophilic formylation vs 3-formylBODIPYs by methyl oxidation), we report here a comparative study of the application of both strategies to the preparation of formylBODIPY-based dimers involving different BODIPY–BODIPY linkages (2–8′ and 3–8′), as key synthetic precursors of unprecedented all-orthogonal BODIPY trimers, because they could serve as advantageous platforms for the development of advanced heavy-atom-free PDT and phototheragnostic agents.

Thus, we first studied the PCC-promoted oxidation of methyl groups in the 2–8′ dimers **1a**,^12^**1b** and **1c**, and **1d**,^13^ bearing an electron-donating *meso*-methyl (**1a**) or a *meso-*phenyl group of different electron richness in one of their BODIPY subunits (mesityl in **1b**, 4-methoxyphenyl in **1c**, or 4-nitrophenyl in **1d**). In all cases, 3-formylBODIPY-based dimers (see **2a–d** in Scheme 1A) were obtained in 54–64% yield. Interestingly, methyl oxidation exclusively took place at position 3 (3-methyl group) of the BODIPY subunit bearing a BODIPY rest at its *meso* position, regardless of the *meso* substitution of the other BODIPY subunit. These results constitute the first examples of regioselective mono-oxidation of 3-methylBODIPY-based dimers by PCC, expanding the interest in this reaction in the BODIPY chemistry field.^9b^

**Scheme 1:**
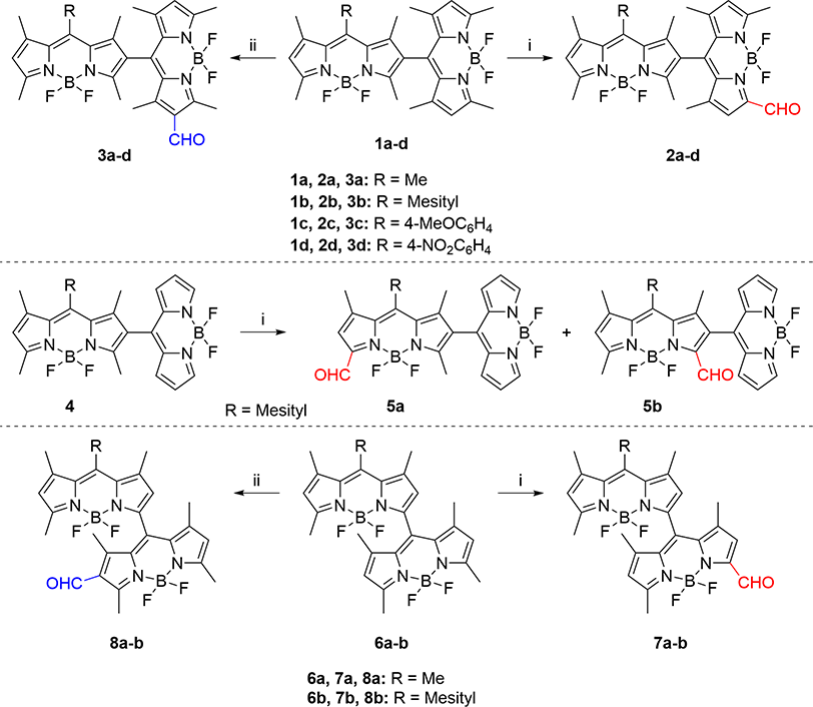
Synthesis of Mono-formylated Orthogonal BODIPY Dimers by (i) PCC Oxidation or (ii) Electrophilic Formylation with POCl_3_/DMF

On the contrary, the electrophilic formylation of the same dimers (**1a**, **1b** and **1c**, and **1d**) with POCl_3_/DMF was also studied. In this case, the reaction takes place at the BODIPY subunit bearing a BODIPY rest at its *meso* position, too, to generate 2-formylBODIPY-based dimers **3a–d** (Scheme 1A) in >80% yields. This regioselectivity agrees with the findings of Akkaya et al. in the up-to-now unique formylation of an orthogonal BODIPY dimer.^10a^

To further investigate the scope of the PCC oxidation of methylated BODIPY dimers, we selected the oxidation of dimer **4** (Scheme 1B), in which the reaction can take place only at the BODIPY subunit without a BODIPY rest at *meso*. In this specific case, methyl oxidation also occurs, but yielding a mixture of products (**5a** and **5b**) with low yield and regioselectivity (24% and 11%, respectively).

All of these results prompted us to extend the investigation of the application of both reactions, PCC methyl oxidation versus POCl_3_/DMF formylation, to two additional BODIPY dimers involving the uncommon 3–8′ linkage (**6a** and **6b** in Scheme 1C). To our satisfaction, the regioselectivity found in the PCC oxidation and POCl_3_/DMF formylation of the 2–8′-linked dimers **1a–d** is maintained in the 3–8′-linked dimers **6a** and **6b** to generate 3-formylBODIPY-based dimers **7a** and **7b**, respectively (by oxidation; 46% and 39% yields, respectively), and 2-formylBODIPY-based dimers **8a** and **8b**, respectively (by formylation; 64% and 78% yield, respectively) (see Scheme 1C). It must be remarked that **7a**, **7b**, **8a**, and **8b** are the first examples of formylated orthogonal BODIPY dimers involving the 3–8′ linkage. On the contrary, it should be noted that the position of the formyl group in all of the obtained formylated dimers was unequivocally established by one-dimensional NOESY experiments (e.g., see Figures S1–S5).

The obtained formylated BODIPY dimers should pave the way for all-orthogonal BODIPY trimers with different geometries upon standard BODIPY-core formation from formyl groups. To explore this possibility, we selected *meso-*mesitylated dimers **2b**, **3b**, **7b**, and **8b** (see Scheme 1), due to the known high photostability promoted by *meso*-mesitylation in BODIPY fluorophores.^14^ Satisfactorily, condensation of **2b**, **3b**, **7b**, and **8b** with 2,4-dimethylpyrrole in the presence of trifluoroacetic acid (TFA), followed by oxidation with 2,3-dichloro-5,6-dicyano-1,4-benzoquinone (DDQ), and final complexation with BF_3_·OEt_2_/triethylamine (standard BODIPY-core formation) gave rise to trimers **9–12**, respectively, in low to moderate yields (see Scheme 2).

**Scheme 2:**
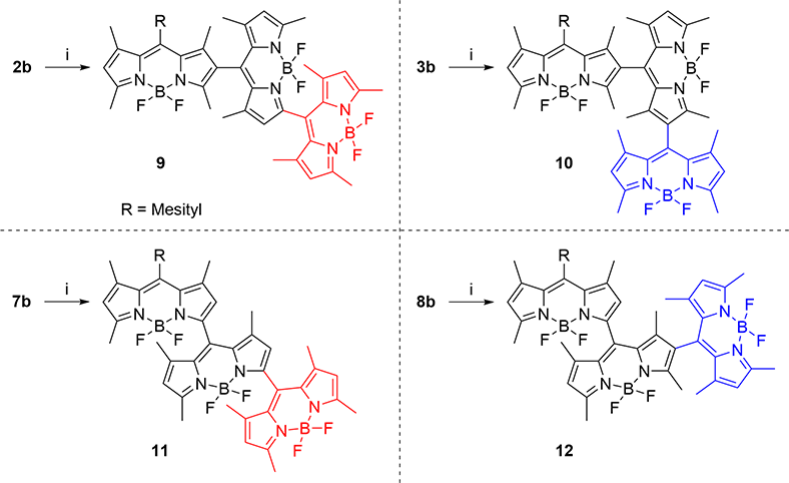
Synthesis of All-Orthogonal BODIPY Trimers **9–12** with Different Geometries Reaction conditions: (i) (a) 2,4-dimethylpyrrole, TFA, CH_2_Cl_2_; (b) DDQ; (c) BF_3_·Et_2_O/Et_3_N.

The obtained new, all-orthogonal BODIPY trimers (**9–12**) display their main absorption band in the same spectral region [centered at 505–510 nm (Figure S6)], resembling the absorption of each independent BODIPY subunit. Just a weak long-wavelength shoulder is recorded from trimer **12**, featuring both 2–8′ and 3–8′ junctions, which can be attributed to a small degree of excitonic coupling in such a geometry (Figure 2).^15^ Theoretically optimized geometries (CAM-B3LYP) reveal that the steric hindrance around both BODIPY–BODIPY linkage positions imposes an orthogonal disposition of the involved BODIPY subunits [torsion angles approaching 90° (Figure S7)], which hampers any resonant interaction between them. However, the molar absorption markedly depends on the linked chromophoric positions (see Table S1). Thus, trimer **10**, featuring solely 2–8′ linkages, displays huge molar absorption (reaching 230000 M^–1^ cm^–1^), in concordance with the expected additive contribution of the three involved BODIPY subunits. However, the mixing of the linkage positions (3–8′ and 2–8′ in **9** and **12**) implies a decrease in the absorption probability, reaching the lowest values for trimer **11** with just 3–8′ connectivities (down to 90000 M^–1^ cm^–1^). For the sake of simplicity, we theoretically simulated the absorption properties of the corresponding dimers **1b** and **6b** (Table S2), which show the photophysical trends observed in trimers (Tables S1 and S3). The photoexcitation of 2–8′-linked dimer **1b** implies the population of two excited states, each resulting from electron promotion in individual BODIPY subunits. Indeed, the molecular orbitals (MOs) involved in the electronic transitions to S_1_ and S_2_ are predominantly located in each BODIPY core, leading to additive allowed local excitations (LE) [HOMO–1 → LUMO and HOMO → LUMO (Figure S8)]. However, in 3–8′-linked dimer **6b**, the low-lying excited state has partial charge transfer (CT) character. Indeed, and in spite of the orthogonal arrangement, the occupied frontier MOs of both dimers are spread over the two BODIPY cores, whereas the unoccupied ones are exclusively located in one of the BODIPY cores (Figure S8). Therefore, the HOMO → LUMO transition in **6b** entails electronic transfer from one BODIPY subunit to another.^10c^ Note that such a weaker CT transition was fully forbidden in **1b**, but allowed in **6b**, and it is predicted to be at a position similar to that of the LE transitions of **1b** (Table S2), in agreement with the experimental findings.

**Figure 2:**
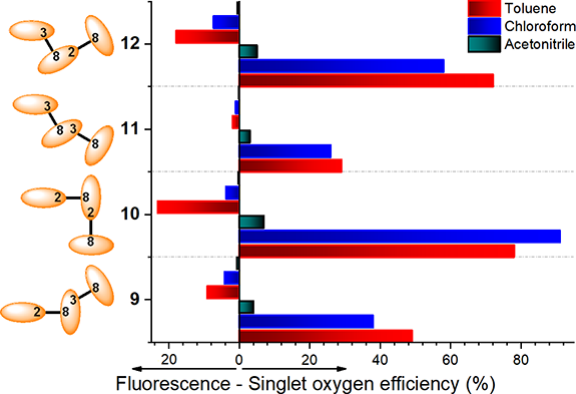
Fluorescence and singlet oxygen efficiency of all-orthogonal BODIPY trimers involving 2–8′ (**10**), 2–8′ and 3–8′ (**9** and **12**), and 3–8′ (**11**) BODIPY–BODIPY linkages in different solvents. Full photophysical data are listed in Table S1.

The fluorescence signatures also differ markedly depending on the geometry of the trimer. Thus, 2–8′-linked trimer **10** shows a single emission centered around 530–535 nm (Figure S6); its intensity decreases with solvent polarity [from 23% to almost 0% (Figure 2)]. As expected, the excited state dynamics are ruled by the orthogonal arrangement-induced intramolecular CT attributed to a symmetry-breaking mechanism (SBCT).^16^ In agreement with the absorption measurements, the presence of the 3–8′ linkage in the trimer implies a further stabilization of the CT, as reflected in lower fluorescence efficiencies [e.g., <2% in **11**, even in apolar media (Figure 2)]. Indeed, in trimers involving the 3–8′ junction (**9**, **11**, and **12**), the emission from the LE state is so weak that the ICT emission is detected at longer wavelengths in apolar and low-polarity solvents [shifted up to ∼610 nm in **12** and ∼675 nm in **11** (Figure S6)]. In more polar media, the charge separation (CS) is so stabilized that the ICT becomes a dark state and its emission vanishes, resulting in a single strongly quenched LE emission. The role of the geometry and the solvent is also reflected in the corresponding dimers (Table S3). Thus, 2–8′-linked dimer **1b** is more fluorescent than related trimer **10** based on it. The presence of two orthogonally linked BODIPY pairs in this trimer enhances the SBCT probability with the ensuing fluorescence quenching. However, the fluorescence response of 3–8′-linked dimer **6b** is weak and similar to that of its counterpart trimer **11**, supporting the stronger CS stabilization in this geometry arrangement.

CT states can mediate the triplet state population, promoting singlet oxygen generation by energy transfer (type II mechanism of ROS photogeneration),^10c,17^ as supported by the detection of the ^1^O_2_ phosphorescence at 1270 nm (see the experimental details in the Supporting Information). The most accepted mechanism, enabling the triplet state to be reached from the populated CT one, is spin–orbit charge transfer intersystem crossing (SOCT-ISC).^18^ All of the studied trimers show an efficient singlet oxygen photogeneration, which decays in polar media (Figure 2). This fact can be explained by the stabilization of a CS state, hindering the required charge recombination (CR) to reach the triplet manifold.^16^ Accordingly, these trimers show phosphorescence emission placed at 680–770 nm with a lifetime of ≤100 μs (Figure S9) measured from aerated solutions at room temperature. The effect of the trimer geometry on its behavior as a ROS photosensitizer can be rationalized in a similar way. Once again, the highest singlet oxygen efficiencies are achieved for 2–8′-linked trimer **10**, whereas the 3–8′ connection decreases the level of singlet oxygen photogeneration (see trimer **11** in Figure 2). The enhancement of the CS when it involves position 3 in the BODIPY–BODIPY linkage enables nonradiative relaxation channels from the ICT state, decreasing both fluorescence and ISC pathways. Further evidence is gathered upon inspection of the ROS generation capability of the corresponding dimers. These dimers show sizable efficiency (Table S3), though 2–8′-linked **1b** enables ^1^O_2_ generation even in polar media, while the rest of the dimers and the trimer show low efficiency (Figure 3). As mentioned above, the trimers are more prone to undergoing SBCT, the level of ROS generation being therefore high but more sensitive to the solvent polarity and the molecular geometry. Therefore, all of the developed trimers should be able to kill cells by PDT, but only the dual photonic behavior of trimer **10** and, to a lesser extent, **12** should allow phototheragnostic capability (Figure 2).

**Figure 3:**
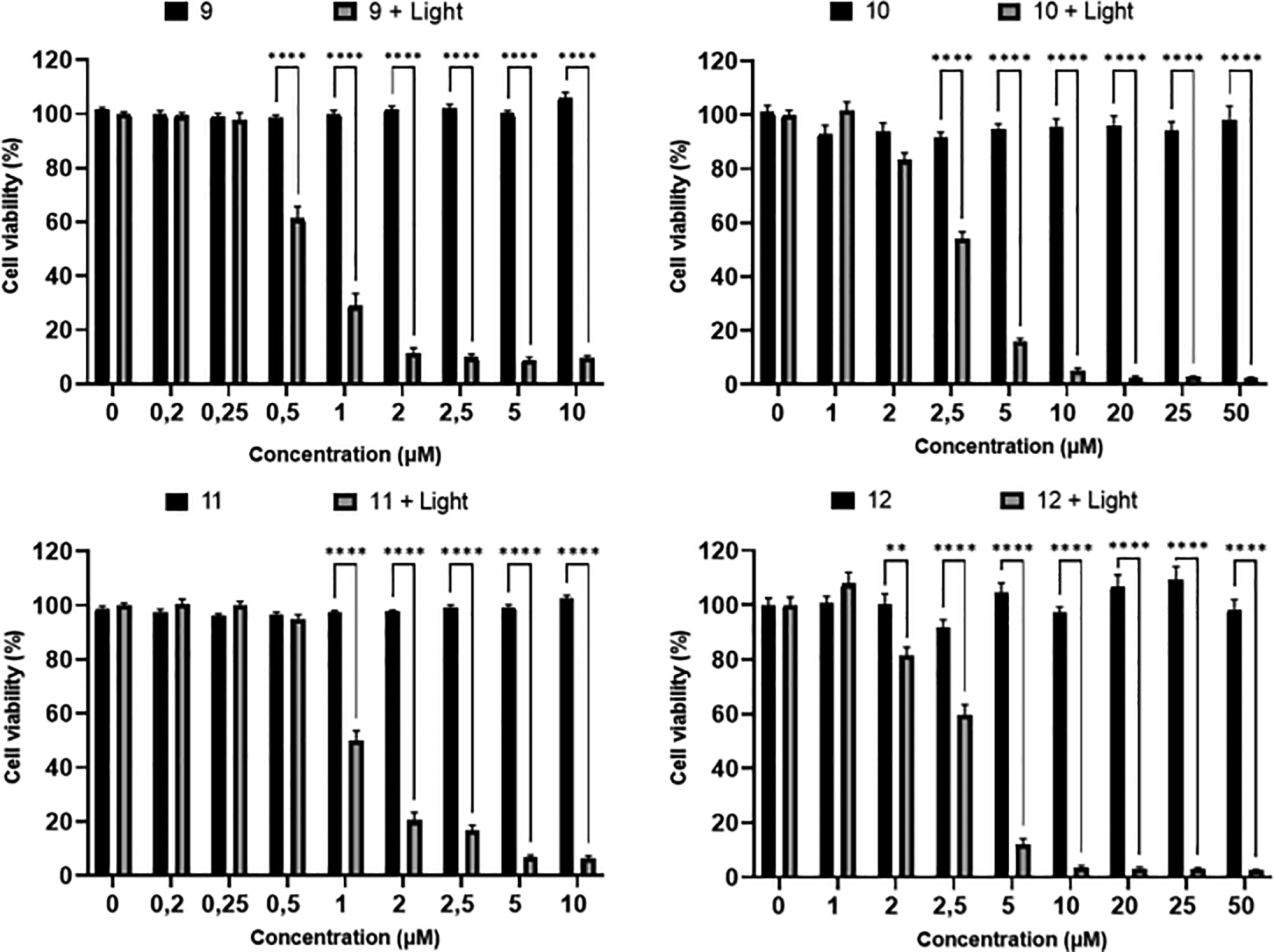
Cell viability of SK-Mel-103 cancer cells treated with trimers **9–12** (different concentrations) for 24 h in the absence (black) and presence (gray) of visible light (475 nm, 36 W) for 0.5 h. Values are expressed as means ± SEM of at least three independent experiments, and statistical significance was assessed by two-way ANOVA and Tukey’s post-test. **p < 0.010 and ****p < 0.0001 indicate statistically significant changes.

Accordingly, we evaluated the PDT activity of **9–12**. For this purpose, human melanoma cell line SK-Mel-103 and the cell viability WST-1 assay were selected. The cells were treated with increasing doses of the corresponding BODIPY trimer for 24 h and subsequently irradiated with LED light (475 nm, 36 W) for 0.5 h. As shown in Figure 3, all of the studied trimers display evident phototoxicity in a concentration-dependent manner [half-maximal inhibitory concentrations, IC_50_, between 0.69 and 2.80 μM (see Figure S10 and Table S4)]. By contrast, in the absence of light, no significant adverse effect on cells was detected. These results support trimers **9–12** being platforms for the development of PDT agents.

The significant fluorescent behavior of **10** and **12**, in conjunction with their PDT activity (Figures 2 and 3), prompted us to conduct further investigations to support their potential as phototheragnostic agents. Thus, we investigated the capability of these dyes to act as fluorescent intracellular makers. To our satisfaction, confocal laser scanning microscopy (CSLM) demonstrated that both dyes are internalized well into living SK-Mel-103 cells, preferably accumulating in the lysosomes without triggering cell death under the used microscopy conditions [e.g., Pearson’s correlation coefficient Rr of 0.70 ± 0.06 for **12** using LysoTracker Deep Red (see Figure 4 and Table S5)]. In contrast, when using the mitochondria and endoplasmic reticulum trackers, the two channels do not completely overlap with Pearson’s correlation coefficients decreasing (see Table S5 and Figures S11 and S12). All of these results demonstrate the capability of **10** and **12** to act as fluorescent intracellular probes, supporting their potential to serve as phototheragnostic agents. Moreover, apoptosis was confirmed as the main cell-death mechanism upon light irradiation (PDT treatment) when both dyes are individually used as PDT agents. Thus, flow cytometry shows an increase in the number of Annexin-V positive cells (a hallmark of apoptosis) after the selected PDT treatment (SK-Mel-103 cells; incubation with the dye for 24 h; 475 nm, 36 W, 0.5 h) with an increase in the concentration of the dye. For example, trimer **12** triggers cell death through apoptosis only after irradiation.^19^ The average percentage of early and late apoptotic cell population increased from 30.5% to 90.1% when the the dye concentration was increased from 2.84 μM [IC_50_ (see Figure 4)] to 5.00 μM. In contrast, the percentage of necrotic cells was not significant in either case, thus confirming apoptotic cell death (see Figure S13). A similar result was obtained when using **10** instead of **12** (see Figure S14). All of these studies and results support the potential of all-orthogonal BODIPY trimers **10** and **12** to serve as platforms for the development of advanced phototheragnostic agents.

**Figure 4:**
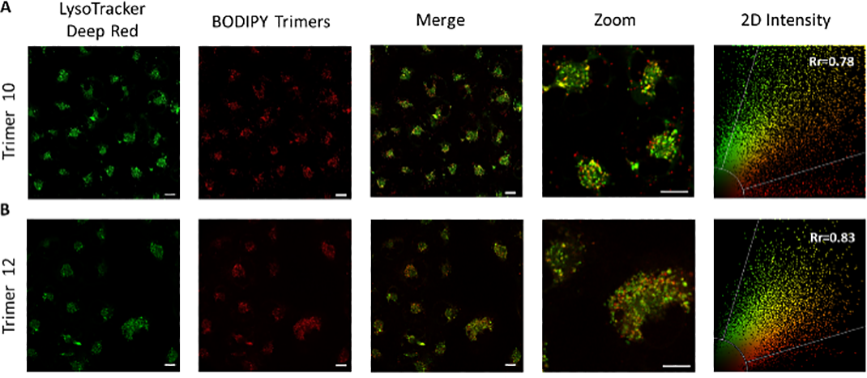
Confocal fluorescence images of subcellular co-localization studies of trimer **10** (2.5 μM) and trimer **12** (5.0 μM) in SK-Mel-103 cells stained with LysoTracker Deep Red. Areas of co-localization appear in yellow/orange in the Merge panels. Pearson’s co-localization coefficient (Rr), provided in the column of two-dimensional intensity, represents a correlation between pixel intensities between trimers and tracker channel in the close-up image. The scale bar is 10 μm.

In summary, a new synthetic strategy based of the regioselective formation of formylBODIPY-based dimers allows easy access to all-orthogonal BODIPY trimers with well-defined final geometries. Photophysical studies demonstrate that the involvement of 2–8′ BODIPY–BODIPY linkages in these trimers is advantageous for counterbalancing singlet oxygen generation and fluorescence toward phototheragnostic purposes. However, further CT state stabilization induced by the presence of 3–8′ linkages is detrimental for both key properties, sustaining the fundamental role of the fine control of the CT to develop smart phototheragnostic agents. Biological studies using SK-Mel-103 cells corroborate trimer photophysics, showing that all of the developed trimers display significant photocytoxicity, which is complemented by bioimaging capability (probing lysosomes) in the case of **10** and **12** involving 2–8′ linkages. These results support the utility of the developed synthetic strategy and the revealed privileged designs (based on 2–8′ BODIPY–BODIPY linkages) for the development of advanced heavy-atom-free PDT agents, including valuable phototheragnostic agents.

## Supporting Information Available

General methods, synthetic procedures, characterization data, including copies of ^1^H and ^13^C NMR spectra, and additional photophysical, computational, and biological results (PDF)
