# Supplementary material for: Development of Geometry-Controlled All-Orthogonal BODIPY Trimers for Photodynamic Therapy and Phototheragnosis
Source: Org Lett. 2022 May 16;24(20):3636–41. doi: 10.1021/acs.orglett.2c01169 (PMC9150176; doi:10.1021/acs.orglett.2c01169)
Supplement: Supplementary file 1 — ol2c01169_si_001.pdf [file ol2c01169_si_001.pdf]

Supporting Information (SI)

Development of Geometry-Controlled All-Orthogonal BODIPY Trimers for PDT and Photo-theragnosis

Alejandro Prieto-Castañeda,[†] Fernando García-Garrido,[†] Carolina Díaz-Norambuena,[‡] Blanca Escriche-Navarro,[§,¥,±] Alba García-Fernández,[§,±,µ] Jorge Bañuelos,[‡,*] Esther Rebollar,[¢] Inmaculada García-Moreno,[¢] Ramón Martínez-Máñez, [§,¥,±,µ,*] Santiago de la Moya,[†] Antonia R. Agarrabeitia,[†,‖] María J. Ortiz[†,*]

[†]*Departamento de Química Orgánica, Facultad de Ciencias Químicas, Universidad Complutense de Madrid, Ciudad Universitaria s/n, 28040 Madrid (Spain).*

[‡]*Departamento de Química-Física, Universidad del País Vasco-EHU, Apartado 644, 48080 Bilbao (Spain).*

[§]*Unidad Mixta UPV-CIPF de Investigación en Mecanismos de Enfermedades y Nanomedicina, Universidad Politécnica de Valencia, Centro de Investigación Príncipe Felipe, Carrer d'Eduardo Primo Yúfera 3, 46012 Valencia (Spain).*

[¥]*Unidad Mixta de Investigación en Nanomedicina y Sensores, IIS La Fe, Universitat Politècnica de Valencia, Avda. de Fernando Abril Martorell 106, 46026 Valencia (Spain).*

[±]*Instituto Interuniversitario de Investigación de Reconocimiento Molecular y Desarrollo Tecnológico (IDM), Universitat Politécnica de Valencia, Universitat de Valencia, Camino de Vera s/n, 46022 Valencia (Spain).*

[µ]*CIBER de Bioingeniería, Biomateriales y Nanomedicina,* (CIBER-BBN), *Melchor Fernández Almagro 3, 28029 Madrid (Spain).*

[¢]*Departamento de Sistemas de Baja Dimensionalidad, Superficies y Materia Condensada, Instituto de Química-Física "Rocasolano", CSIC, Serrano 119, 28006 Madrid (Spain).*

[‖]*Sección Departamental de Química Orgánica, Facultad de Óptica y Optometría, Universidad Complutense de Madrid, Arcos de Jalón 118, 28037 Madrid (Spain).*

Table of contents

**1. General methods**	S2
**2. Synthesis and characterization**	S5
**3. ^{1}H NMR and ^{13}C NMR spectra**	S16
**4. 1D-NOESY experiments**	S39
**5. Photophysical and computational results**	S42
**6. Biological studies**	S48
**7. References**	S51

1. General methods

Synthesis. All starting materials and reagents were commercial, unless otherwise indicated, and used without further purifications. Common solvents were dried and distilled by standard procedures. Flash chromatography was performed using silica gel (230-400 mesh). NMR spectra were recorded using $CDCl_3$ at 20 ºC. ^1H NMR and ^{13}C NMR chemical shifts (δ) were referenced to internal solvent $CDCl_3$ (δ = 7.260 and 77.04 ppm, respectively). DEPT 135 experiments were used to determine the type of carbon nucleus (C *vs* CH *vs* CH_2 *vs* CH_3). FTIR spectra were obtained from neat samples using the ATR technique. High resolution mass spectrometry (HRMS) were performed using the EI and MALDI-TOF techniques.

Spectroscopic measurements. The photophysical properties were registered in diluted solutions (around 2×10^{-6} M), prepared by adding the corresponding solvent (spectroscopic grade) to the residue from the adequate amount of a concentrated stock solution in acetone, after vacuum evaporation of this solvent. UV-Vis absorption and fluorescence spectra were recorded on a Varian model CARY 4E spectrophotometer and an Edinburgh Instruments spectrofluorometer (model FLSP 920), respectively. Fluorescence quantum yields (ϕ) were obtained from corrected spectra (detector sensibility to the wavelength) using PM546 (ϕ = 0.85 in ethanol) as reference.[1] The values were corrected by the refractive index of the solvent. Radiative decay curves were registered with the time correlated single-photon counting technique as implemented in the aforementioned spectrofluorometer. Fluorescence emission was monitored at the maximum emission wavelength after excitation by means of a Fianium pulsed laser (time resolution of picoseconds) with tuneable wavelength. The fluorescence lifetime (τ) was obtained after the deconvolution of the instrumental response signal from the recorded decay curves by means of an iterative method. The goodness of the exponential fit was controlled by statistical parameters (chi-square and the analysis of the residuals).

The photoinduced production of singlet oxygen ($^1O_2$) was determined by direct measurement of the luminescence at 1276 nm with a NIR detector integrated in the aforementioned spectrofluorometer (InGaAs detector, Hamamatsu G8605-23). The $^1O_2$ signal was registered in front configuration (front face), 40° and 50° to the excitation and emission beams, respectively and leaned 30° to the plane formed by the direction of incidence and registration in cells of 1 cm. The signal was filtered by a low cut-off of 850 nm. $^1O_2$-generation quantum yield (ϕ^Δ) was determined using the following equation:

$$\phi^\Delta = \phi^{\Delta,r}\cdot(\alpha^r/\alpha^{PS})\cdot(Se^{PS}/Se^r)$$

where $\phi^{A,r}$ is the quantum yield of $^1O_2$ generation for the used reference (2,6-diiodo-3,5-dimethyl-8-methylthioBODIPY, MeSBDP). Factor $\alpha = 1\text{-}10^{-Abs}$, corrects the different amount of photons absorbed by the sample (α^{PS}) and reference (α^{R}). Factor Se is the intensity of the $^1O_2$ phosphorescence signal of the sample (Se^{PS}) and the reference (Se^{r}) at 1276 nm. The singlet-oxygen quantum yield of the reference MeSBDP were 0.95, 0.91 and 0.95 in toluene, chloroform and acetonitrile respectively. $^1O_2$ quantum yields were averaged from at least five concentrations between 10^{-6} M and 10^{-5} M.

Delayed spectroscopy. Aerated solutions at room temperature of **9-12** contained in 1 cm optical-path rectangular quartz cells were transversally pumped with intense laser pulses (energy fluence of 5 mJ/cm^2) from the second (532 nm) harmonic of a Nd:YAG laser (LOTIS TII, LS-2147) at 10 Hz repetition rate. A solution volume of 3 cm^3 was used in order to avoid (or at least reduce) the risk of photo-bleaching the sample during the experiments. The time-gated emission upon laser photoexcitation, analyzed perpendicularly to the input radiation, was focused onto a spectrograph (Kymera 193i-A, Andor Technologies) coupled to an intensified CCD camera (iStar, Andor Technologies). The camera enabled gate widths ranging from nanoseconds up to seconds and its opening can be delayed in a controlled way with respect to the incoming pump laser pulse. Neither long-pass-filters nor band-pass filters were used to remove the excitation laser since we have verified that these filters, especially long pass filters, under drastic pump conditions, exhibited its own fluorescence and/or phosphorescence emission, which could lead to misunderstand the experimental results. Each spectrum is the average of at least 200 scans recorded with a gate time of 10, 50 and 100 μs. This experimental set-up allowed to carry out the projected measurements even under adverse conditions, but avoided to determine properly the efficiency of the delayed emission.

Quantum mechanical calculations. Ground state geometries were optimized with the B3LYP hybrid functional, within the Density Functional Theory (DFT), corrected with the Coulomb attenuating method (CAM) to account for long range interactions like charge transfer excitations, using the triple valence basis set with a polarization function (6-311g*). The geometries were considered as energy minimum when the corresponding frequency analysis did not give any negative value. The simulation of the absorption spectra as vertical Franck-Condon transitions was carried out using the Time Dependent (TD) method with the same functional (CAM-B3LYP) and basis set used for the energy minimization. All the calculations were performed in the Gaussian 16 implemented in the “arina” computational cluster of the UPV/EHU.

Cell culture conditions. The human melanoma cell line SK-Mel-103 was cultured in Dulbecco's Modified Eagle Medium (DMEM) supplemented with 10% fetal bovine serum (FBS) and maintained at 37 ^{o}C in an atmosphere of 20% O_2 and 5% CO_2. All-orthogonal BODIPYs trimers **9-12** were prepared in DMSO and kept at -20 ^{o}C prior to use in cell culture.

Cell Viability Assay. The cytotoxic effect of all-orthogonal BODIPYs trimers **9-12** was determined using the WST-1 Assay for Cell Proliferation and Viability (Roche). SK-Mel-103 cells were seeded in 96-well plates (5×10^3 cells/well) and incubated for 24 h at 37 ^{o}C under 5% CO_2. Then, the cells were treated with different concentrations of trimers ranging from 0.2 to 50 µM prepared in DMEM (containing 1% DMSO). After incubation for another 24 h, the cells were rinsed with PBS, infused with fresh medium, and illuminated with a 36 W LED device at a 10 cm distance (900 mW/cm^2) for 30 min (*i.e.*, 1620 J/cm^2 light dose). A light filter was used to allow the passage of only appropriate wavelengths (Newport, λ > 475 nm). After further incubation for 24 h, 7 µL of WST-1 were added to each well and the plates were incubated for 1 h at 37 ^{o}C. The absorbance of samples was measured using a spectrophotometer Wallac 1420 Victor2 Microplate Reader (PerkinElmer) at a wavelength of 450 nm. The dark toxicity of trimers was evaluated under the same conditions as above but omitted the illumination step. The IC_{50} value (concentration of compound which reduces 50% of cell viability compared to untreated controls) was estimated from a sigmoidal dose-response curve fit of the photocytotoxicity data from at least three independent analyses.

Subcellular localization studies. SK-Mel-103 cells (2.5×10^5 cell/well) were seeded on 24 mm ϕ glass coverslips in 6-well plates and incubated 24 h to adhere. Cells were then treated for another 24 h with 2.5 µM and 5.0 µM solution of trimers **10** and **12**, respectively. After were rinsed with PBS, the cells were stained with different organelle-specific trackers (Molecular Probes® by Life Technologies). For labeling of the mitochondria, cells were treated with MitoTracker Deep Red FM (250 nM) for 1 h, for labeling lysosomes with LysoTracker Deep Red (75 nM) for 1 h and for labeling the endoplasmic reticulum with ER-Tracker Blue-White DPX (0.5 µM) for 30 min. Subsequently, the cells were examined with a Leica TCS SP8 high-speed confocal microscope equipped with 405, 488, 522, and 638 nm lasers using 63X objective lens. The parameter of confocal laser scanning microscope (CLSM) was: Trimers **10** and **11** were excited at 488 nm and emission collected in the region 513-567 nm, MitoTracker® Deep Red FM and LysoTracker® Deep Red were excited at 638 nm and emission wavelength collected at 640-780 nm, and ER-Tracker Blue-White DPX was excited at 405 nm and emission collected at 410-480 nm. The images, Pearson's co-localization coefficient (Rr) and 2D

scatterplots of colocalization were acquired and analyzed with Leica Application Suite X software.

Apoptosis assay with Annexin V-APC/DAPI. SK-Mel-103 cells were cultured in 12-well plates at a cell density of 1×10^5 cells/well and allowed to settle for 24 hours. The medium was then changed to a fresh medium containing **10** and **12** trimers at a concentration of IC_{50} and 5 µM. After 24 h, the cells were irradiated (900 mW/cm^2) for 30 min (*i.e.*, 1620 J/cm^2 light dose). Following further incubation for 24 h, treated cells, both floating and adherent, were collected, centrifuged (300 x g, 5 min, 4 °C), and suspended in 0.2 mL of Annexin V binding buffer (1X) containing 5 µL of Annexin V conjugated with allophycocyanin (APC). After incubation for 15 min at 4 °C in the dark, 1 µL of DAPI (1 mg/mL) was added to each sample and it was determined whether the cells were viable (Annexin V-, DAPI-negative), early apoptotic (Annexin V-positive, DAPI-negative) late apoptotic/necrotic (Annexin V-, PI-positive) or necrotic (Annexin V-negative, DAPI-positive) by flow cytometry (CytoFlexS instrument, Beckman Coulter). Data analysis was performed with CytoExpert software.

Statistical analysis. All biological experiments were performed at least three times with representative results depicted in this report. Data are presented as means ± standard deviation of the mean (SEM) of at least three independent experiments with software GraphPad Prism 8.0. Statistical analysis was performed with one-way ANOVA or two-way ANOVA (analysis of variance), and Tukey's post-test as indicated in the figure legends. Statistical significance is indicated as * $p<0.05$, ** $p<0.01$, *** $p<0.001$ and **** $p<0.0001$.

2. Synthesis and characterization

Orthogonal BODIPY dimers **1a**[2] and **1d**[3] were synthesized by the corresponding described methods.

2.1. General procedure for the synthesis of BODIPY dimers and trimers. To a solution of formylBODIPY (1 equiv) in dry CH_2Cl_2 were added 2,4-dimethylpyrrole (2.1 equiv), or pyrrole (6 equiv), and trifluoroacetic acid (TFA) (two drops), and the reaction was stirred at rt for 30 min-6 h. After disappearance of the starting material, a solution of DDQ (1.1 equiv) in CH_2Cl_2 was added, and the mixture was stirred for 30 min. Et_3N (5 equiv) and $BF_3\cdot Et_2O$ (5 equiv) were then added, and the mixture was stirred for 3 h. Finally, the reaction was washed with HCl 10% solution and H_2O, and the extract was dried over anhydrous Na_2SO_4, filtered and evaporated to dryness. The obtained residue was submitted to purification by flash chromatography on silica gel.

2.2. General procedure for oxidation reactions of methylBODIPYs. To a degassed solution of 3-methylBODIPY (1 equiv) in EtOAc or 1,2-dichloroethane (DCE) was dropwisely added a solution of PCC (6 equiv) in EtOAc or DCE. The reaction mixture was stirred at rt or 60 ºC for 7-24 h. The reaction progress was monitored by TLC. Once the reaction was completed, the mixture was filtered over silica gel (EtOAc or DCE were used for elution and washing), and the obtained solution was submitted to solvent evaporation under vacuum. The obtained residue was submitted to purification by flash chromatography on silica gel.

2.3. General procedure for formylation reactions of BODIPYs. A mixture of $POCl_3$ (15 equiv) and DMF (30 equiv) was stirred in an ice bath for 5 min under argon. After being warmed to rt, it was stirred for additional 30 min, and then, BODIPY (1 equiv) in DCE was added. The reaction was raised to 60 ºC and stirred for 1 h. The reaction progress was monitored by TLC. Once the reaction was completed, the mixture was cooled to rt and slowly poured into saturated aqueous $NaHCO_3$ (100 mL) under ice-cold conditions, and stirring maintained for 1 h. Finally, the reaction mixture was diluted with CH_2Cl_2 and washed with H_2O. The organic layer was dried over anhydrous Na_2SO_4, filtered and evaporated to dryness. The obtained residue was submitted to purification by flash chromatography on silica gel.

2.4. Synthesis and characterization of compounds:

1b: According to general procedure 2.1., 2-formyl-8-mesityl-1,3,5,7-tetramethylBODIPY[4] (165 mg, 0.42 mmol), 2,4-dimethylpyrrole (0.09 mL, 0.88 mmol) and TFA (two drops) in CH_2Cl_2 (20 mL) were reacted for 30 min. Then, a solution of DDQ (140 mg, 0.46 mmol,) in CH_2Cl_2 (10 mL), Et_3N (0.29 mL, 2.10 mmol) and $BF_3·Et_2O$ (0.26 mL, 2.10 mmol) were added to the mixture. Flash chromatography using hexane/CH_2Cl_2 (60:40) afforded **1b** (197 mg, 76%) as an orange solid. ^1H NMR (300 MHz, $CDCl_3$) δ 6.95 (s, 2H, 2CH), 6.05 (s, 1H, CH), 5.98 (s, 2H, 2CH), 2.60 (s, 3H, CH_3), 2.53 (s, 6H, 2CH_3), 2.41 (s, 3H, CH_3), 2.32 (s, 3H, CH_3), 2.10 (s, 6H, 2CH_3), 1.69 (s, 6H, 2CH_3), 1.42 (s, 3H, CH_3), 1.22 (s, 3H, CH_3) ppm. ^{13}C NMR (75 MHz, $CDCl_3$) δ 158.5 (C), 155.7 (C), 150.1 (C), 144.7 (C), 142.42 (C), 142.40 (C), 139.1 (C), 137.5 (C), 134.5 (C), 133.9 (C), 131.9 (C), 131.8 (C), 130.7 (C), 129.9 (C), 129.3 (CH), 125.2 (C), 122.1 (CH), 121.2 (CH), 21.2 (CH_3), 19.4 (CH_3), 14.8 (CH_3), 14.6 (CH_3), 13.9 (CH_3), 13.6 (CH_3), 12.7 (CH_3), 11.1 (CH_3) ppm. FTIR ν 2921, 2852, 1544, 1511, 1469, 1406, 1309, 1192, 1158, 1077, 978 cm^{-1}. HRMS-EI *m/z* 612.3215 (612.3219 calcd. for $C_{35}H_{38}B_2F_4N_4$).

1c: According to general procedure 2.1., 2-formyl-8-(4-methoxyphenyl)-1,3,5,7-tetramethyl BODIPY[5] (100 mg, 0.26 mmol), 2,4-dimethylpyrrole (0.06 mL, 0.55 mmol) and TFA (two drops) in CH_2Cl_2 (20 mL) were reacted for 1 h. Then, a solution of DDQ (65 mg, 0.29 mmol)

in CH_2Cl_2 (10 mL), Et_3N (0.18 mL, 1.30 mmol) and $BF_3{\cdot}Et_2O$ (0.16 mL, 1.30 mmol) were added to the mixture. Flash chromatography using hexane/EtOAc (90:10) afforded **1c** (97 mg, 62%) as an orange solid. ^{1}H NMR (300 MHz, $CDCl_3$) δ 7.18 (d, J = 8.7 Hz, 2H, 2CH), 7.02 (d, J = 8.7 Hz, 2H, 2CH), 6.07 (s, 1H, CH), 5.98 (s, 2H, 2CH), 3.86 (s, 3H, OCH_3), 2.59 (s, 3H, CH_3), 2.53 (s, 6H, 2CH_3), 2.41 (s, 3H, CH_3), 1.71 (s, 6H, 2CH_3), 1.47 (s, 3H, CH_3), 1.26 (s, 3H, CH_3) ppm. ^{13}C NMR (75 MHz, $CDCl_3$) δ 160.4 (C), 158.5 (C), 155.7 (C), 150.4 (C), 145.5 (C), 142.6 (C), 142.5 (C), 138.3 (C), 133.8 (C), 132.9 (C), 131.9 (C), 131.4 (C), 129.1 (CH), 126.5 (C), 125.5 (C), 122.4 (CH), 121.2 (CH), 114.8 (CH), 55.4 (OCH_3), 14.82 (CH_3), 14.79 (CH_3), 14.6 (CH_3), 14.0 (CH_3), 12.7 (CH_3), 12.3 (CH_3) ppm. FTIR ν 2924, 2854, 1543, 1513, 1468, 1406, 1309, 1249, 1191, 1161, 1075, 978 cm^{-1}. HRMS-EI *m/z* 600.2852 (600.2855 calcd. for $C_{33}H_{34}B_2F_4N_4O$).

2a: According to general procedure 2.2., BODIPY **1a**[2] (30 mg, 0.06 mmol) and PCC (78 mg, 0.36 mmol) in EtOAc (8 mL) were reacted at rt for 24 h. Flash chromatography using hexane/EtOAc (99:1) afforded **2a** (17 mg, 54%) as a red solid. ^{1}H NMR (700 MHz, $CDCl_3$) δ 10.27 (s, 1H, CHO), 6.82 (s, 1H, CH), 6.27 (s, 1H, CH), 6.20 (s, 1H, CH), 2.69 (s, 3H, CH_3), 2.68 (s, 3H, CH_3), 2.58 (s, 3H, CH_3), 2.48 (s, 3H, CH_3), 2.38 (s, 3H, CH_3), 2.28 (s, 3H, CH_3), 1.78 (s, 3H, CH_3), 1.73 (s, 3H, CH_3) ppm. ^{13}C NMR (176 MHz, $CDCl_3$) δ 184.5 (CHO), 166.3 (C), 158.4 (C), 149.2 (C), 147.1 (C), 144.5 (C), 143.9 (C), 142.3 (C), 137.5 (C), 136.9 (C), 136.8 (C), 134.8 (C), 133.7 (C), 133.2 (C), 131.6 (C), 125.4 (CH), 123.8 (C), 123.3 (CH), 119.9 (CH), 17.7 (CH_3), 16.9 (CH_3), 15.7 (CH_3), 14.9 (CH_3), 14.7 (CH_3), 13.8 (CH_3), 12.7 (CH_3) ppm. FTIR ν 2924, 2855, 1670, 1552, 1480, 1402, 1314, 1197, 1144, 1078, 986 cm^{-1}. HRMS-EI *m/z* 522.2381 (522.2385 calcd. for $C_{27}H_{28}B_2F_4N_4O$).

2b: According to general procedure 2.2., dimer **1b** (60 mg, 0.10 mmol) and PCC (129 mg, 0.60 mmol) in EtOAc (8 mL) were reacted at rt for 7 h. Flash chromatography using hexane/EtOAc (70:30) afforded **2b** (40 mg, 64%) as a red solid. ^{1}H NMR (700 MHz, $CDCl_3$) δ 10.24 (s, 1H, CHO), 6.97 (s, 1H, CH), 6.96 (s, 1H, CH), 6.79 (s, 1H, CH), 6.24 (s, 1H, CH), 6.10 (s, 1H, CH), 2.66 (s, 3H, CH_3), 2.62 (s, 3H, CH_3), 2.41 (s, 3H, CH_3), 2.33 (s, 3H, CH_3), 2.11 (s, 3H, CH_3), 2.10 (s, 3H, CH_3), 1.78 (s, 3H, CH_3), 1.74 (s, 3H, CH_3), 1.43 (s, 3H, CH_3), 1.22 (s, 3H, CH_3) ppm. ^{13}C NMR (176 MHz, $CDCl_3$) δ 184.5 (CHO), 166.2 (C), 160.0 (C), 149.0 (C), 148.4 (C), 145.8 (C), 143.8 (C), 142.6 (C), 139.3 (C), 137.2 (C), 137.0 (C), 136.6 (C), 136.4 (C), 134.6 (C), 134.3 (C), 133.1 (C), 132.4 (C), 130.5 (C), 129.8 (C), 129.4 (CH), 129.3 (CH), 125.3 (CH), 123.7 (C), 122.8 (CH), 119.9 (CH), 21.3 (CH_3), 19.4 (CH_3), 15.7 (CH_3), 15.0 (CH_3), 14.6 (CH_3), 13.8 (CH_3), 13.7 (CH_3), 12.8 (CH_3), 11.1 (CH_3) ppm. FTIR ν 2922, 2854, 1670, 1542, 1401,

1311, 1188, 1143, 1115, 1083, 980 cm^{-1}. HRMS-EI *m/z* 626.3025 (626.3011 calcd. for $C_{35}H_{36}B_2F_4N_4O$).

2c: According to general procedure 2.2., dimer **1c** (30 mg, 0.05 mmol) and PCC (65 mg, 0.30 mmol) in EtOAc (8 mL) were reacted at rt for 20 h. Flash chromatography using hexane/EtOAc (85:15) afforded **2c** (17 mg, 55%) as a red solid. ^{1}H NMR (700 MHz, $CDCl_3$) δ 10.24 (s, 1H, CHO), 7.20-7.17 (m, 2H, 2CH), 7.04-7.03 (m, 2H, 2CH), 6.79 (s, 1H, CH), 6.24 (s, 1H, CH), 6.11 (s, 1H, CH), 3.87 (s, 3H, OCH_3), 2.66 (s, 3H, CH_3), 2.61 (s, 3H, CH_3), 2.41 (s, 3H, CH_3), 1.79 (s, 3H, CH_3), 1.75 (s, 3H, CH_3), 1.49 (s, 3H, CH_3), 1.26 (s, 3H, CH_3) ppm. ^{13}C NMR (176 MHz, $CDCl_3$) δ 184.5 (CHO), 166.3 (C), 160.5 (C), 160.0 (C), 149.1 (C), 148.6 (C), 146.6 (C), 143.8 (C), 142.8 (C), 137.3 (C), 137.2 (C), 136.9 (C), 136.6 (C), 133.4 (C), 133.1 (C), 131.3 (C), 129.1 (CH), 128.9 (CH), 126.2 (C), 125.3 (CH), 124.0 (C), 123.0 (CH), 119.9 (CH), 115.1 (CH), 114.8 (CH), 55.4 (OCH_3), 15.7 (CH_3), 15.0 (CH_3), 14.9 (CH_3), 14.7 (CH_3), 13.8 (CH_3), 12.8 (CH_3), 12.3 (CH_3) ppm. FTIR ν 2924, 2853, 1669, 1542, 1517, 1474, 1402, 1312, 1249, 1189, 1144, 1081, 1068, 988 cm^{-1}. HRMS-EI *m/z* 614.2638 (614.2648 calcd. for $C_{33}H_{32}B_2F_4N_4O_2$).

2d: According to general procedure 2.2., dimer **1d**[3] (30 mg, 0.05 mmol) and PCC (65 mg, 0.30 mmol) in EtOAc (8 mL) were reacted at rt for 8 h. Flash chromatography using hexane/EtOAc (85:15) afforded **2d** (18 mg, 57%) as a red solid. ^{1}H NMR (700 MHz, $CDCl_3$) δ 10.23 (s, 1H, CHO), 8.44-8.42 (m, 2H, 2CH), 7.59-7.56 (m, 2H, 2CH), 6.79 (s, 1H, CH), 6.26 (s, 1H, CH), 6.16 (s, 1H, CH), 2.66 (s, 3H, CH_3), 2.63 (s, 3H, CH_3), 2.43 (s, 3H, CH_3), 1.79 (s, 3H, CH_3), 1.74 (s, 3H, CH_3), 1.42 (s, 3H, CH_3), 1.21 (s, 3H, CH_3) ppm. ^{13}C NMR (176 MHz, $CDCl_3$) δ 184.4 (CHO), 166.7 (C), 161.6 (C), 150.1 (C), 148.7 (C), 148.6 (C), 145.9 (C), 144.0 (C), 141.1 (C), 139.2 (C), 137.1 (C), 136.7 (C), 136.5 (C), 135.9 (C), 132.9 (C), 132.4 (C), 129.9 (C), 129.5 (CH), 129.4 (CH), 125.5 (CH), 124.8 (CH), 124.7 (CH), 123.9 (CH), 120.0 (CH), 15.7 (CH_3), 15.08 (CH_3), 15.06 (CH_3), 14.7 (CH_3), 13.8 (CH_3), 12.9 (CH_3), 12.5 (CH_3) ppm. FTIR ν 2923, 2853, 1668, 1542, 1524, 1401, 1345, 1310, 1188, 1143, 1065, 979 cm^{-1}. HRMS-EI *m/z* 629.2386 (629.2392 calcd. for $C_{32}H_{29}B_2F_4N_5O_3$).

3a: According to general procedure 2.3., $POCl_3$ (0.17 mL, 1.80 mmol), DMF (0.28 mL, 3.60 mmol) and **1a**[2] (63 mg, 0.12 mmol) in DCE (10 mL) were reacted for 30 min. Flash chromatography using hexane/CH_2Cl_2 (10:90) afforded **3a** (49 mg, 76%) as an orange solid. ^{1}H NMR (300 MHz, $CDCl_3$) δ 10.03 (s, 1H, CHO), 6.19 (s, 2H, 2CH), 2.82 (s, 3H, CH_3), 2.67 (s, 3H, CH_3), 2.62 (s, 3H, CH_3), 2.56 (s, 3H, CH_3), 2.46 (s, 3H, CH_3), 2.36 (s, 3H, CH_3), 2.26 (s, 3H, CH_3), 2.00 (s, 3H, CH_3),1.72 (s, 3H, CH_3) ppm. ^{13}C NMR (75 MHz, $CDCl_3$) δ 186.3 (CHO),

162.4 (C), 158.5 (C), 157.1 (C), 147.7 (C), 147.2 (C), 144.8 (C), 142.8 (C), 136.7 (C), 135.4 (C), 134.0 (C), 132.0 (C), 130.6 (C), 126.7 (C), 124.5 (CH), 123.6 (CH), 18.1 (CH_3), 17.2 (CH_3), 15.6 (CH_3), 15.3 (CH_3), 15.1 (CH_3), 14.9 (CH_3), 13.5 (CH_3), 13.0 (CH_3), 11.3 (CH_3) ppm. FTIR ν 2964, 2852, 1669, 1541, 1312, 1192, 981 cm^{-1}. HRMS-EI *m/z* 536.2536 (536.2542 calcd. for $C_{28}H_{30}B_2F_4N_4O$).

3b: According to general procedure 2.3., $POCl_3$ (0.22 mL, 2.40 mmol), DMF (0.37 mL, 4.80 mmol) and **1b** (100.3 mg, 0.16 mmol) in DCE (10 mL) were reacted for 1 h. Flash chromatography using hexane/EtOAc (85:15) afforded **3b** (87 mg, 85%) as an orange solid. ^1H NMR (700 MHz, $CDCl_3$) δ 10.02 (s, 1H, CHO), 6.964 (s, 1H, CH), 6.959 (s, 1H, CH), 6.16 (s, 1H, CH), 6.09 (s, 1H, CH), 2.79 (s, 3H, CH_3), 2.61 (s, 3H, CH_3), 2.59 (s, 3H, CH_3), 2.40 (s, 3H, CH_3), 2.32 (s, 3H, CH_3), 2.11 (s, 3H, CH_3), 2.10 (s, 3H, CH_3), 2.01 (s, 3H, CH_3), 1.73 (s, 3H, CH_3), 1.43 (s, 3H, CH_3), 1.20 (s, 3H, CH_3) ppm. ^{13}C NMR (176 MHz, $CDCl_3$) δ 185.9 (CHO), 161.8 (C), 159.7 (C), 156.7 (C), 148.7 (C), 146.6 (C), 145.6 (C), 142.6 (C), 142.1 (C), 139.2 (C), 136.5 (C), 136.4 (C), 134.9 (C), 134.6 (C), 134.4 (C), 132.3 (C), 130.5 (C), 130.0 (C), 129.8 (C), 129.4 (CH), 129.3 (CH), 126.3 (C), 124.1 (CH), 122.7 (CH), 21.2 (CH_3), 19.4 (CH_3), 15.1 (CH_3), 14.9 (CH_3), 14.4 (CH_3), 13.7 (CH_3), 13.0 (CH_3), 12.7 (CH_3), 11.1 (CH_3), 10.9 (CH_3) ppm. FTIR ν 2924, 2852, 1673, 1541, 1438, 1406, 1311, 1186, 1072, 982 cm^{-1}. HRMS-EI *m/z* 640.3162 (640.3168 calcd. for $C_{36}H_{38}B_2F_4N_4O$).

3c: According to general procedure 2.3., $POCl_3$ (0.04 mL, 0.45 mmol), DMF (0.07 mL, 0.90 mmol) and **1c** (20 mg, 0.03 mmol) in DCE (10 mL) were reacted for 1 h. Flash chromatography using hexane/EtOAc (90:10) afforded **3c** (14 mg, 74%) as an orange solid. ^1H NMR (700 MHz, $CDCl_3$) δ 10.03 (s, 1H, CHO), 7.20-7.17 (m, 2H, 2CH), 7.04-7.02 (m, 2H, 2CH), 6.16 (s, 1H, CH), 6.11 (s, 1H, CH), 3.87 (s, 3H, OCH_3), 2.80 (s, 3H, CH_3), 2.604 (s, 3H, CH_3), 2.596 (s, 3H, CH_3), 2.40 (s, 3H, CH_3), 2.02 (s, 3H, CH_3), 1.75 (s, 3H, CH_3), 1.49 (s, 3H, CH_3), 1.24 (s, 3H, CH_3) ppm. ^{13}C NMR (176 MHz, $CDCl_3$) δ 186.0 (CHO), 161.9 (C), 160.5 (C), 159.8 (C), 156.7 (C), 148.9 (C), 146.7 (C), 146.4 (C), 142.8 (C), 142.2 (C), 137.4 (C), 136.3 (C), 134.9 (C), 133.3 (C), 131.3 (C), 130.0 (C), 129.1 (CH), 128.9 (CH), 126.29 (C), 126.27 (C), 124.3 (C), 124.1 (CH), 122.9 (CH), 115.0 (CH), 114.8 (CH), 55.4 (OCH_3), 15.2 (CH_3), 14.93 (CH_3), 14.91 (CH_3), 14.5 (CH_3), 13.1 (CH_3), 12.7 (CH_3), 12.3 (CH_3), 10.9 (CH_3) ppm. FTIR ν 2924, 2854, 1672, 1539, 1517, 1469, 1405, 1311, 1249, 1186, 1081, 1072, 981 cm^{-1}. HRMS-EI *m/z* 628.2798 (628.2804 calcd. for $C_{34}H_{34}B_2F_4N_4O_2$).

3d: According to general procedure 2.3., $POCl_3$ (0.07 mL, 0.75 mmol), DMF (0.12 mL, 1.50 mmol) and **1d**[3] (29 mg, 0.05 mmol) in DCE (10 mL) were reacted for 2 h. Flash chromatography

using hexane/EtOAc (90:10) afforded **3d** (24 mg, 75%) as an orange solid. ^{1}H NMR (700 MHz, $CDCl_3$) δ 10.03 (s, 1H, CHO), 8.45-8.40 (m, 2H, 2CH), 7.60-7.54 (m, 2H, 2CH), 6.18 (s, 1H, CH), 6.16 (s, 1H, CH), 2.79 (s, 3H, CH_3), 2.63 (s, 3H, CH_3), 2.60 (s, 3H, CH_3), 2.42 (s, 3H, CH_3), 2.02 (s, 3H, CH_3), 1.75 (s, 3H, CH_3), 1.42 (s, 3H, CH_3), 1.19 (s, 3H, CH_3) ppm. ^{13}C NMR (176 MHz, $CDCl_3$) δ 185.9 (CHO), 162.3 (C), 161.4 (C), 157.0 (C), 150.4 (C), 148.6 (C), 146.3 (C), 145.7 (C), 141.9 (C), 141.2 (C), 139.2 (C), 136.8 (C), 135.3 (C), 134.8 (C), 132.3 (C), 129.94 (C), 129.87 (C), 129.6 (CH), 129.4 (CH), 126.3 (C), 125.1 (C), 124.8 (CH), 124.7 (CH), 124.3 (CH), 123.8 (CH), 15.2 (CH_3), 15.1 (CH_3), 15.0 (CH_3), 14.5 (CH_3), 13.0 (CH_3), 12.8 (CH_3), 12.4 (CH_3), 11.0 (CH_3) ppm. FTIR ν 2925, 2854, 1672, 1539, 1524, 1468, 1405, 1310, 1185, 1165, 1070, 982 cm^{-1}. HRMS-EI *m/z* 643.2541 (643.2549 calcd. for $C_{33}H_{31}B_2F_4N_5O_3$).

4: According to general procedure 2.1., 2-formyl-8-mesityl-1,3,5,7-tetramethylBODIPY[4] (475 mg, 1.20 mmol), pyrrole (5 mL, 7.20 mmol) and TFA (two drops) in CH_2Cl_2 (20 mL) were reacted for 6 h. Then, a solution of DDQ (300 mg, 1.32 mmol) in CH_2Cl_2 (10 mL), Et_3N (0.83 mL, 6.00 mmol) and $BF_3{\cdot}Et_2O$ (0.74 mL, 6.00 mmol) were added to the mixture. Flash chromatography using hexane/EtOAc (90:10) afforded **4** (124 mg, 18%) as a red solid. ^{1}H-NMR (300 MHz, $CDCl_3$) δ 7.87 (broad s, 2H, 2CH), 6.97 (s, 2H, 2CH), 6.84 (d, J = 4.2 Hz, 2H, 2CH), 6.51-6.48 (m, 2H, 2CH), 6.11 (s, 1H, CH), 2.63 (s, 3H, CH_3), 2.49 (s, 3H, CH_3), 2.33 (s, 3H, CH_3), 2.15 (s, 6H, 2CH_3), 1.44 (s, 3H, CH_3), 1.29 (s, 3H, CH_3) ppm. ^{13}C NMR (75 MHz, $CDCl_3$) δ 159.6 (C), 151.4 (C), 145.5 (C), 144.0 (CH), 142.9 (C), 140.3 (C), 139.2 (C), 138.9 (C), 135.6 (C), 134.6 (C), 132.6 (C), 130.8 (CH), 130.6 (C), 129.8 (C), 129.4 (CH), 123.9 (C), 122.9 (CH), 118.5 (CH), 21.2 (CH_3), 19.7 (CH_3), 15.0 (CH_3), 13.8 (CH_3), 13.6 (CH_3), 12.3 (CH_3) ppm. FTIR ν 2922, 2850, 1541, 1509, 1471, 1402, 1305, 1193, 1160, 1074, 975 cm^{-1}. HRMS-EI *m/z* 556.2589 (556.2593 calcd. for $C_{31}H_{30}B_2F_4N_4$).

5: According to general procedure 2.2., dimer **4** (44 mg, 0.08 mmol) and PCC (103 mg, 0.48 mmol) in DCE (5 mL) were reacted at 60 ºC for 4 h. Flash chromatography using hexane/EtOAc (85:15) afforded **5a** (11 mg, 24%) as a red solid, and **5b** (5 mg, 11%) as an orange solid.

5a: ^{1}H-NMR (700 MHz, $CDCl_3$) δ 10.34 (s, 1H, CHO), 7.93 (broad s, 2H, 2CH), 7.03 (s, 2H, 2CH), 6.85 (s, 1H, CH), 6.81 (d, J = 4.2 Hz, 2H, 2CH), 6.55-6.54 (m, 2H, 2CH), 2.60 (s, 3H, CH_3), 2.35 (s, 3H, CH_3), 2.15 (s, 6H, 2CH_3), 1.49 (s, 3H, CH_3), 1.39 (s, 3H, CH_3) ppm. ^{13}C NMR (176 MHz, $CDCl_3$) δ 184.6 (CHO), 161.6 (C), 147.8 (C), 146.2 (C), 145.8 (C), 145.5 (CH), 140.5 (C), 140.1 (C), 137.2 (C), 135.1 (C), 134.1 (C), 133.9 (C), 132.9 (C), 130.5 (CH), 129.83 (CH), 129.78 (C), 127.9 (C), 120.3 (CH), 119.3 (CH), 21.3 (CH_3), 19.8 (CH_3), 14.5

(CH_3), 13.7 (CH_3), 13.1 (CH_3) ppm. FTIR ν 2922, 2850, 1668, 1539, 1405, 1309, 1183, 1147, 1079, 977 cm^{-1}. HRMS-EI *m/z* 570.2380 (570.2385 calcd. for $C_{31}H_{28}B_2F_4N_4O$).

5b: ^{1}H-NMR (700 MHz, $CDCl_3$) δ 10.23 (s, 1H, CHO), 7.86 (broad s, 2H, 2CH), 7.00 (s, 2H, 2CH), 6.73 (d, *J* = 4.2 Hz, 2H, 2CH), 6.45-6.44 (m, 2H, 2CH), 6.33 (s, 1H, CH), 2.74 (s, 3H, CH_3), 2.34 (s, 3H, CH_3), 2.14 (s, 6H, 2CH_3), 1.52 (s, 3H, CH_3), 1.24 (s, 3H, CH_3) ppm. ^{13}C NMR (176 MHz, $CDCl_3$) δ 182.6 (CHO), 168.3 (C), 150.3 (C), 144.2 (CH), 143.9 (C), 140.8 (C), 140.2 (C), 140.0 (C), 136.5 (C), 135.6 (C), 134.4 (C), 131.2 (C), 130.1 (CH), 129.7 (CH), 129.6 (C), 126.1 (CH), 125.4 (C), 118.4 (CH), 21.3 (CH_3), 19.8 (CH_3), 15.9 (CH_3), 14.3 (CH_3), 11.1 (CH_3) ppm. FTIR ν 2922, 2851, 1669, 1542, 1400, 1312, 1180, 1141, 1089, 977 cm^{-1}. HRMS-EI *m/z* 570.2379 (570.2385 calcd. for $C_{31}H_{28}B_2F_4N_4O$).

6a: According to general procedure 2.1., 3-formyl-1,5,7,8-tetramethylBODIPY[6] (69 mg, 0.25 mmol), 2,4-dimethylpyrrole (0.05 mL, 0.53 mmol) and TFA (two drops) in CH_2Cl_2 (15 mL) were reacted for 1 h. Then, a solution of DDQ (62 mg, 0.28 mmol) in CH_2Cl_2 (10 mL), Et_3N (0.17 mL 1.25 mmol) and $BF_3{\cdot}Et_2O$ (0.15 mL, 1.25 mmol) were added to the mixture. Flash chromatography using hexane/EtOAc (80:20) afforded **6a** (14 mg, 11%) as a red solid. ^{1}H NMR (700 MHz, $CDCl_3$) δ 6.18 (s, 1H, CH), 6.15 (s, 1H, CH), 5.98 (s, 2H, 2CH), 2.72 (s, 3H, CH_3), 2.54 (s, 6H, 2CH_3), 2.53 (s, 3H, CH_3), 2.47 (s, 3H, CH_3), 2.44 (s, 3H, CH_3), 1.69 (s, 6H, 2CH_3) ppm. ^{13}C NMR (176 MHz, $CDCl_3$) δ 159.3 (C), 156.0 (C), 144.8 (C), 143.4 (C), 143.3 (C), 142.6 (C), 138.4 (C), 133.9 (C), 132.0 (C), 131.4 (C), 131.2 (C), 123.5 (CH), 120.9 (CH), 119.9 (CH), 17.8 (CH_3), 17.3 (CH_3), 17.0 (CH_3), 14.9 (CH_3), 14.8 (CH_3), 13.1 (CH_3) ppm. FTIR ν 2923, 2854, 1548, 1511, 1410, 1313, 1190, 1156, 1062, 980 cm^{-1}. HRMS-EI *m/z* 494.2430 (494.2436 calcd. for $C_{26}H_{28}B_2F_4N_4$).

6b: According to general procedure 2.1., 3-formyl-8-mesityl-1,5,7-trimethylBODIPY[6] (175 mg, 0.46 mmol), 2,4-dimethylpyrrole (0.10 mL, 0.97 mmol) and TFA (two drops) in CH_2Cl_2 (20 mL) were reacted for 1 h. Then, a solution of DDQ (116 mg, 0.51 mmol) in CH_2Cl_2 (10 mL), Et_3N (0.32 mL, 2.30 mmol) and $BF_3{\cdot}Et_2O$ (0.28 mL, 2.30 mmol) were added to the mixture. Flash chromatography using hexane/EtOAc (85:15) afforded **6b** (61 mg, 22%) as a red solid. ^{1}H NMR (300 MHz, $CDCl_3$) δ 7.01 (s, 2H, 2CH), 6.08 (s, 1H, CH), 6.06 (s, 1H, CH), 6.00 (s, 2H, 2CH), 2.55 (s, 6H, 2CH_3), 2.48 (s, 3H, CH_3), 2.37 (s, 3H, CH_3), 2.13 (s, 6H, 2CH_3), 1.77 (s, 6H, 2CH_3), 1.47 (s, 3H, CH_3), 1.44 (s, 3H, CH_3) ppm. ^{13}C NMR (75 MHz, $CDCl_3$) δ 161.0 (C), 156.1 (C), 146.1 (C), 143.9 (C), 143.5 (C), 143.3 (C), 139.9 (C), 139.2 (C), 134.5 (C), 132.7 (C), 132.0 (C), 131.2 (C), 130.8 (C), 129.8 (C), 129.3 (CH), 123.1 (CH), 121.0 (CH), 119.4 (CH), 21.3 (CH_3), 19.3 (CH_3), 15.1 (CH_3), 14.8 (CH_3), 13.8 (CH_3), 13.3 (CH_3), 12.8

(CH_3) ppm. FTIR ν 2924, 2854, 1544, 1510, 1407, 1303, 1191, 1156, 1065, 982 cm^{-1}. HRMS-EI *m/z* 598.3060 (598.3062 calcd. for $C_{34}H_{36}B_2F_4N_4$).

7a: According to general procedure 2.2., BODIPY **6a** (14 mg, 0.03 mmol) and PCC (39 mg, 0.18 mmol) in EtOAc (8 mL) were reacted for 18 h. Flash chromatography using hexane/EtOAc (75:25) afforded **7a** (7 mg, 46%) as a red solid. ^1H NMR (700 MHz, $CDCl_3$) δ 10.25 (s, 1H, CHO), 6.79 (s, 1H, CH), 6.22 (s, 1H, CH), 6.20 (s, 1H, CH), 6.19 (s, 1H, CH), 2.74 (s, 3H, CH_3), 2.67 (s, 3H, CH_3), 2.54 (s, 3H, CH_3), 2.49 (s, 3H, CH_3), 2.44 (s, 3H, CH_3), 1.80 (s, 3H, CH_3) 1.70 (s, 3H, CH_3) ppm. ^{13}C NMR (176 MHz, $CDCl_3$) δ 184.7 (CHO), 167.5 (C), 160.8 (C), 150.7 (C), 146.0 (C), 143.8 (C), 143.6 (C), 140.1 (C), 138.1 (C), 137.9 (C), 137.2 (C), 134.5 (C), 133.8 (C), 133.5 (C), 131.6 (C), 125.2 (CH), 124.2 (CH), 119.7 (CH), 119.2 (CH), 18.0 (CH_3), 17.4 (CH_3), 17.1 (CH_3), 16.0 (CH_3), 15.0 (CH_3), 13.84 (CH_3), 13.82 (CH_3), 13.00 (CH_3), 12.98 (CH_3) ppm. FTIR ν 2920, 2853, 1671, 1548, 1510, 1412, 1303, 1190, 1146, 980 cm^{-1}. HRMS-EI *m/z* 508.2223 (508.2229 calcd. for $C_{26}H_{26}B_2F_4N_4O$).

7b: According to general procedure 2.2., BODIPY **6b** (30 mg, 0.05 mmol) and PCC (65 mg, 0.30 mmol) in EtOAc (8 mL) were reacted for 6 h. Flash chromatography using hexane/EtOAc (85:15) afforded **7b** (12 mg, 39%) as a red solid. ^1H NMR (700 MHz, $CDCl_3$) δ 10.26 (s, 1H, CHO), 7.02 (s, 2H, 2CH), 6.81 (s, 1H, CH), 6.25 (s, 1H, CH), 6.11 (s, 1H, CH), 6.10 (s, 1H, CH), 2.68 (s, 3H, CH_3), 2.48 (s, 3H, CH_3), 2.37 (s, 3H, CH_3), 2.13 (s, 3H, CH_3), 2.11 (s, 3H, CH_3), 1.87 (s, 3H, CH_3), 1.77 (s, 3H, CH_3), 1.48 (s, 3H, CH_3), 1.46 (s, 3H, CH_3) ppm. ^{13}C NMR (176 MHz, $CDCl_3$) δ 184.6 (CHO), 167.4 (C), 162.3 (C), 150.4 (C), 147.1 (C), 143.70 (C), 143.67 (C), 141.3 (C), 139.6 (C), 139.4 (C), 137.7 (C), 137.0 (C), 134.4 (C), 134.3 (C), 133.7 (C), 133.3 (C), 133.2 (C), 130.5 (C), 129.8 (C), 129.5 (CH), 129.4 (CH), 125.2 (CH), 123.6 (CH), 119.6 (CH), 118.7 (CH), 21.3 (CH_3), 19.4 (CH_3), 19.3 (CH_3), 15.9 (CH_3), 15.1 (CH_3), 13.9 (CH_3), 13.43 (CH_3), 13.41 (CH_3), 13.3 (CH_3), 12.56 (CH_3), 12.55 (CH_3) ppm. FTIR ν 2921, 2851, 1668, 1546, 1405, 1304, 1193, 1144, 1090, 986 cm^{-1}. HRMS-EI *m/z* 612.2847 (612.2855 calcd. for $C_{34}H_{34}B_2F_4N_4O$).

8a: According to general procedure 2.3., $POCl_3$ (0.04 mL, 0.45 mmol), DMF (0.07 mL, 0.90 mmol) and **6a** (15 mg, 0.03 mmol) in DCE (5 mL) were reacted for 1 h. Flash chromatography using hexane/EtOAc (70:30) afforded **8a** (10 mg, 64%) as an orange solid. ^1H NMR (700 MHz, $CDCl_3$) δ 10.00 (s, 1H, CHO), 6.19 (s, 1H, CH), 6.18 (s, 1H, CH), 6.15 (s, 1H, CH), 2.81 (s, 3H, CH_3), 2.74 (s, 3H, CH_3), 2.61 (s, 3H, CH_3), 2.54 (s, 3H, CH_3), 2.49 (s, 3H, CH_3), 2.44 (s, 3H, CH_3), 1.94 (s, 3H, CH_3), 1.75 (s, 3H, CH_3) ppm. ^{13}C NMR (176 MHz, $CDCl_3$) δ 186.1 (CHO), 162.7 (C), 160.3 (C), 156.6 (C), 147.9 (C), 145.7 (C), 143.5 (C), 143.0 (C), 140.6 (C),

138.1 (C), 135.1 (C), 134.3 (C), 133.3 (C), 131.4 (C), 130.2 (C), 126.0 (C), 124.0 (CH), 123.8 (CH), 119.4 (CH), 17.8 (CH_3), 17.3 (CH_3), 17.0 (CH_3), 15.3 (CH_3), 14.9 (CH_3), 13.5 (CH_3), 13.2 (CH_3), 10.6 (CH_3) ppm. FTIR ν 2921, 2851, 1669, 1557, 1544, 1466, 1314, 1192, 1088, 988 cm^{-1}. HRMS-EI *m/z* 522.2379 (522.2385 calcd. for $C_{27}H_{28}B_2F_4N_4O$).

8b: According to general procedure 2.3., $POCl_3$ (0.21 mL, 2.25 mmol), DMF (0.35 mL, 4.50 mmol) and **6b** (88 mg, 0.15 mmol) in DCE (10 mL) were reacted for 1 h. Flash chromatography using hexane/EtOAc (85:15) afforded **8b** (73 mg, 78%) as an orange solid. ^1H NMR (700 MHz, $CDCl_3$) δ 10.02 (s, 1H, CHO), 7.02 (s, 1H, CH), 7.01 (s, 1H, CH), 6.17 (s, 1H, CH), 6.093 (s, 1H, CH), 6.087 (s, 1H, CH), 2.82 (s, 3H, CH_3), 2.62 (s, 3H, CH_3), 2.48 (s, 3H, CH_3), 2.37 (s, 3H, CH_3), 2.13 (s, 3H, CH_3), 2.12 (s, 3H, CH_3), 2.01 (s, 3H, CH_3), 1.83 (s, 3H, CH_3), 1.47 (s, 3H, CH_3), 1.45 (s, 3H, CH_3) ppm. ^{13}C NMR (176 MHz, $CDCl_3$) δ 186.1 (CHO), 162.7 (C), 161.9 (C), 156.7 (C), 147.8 (C), 146.9 (C), 143.7 (C), 142.8 (C), 141.9 (C), 139.7 (C), 139.3 (C), 135.0 (C), 134.5 (C), 134.3 (C), 133.2 (C), 133.1 (C), 130.5 (C), 130.1 (C), 129.7 (C), 129.4 (CH), 129.3 (CH), 126.1 (C), 123.9 (CH), 123.5 (CH), 119.0 (CH), 21.3 (CH_3), 19.4 (CH_3), 19.3 (CH_3), 15.3 (CH_3), 15.1 (CH_3), 13.9 (CH_3), 13.3 (CH_3), 13.24 (CH_3), 13.22 (CH_3), 13.16 (CH_3), 10.26 (CH_3), 10.25 (CH_3) ppm. FTIR ν 2922, 2852, 1672, 1542, 1519, 1403, 1305, 1184, 1158, 1108, 984 cm^{-1}. HRMS-EI *m/z* 626.3009 (626.3011 calcd. for $C_{35}H_{36}B_2F_4N_4O$).

9: According to general procedure 2.1., dimer **2b** (81 mg, 0.13 mmol), 2,4-dimethylpyrrole (0.28 mL, 0.27 mmol) and TFA (two drops) in CH_2Cl_2 (20 mL) were reacted for 1 h. Then, a solution of DDQ (32 mg, 0.14 mmol) in CH_2Cl_2 (10 mL), Et_3N (0.09 mL, 0.65 mmol) and $BF_3{\cdot}Et_2O$ (0.08 mL, 0.65 mmol) were added to the mixture. Flash chromatography using hexane/EtOAc (90:10 to 70:30) afforded trimer **9** (8 mg, 7%) as an orange solid. ^1H NMR (700 MHz, $CDCl_3$) δ 6.98 (s, 2H, 2CH), 6.10 (s, 1H, CH), 6.09 (s, 1H, CH), 6.08 (s, 1H, CH), 6.01 (s, 1H, CH), 5.96 (s, 1H, CH), 2.62 (s, 3H, CH_3), 2.54 (s, 3H, CH_3), 2.53 (s, 3H, CH_3), 2.46 (s, 3H, CH_3), 2.43 (s, 3H, CH_3), 2.34 (s, 3H, CH_3), 2.13 (s, 3H, CH_3), 2.11 (s, 3H, CH_3), 1.78 (s, 3H, CH_3), 1.75 (s, 3H, CH_3), 1.67 (s, 3H, CH_3), 1.44 (s, 3H, CH_3), 1.25 (s, 3H, CH_3) ppm. ^{13}C NMR (176 MHz, $CDCl_3$) δ 161.5 (C), 159.5 (C), 156.4 (C), 156.0 (C), 148.8 (C), 146.1 (C), 145.4 (C), 144.3 (C), 143.4 (C), 143.0 (C), 142.5 (C), 140.0 (C), 139.2 (C), 136.5 (C), 136.0 (C), 134.6 (C), 134.5 (C), 134.0 (C), 132.2 (C), 131.9 (C), 131.8 (C), 130.9 (C), 130.8 (C), 130.6 (C), 129.9 (C), 129.3 (CH), 124.4 (C), 123.5 (C), 122.6 (CH), 121.2 (CH), 120.9 (CH), 119.8 (CH), 21.3 (CH_3), 19.5 (CH_3), 19.4 (CH_3), 15.1 (CH_3), 15.0 (CH_3), 14.8 (CH_3), 14.2 (CH_3), 14.1 (CH_3), 13.8 (CH_3), 13.7 (CH_3), 12.8 (CH_3), 12.7 (CH_3), 12.5 (CH_3), 10.9 (CH_3)

ppm. FTIR ν 2924, 2854, 1543, 1468, 1403, 1308, 1189, 1157, 1068, 982 cm^{-1}. HRMS-MALDI-TOF *m/z* 844.4208 (844.4202 calcd. for $C_{47}H_{49}B_3F_6N_6$).

10: According to general procedure 2.1., dimer **3b** (90 mg, 0.14 mmol), 2,4-dimethylpyrrole (0.03 mL, 0.29 mmol) and TFA (two drops) in CH_2Cl_2 (20 mL) were reacted for 30 min. Then, a solution of DDQ (34 mg, 0.15 mmol) in CH_2Cl_2 (10 mL), Et_3N (0.10 mL, 0.70 mmol) and $BF_3{\cdot}Et_2O$ (0.09 mL, 0.70 mmol) were added to the mixture. Flash chromatography using hexane/CH_2Cl_2/EtOAc (70:20:10) afforded trimer **10** (61 mg, 51%) as an orange solid. ^1H NMR (700 MHz, $CDCl_3$) δ 6.96 (s, 2H, 2CH), 6.08 (s, 1H, CH), 6.07 (s, 1H, CH), 6.02 (s, 1H, CH), 5.98 (s, 1H, CH), 2.59 (s, 3H, CH_3), 2.58 (s, 3H, CH_3), 2.542 (s, 3H, CH_3), 2.537 (s, 3H, CH_3), 2.40 (s, 3H, CH_3), 2.39 (s, 3H, CH_3), 2.33 (s, 3H, CH_3), 2.10 (s, 3H, CH_3), 2.04 (s, 3H, CH_3), 1.72 (s, 3H, CH_3), 1.70 (s, 3H, CH_3), 1.60 (s, 3H, CH_3), 1.54 (s, 3H, CH_3), 1.40 (s, 3H, CH_3), 1.22 (s, 3H, CH_3) ppm. ^{13}C NMR (176 MHz, $CDCl_3$) δ 159.4 (C), 158.9 (C), 156.3 (C), 155.4 (C), 150.7 (C), 148.9 (C), 145.4 (C), 144.8 (C), 142.7 (C), 142.5 (C), 142.0 (C), 139.1 (C), 137.6 (C), 136.5 (C), 134.8 (C), 134.6 (C), 134.4 (C), 133.5 (C), 133.1 (C), 132.2 (C), 132.0 (C), 131.7 (C), 131.1 (C), 130.6 (C), 129.8 (C), 129.4 (CH), 129.3 (CH), 125.6 (C), 124.4 (C), 122.6 (CH), 121.6 (CH), 121.1 (CH), 21.3 (CH_3), 19.43 (CH_3), 19.41 (CH_3), 14.93 (CH_3), 14.87 (CH_3), 14.7 (CH_3), 14.6 (CH_3), 14.1 (CH_3), 13.9 (CH_3), 13.8 (CH_3), 13.7 (CH_3), 12.7 (CH_3), 12.5 (CH_3), 11.5 (CH_3), 10.9 (CH_3) ppm. FTIR ν 2925, 2854, 1545, 1468, 1408, 1311, 1192, 1083, 980 cm^{-1}. HRMS-MALDI-TOF *m/z* 858.4349 (858.4359 calcd. for $C_{48}H_{51}B_3F_6N_6$).

11: According to general procedure 2.1., dimer **7b** (154 mg, 0.25 mmol), 2,4-dimethylpyrrole (0.05 mL, 0.53 mmol) and TFA (two drops) in CH_2Cl_2 (20 mL) were reacted for 30 min. Then, a solution of DDQ (63 mg, 0.28 mmol) in CH_2Cl_2 (10 mL), Et_3N (0.17 mL, 1.25 mmol) and $BF_3{\cdot}Et_2O$ (0.15 mL, 1.25 mmol) were added to the mixture. Flash chromatography using hexane/CH_2Cl_2/EtOAc (80:10:10) afforded trimer **11** (10 mg, 5%) as an orange solid. ^1H NMR (700 MHz, $CDCl_3$) δ 7.02 (s, 2H, 2CH), 6.20 (s, 1H, CH), 6.102 (s, 1H, CH), 6.095 (s, 1H, CH), 6.09 (s, 1H, CH), 5.99 (s, 2H, 2CH), 2.55 (s, 6H, 2CH_3), 2.49 (s, 3H, CH_3), 2.48 (s, 3H, CH_3), 2.37 (s, 3H, CH_3), 2.15 (s, 3H, CH_3), 2.11 (s, 3H, CH_3), 1.85 (s, 3H, CH_3), 1.82 (s, 6H, 2CH_3), 1.81 (s, 3H, CH_3), 1.50 (s, 3H, CH_3), 1.45 (s, 3H, CH_3) ppm. ^{13}C NMR (176 MHz, $CDCl_3$) δ 162.1 (C), 161.6 (C), 156.2 (C), 155.7 (C), 147.2 (C), 146.6 (C), 144.6 (C), 144.1 (C), 143.6 (C), 143.0 (C), 142.5 (C), 140.9 (C), 139.5 (C), 139.3 (C), 134.41 (C), 134.38 (C), 134.1 (C), 133.1 (C), 132.9 (C), 132.3 (C), 131.6 (C), 131.5 (C), 131.2 (C), 130.6 (C), 129.9 (C), 129.4 (CH), 129.3 (CH), 123.3 (CH), 123.2 (CH), 121.1 (CH), 120.7 (CH), 119.3 (CH), 118.9 (CH), 21.3 (CH_3), 19.4 (CH_3), 19.2 (CH_3), 15.3 (CH_3), 15.0 (CH_3), 14.81 (CH_3), 14.76 (CH_3), 13.9

(CH_3), 13.34 (CH_3), 13.26 (CH_3), 13.2 (CH_3), 12.8 (CH_3), 10.8 (CH_3) ppm. FTIR ν 2924, 2852, 1544, 1304, 1187, 1156, 984 cm^{-1}. HRMS-MALDI-TOF *m/z* 830.4038 (830.4046 calcd. for $C_{46}H_{47}B_3F_6N_6$).

12: According to general procedure 2.1., dimer **8b** (75 mg, 0.12 mmol), 2,4-dimethylpyrrole (0.03 mL, 0.25 mmol) and TFA (two drops) in CH_2Cl_2 (20 mL) were reacted for 30 min. Then, a solution of DDQ (29 mg, 0.13 mmol) in CH_2Cl_2 (10 mL), Et_3N (0.08 mL, 0.60 mmol) and $BF_3{\cdot}Et_2O$ (0.07 mL, 0.60 mmol) were added to the mixture. Flash chromatography using hexane/EtOAc (95:5 to 85:15) afforded trimer **12** (32 mg, 32%) as an orange solid. ^1H NMR (700 MHz, $CDCl_3$) δ 6.99 (s, 1H, CH), 6.98 (s, 1H, CH), 6.092 (s, 1H, CH), 6.088 (s, 1H, CH), 6.07 (s, 1H, CH), 5.98 (s, 1H, CH), 5.96 (s, 1H, CH), 2.60 (s, 3H, CH_3), 2.52 (s, 6H, $2CH_3$), 2.47 (s, 3H, CH_3), 2.42 (s, 3H, CH_3), 2.35 (s, 3H, CH_3), 2.12 (s, 3H, CH_3), 2.01 (s, 3H, CH_3), 1.83 (s, 3H, CH_3), 1.81 (s, 3H, CH_3), 1.71 (s, 3H, CH_3), 1.56 (s, 3H, CH_3), 1.45 (s, 3H, CH_3), 1.42 (s, 3H, CH_3) ppm. ^{13}C NMR (176 MHz, $CDCl_3$) δ 161.4 (C), 159.4 (C), 156.0 (C), 154.9 (C), 150.4 (C), 146.5 (C), 145.8 (C), 144.2 (C), 143.6 (C), 142.8 (C), 142.1 (C), 139.8 (C), 139.2 (C), 138.6 (C), 134.4 (C), 134.3 (C), 134.1 (C), 133.1 (C), 132.9 (C), 132.2 (C), 132.0 (C), 131.7 (C), 131.6 (C), 130.6 (C), 129.8 (C), 129.4 (CH), 129.3 (CH), 125.3 (C), 123.3 (CH), 122.2 (CH), 121.3 (CH), 120.9 (CH), 119.0 (CH), 21.3 (CH_3), 19.3 (CH_3), 19.1 (CH_3), 15.04 (CH_3), 14.99 (CH_3), 14.7 (CH_3), 14.60 (CH_3), 14.1 (CH_3), 13.9 (CH_3), 13.82 (CH_3), 13.79 (CH_3), 13.3 (CH_3), 13.07 (CH_3), 13.05 (CH_3), 12.8 (CH_3), 10.68 (CH_3), 10.67 (CH_3) ppm. FTIR ν 2923, 2853, 1543, 1512, 1467, 1305, 1190, 1158, 1067, 980 cm^{-1}. HRMS-MALDI-TOF *m/z* 844.4208 (844.4202 calcd. for $C_{47}H_{49}B_3F_6N_6$).

3. ^{1}H NMR and ^{13}C NMR spectra

^{1}H NMR (300 MHz, $CDCl_3$) and ^{13}C NMR (75 MHz, $CDCl_3$) spectra of **1b**

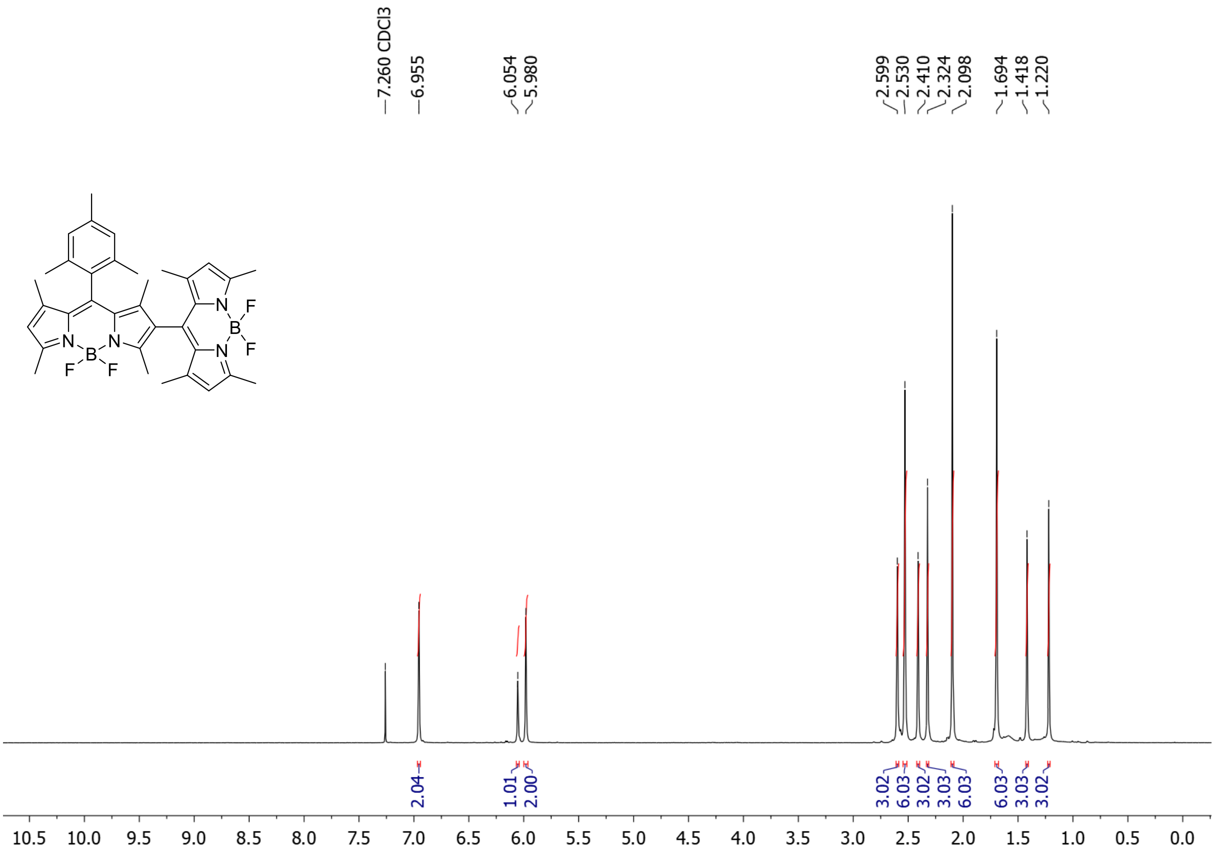

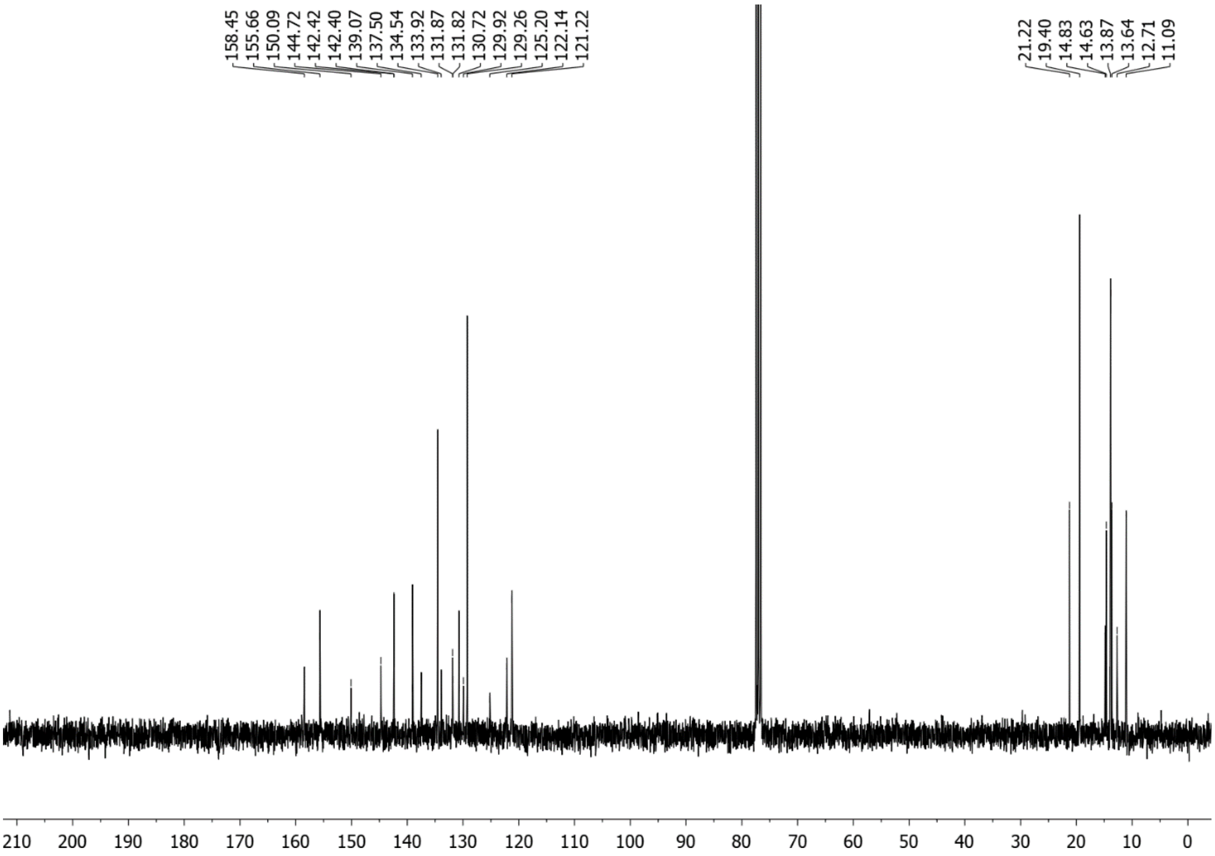

^{1}H NMR (300 MHz, $CDCl_3$) and ^{13}C NMR (75 MHz, $CDCl_3$) spectra of **1c**

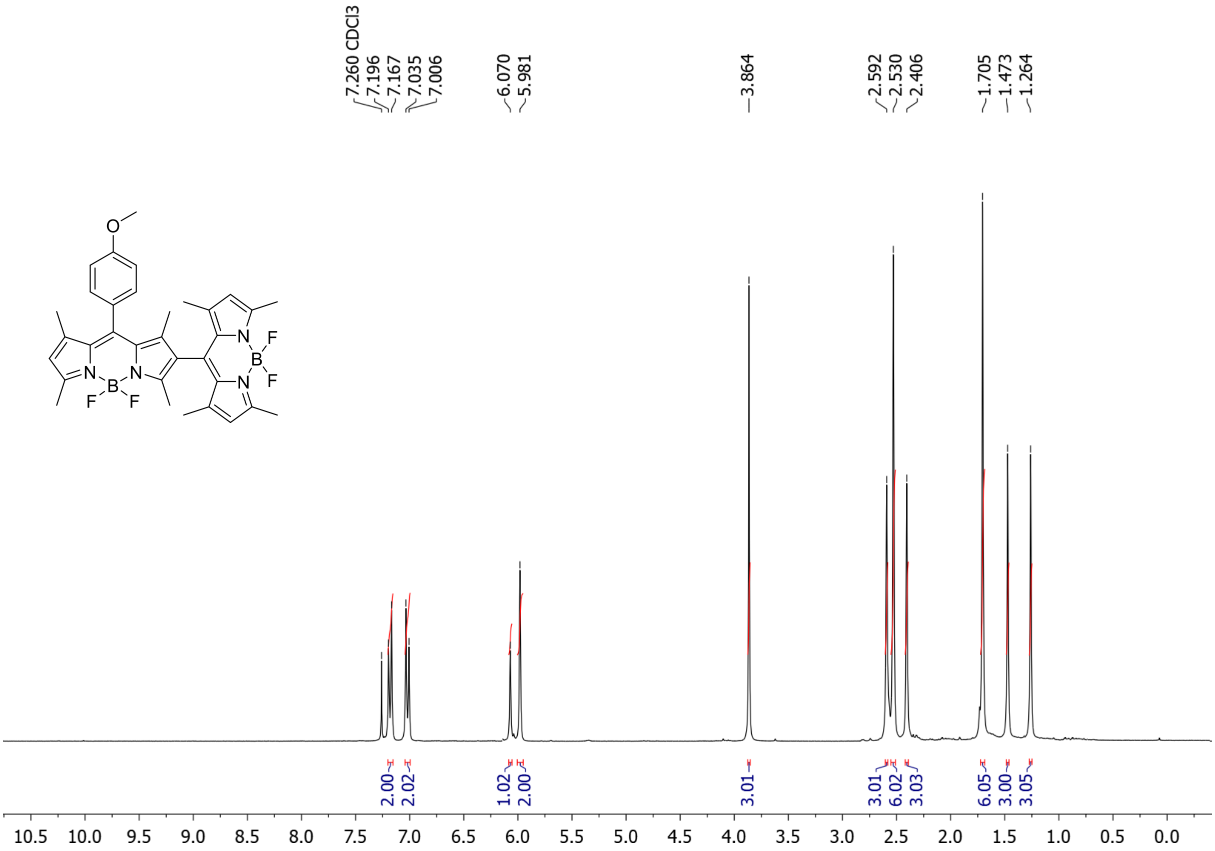

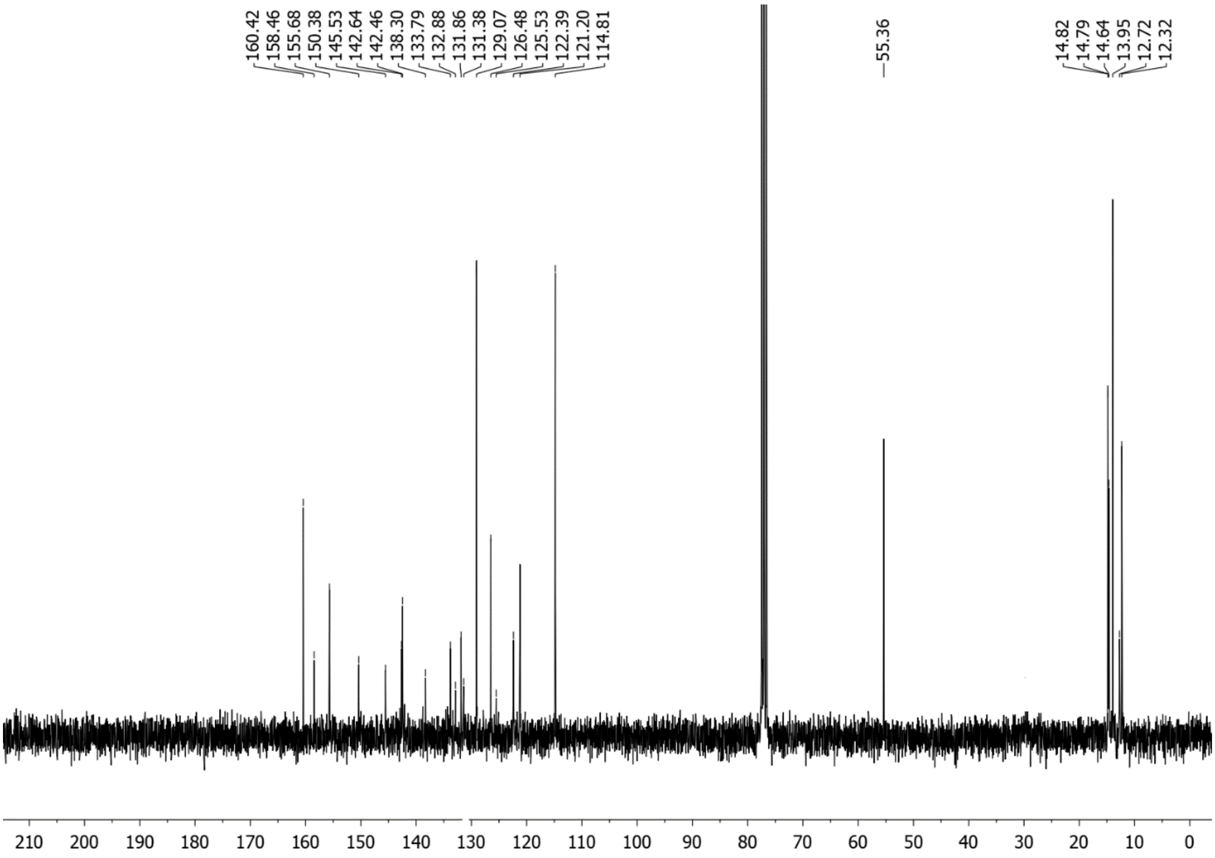

^{1}H NMR (700 MHz, $CDCl_3$) and ^{13}C NMR (176 MHz, $CDCl_3$) spectra of **2a**

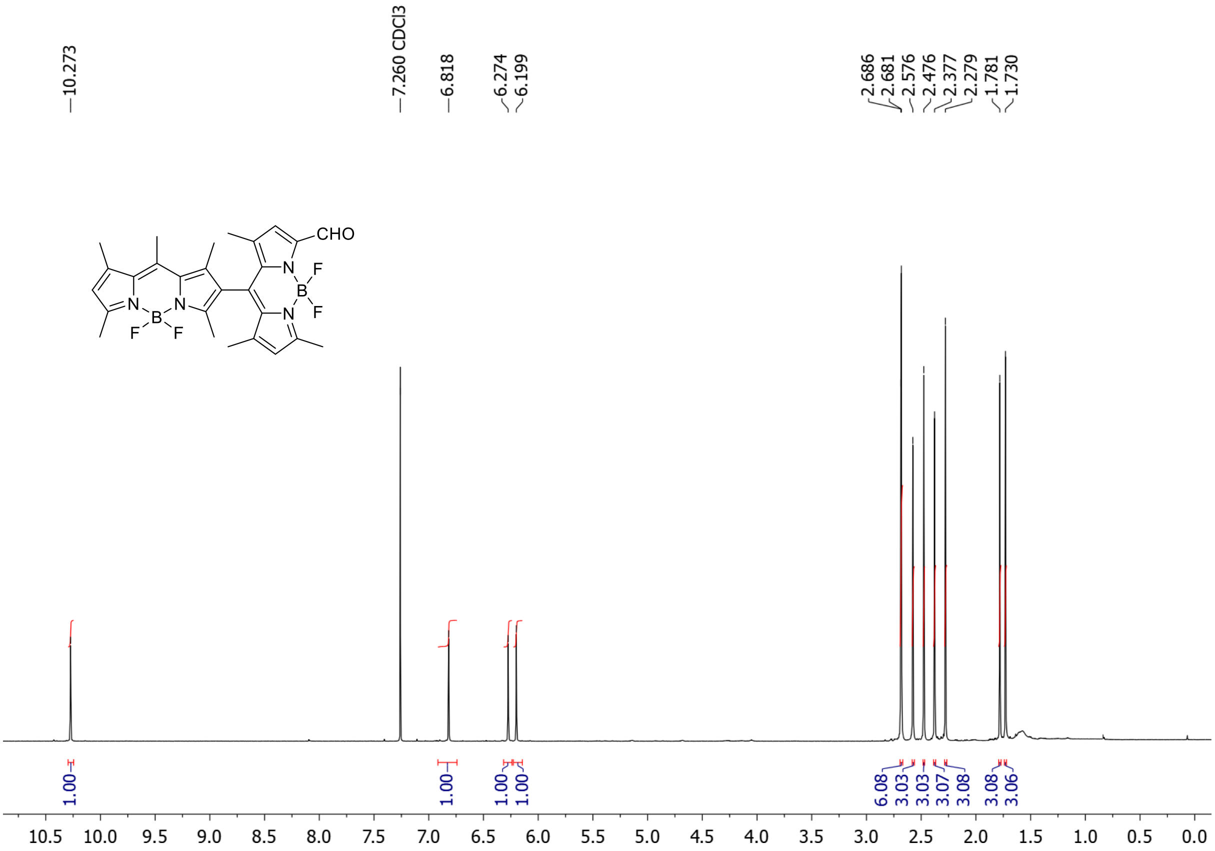

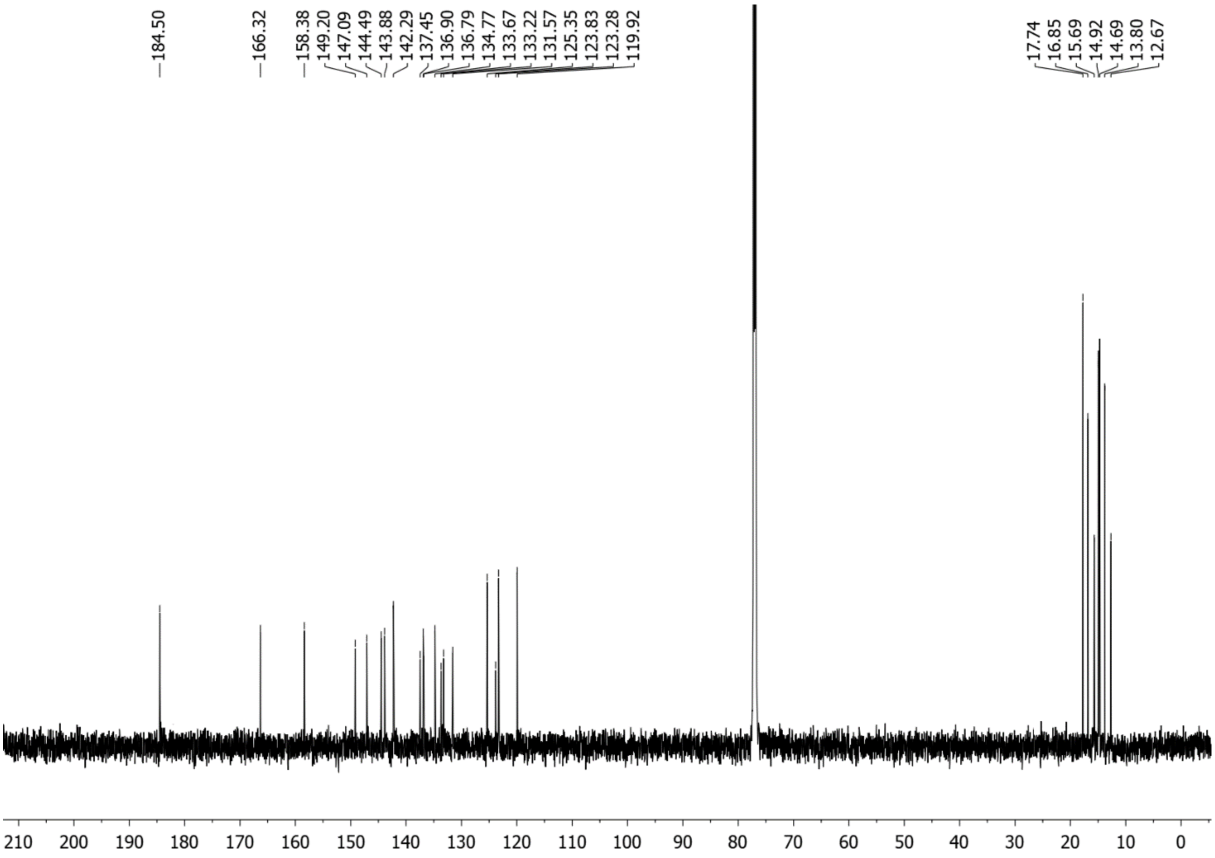

^{1}H NMR (700 MHz, $CDCl_3$) and ^{13}C NMR (176 MHz, $CDCl_3$) spectra of **2b**

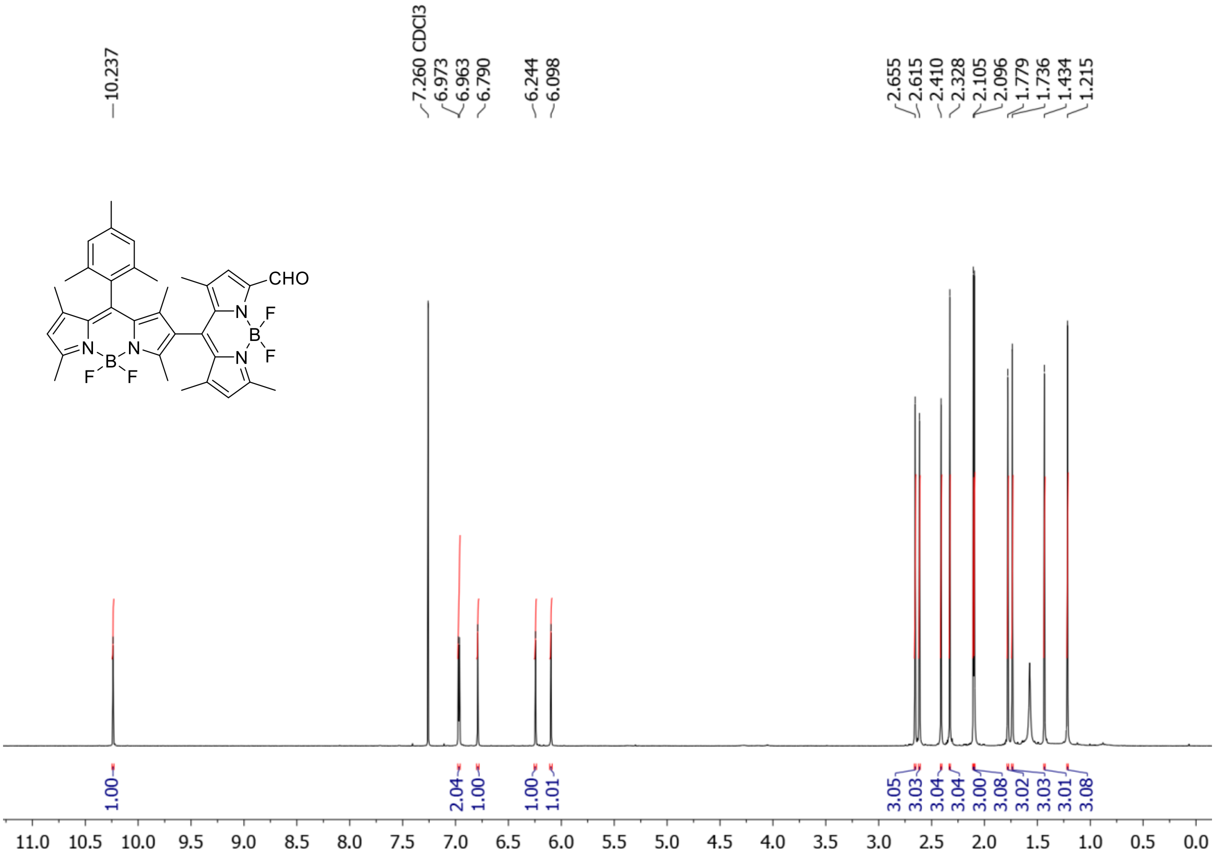

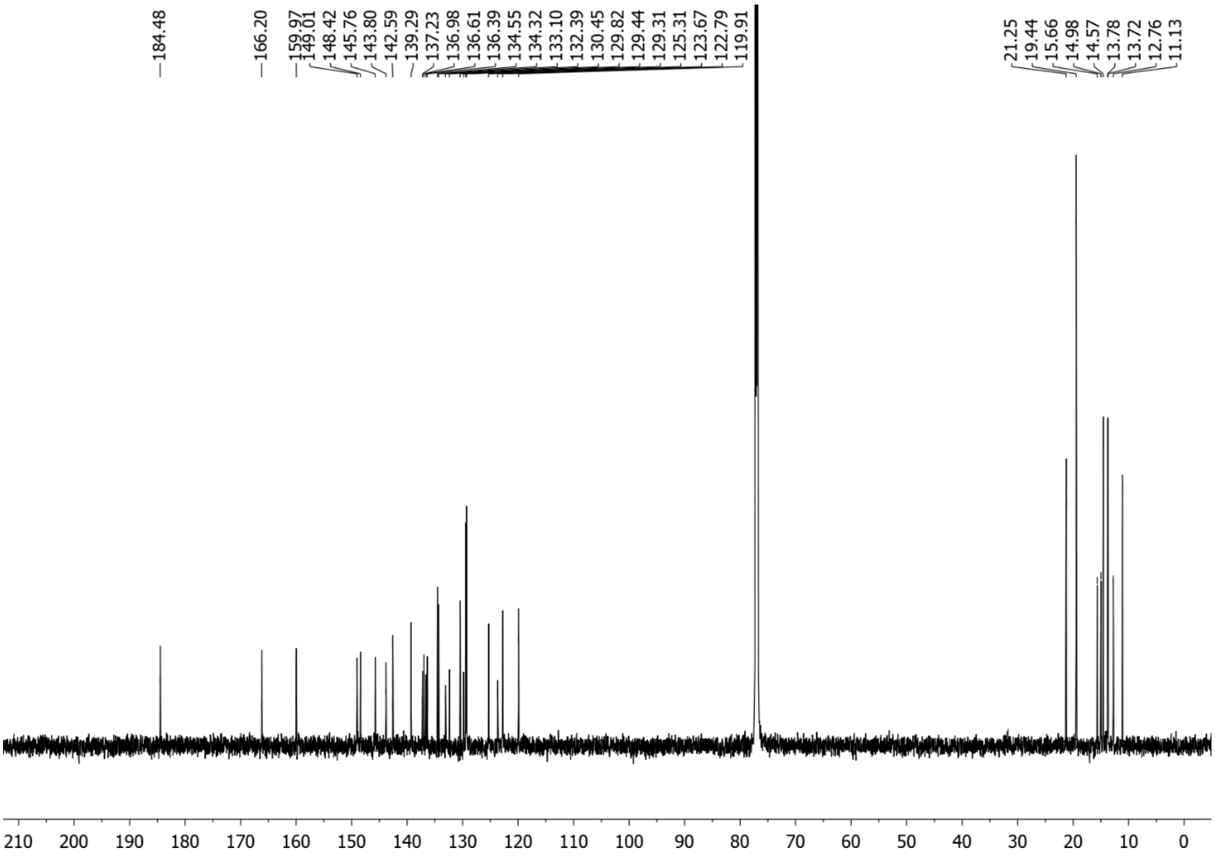

^{1}H NMR (700 MHz, $CDCl_3$) and ^{13}C NMR (176 MHz, $CDCl_3$) spectra of **2c**

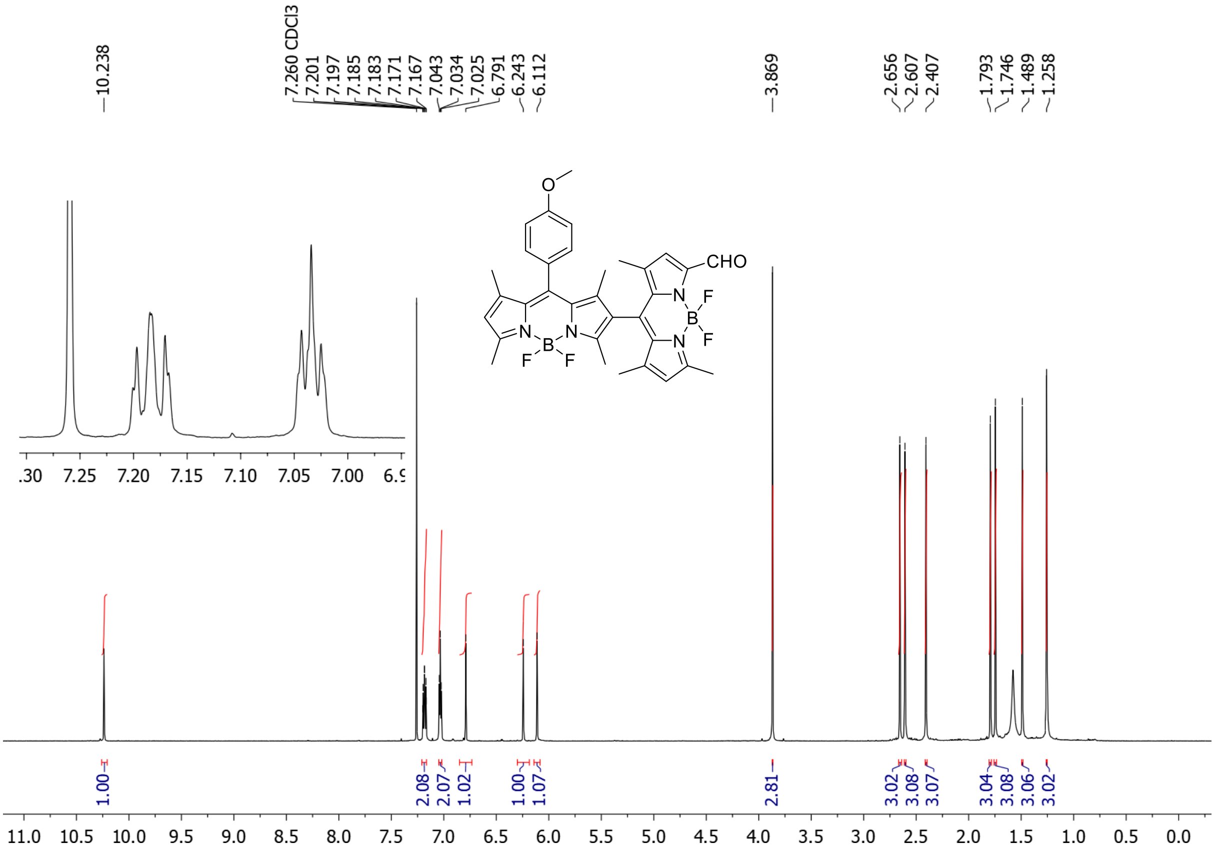

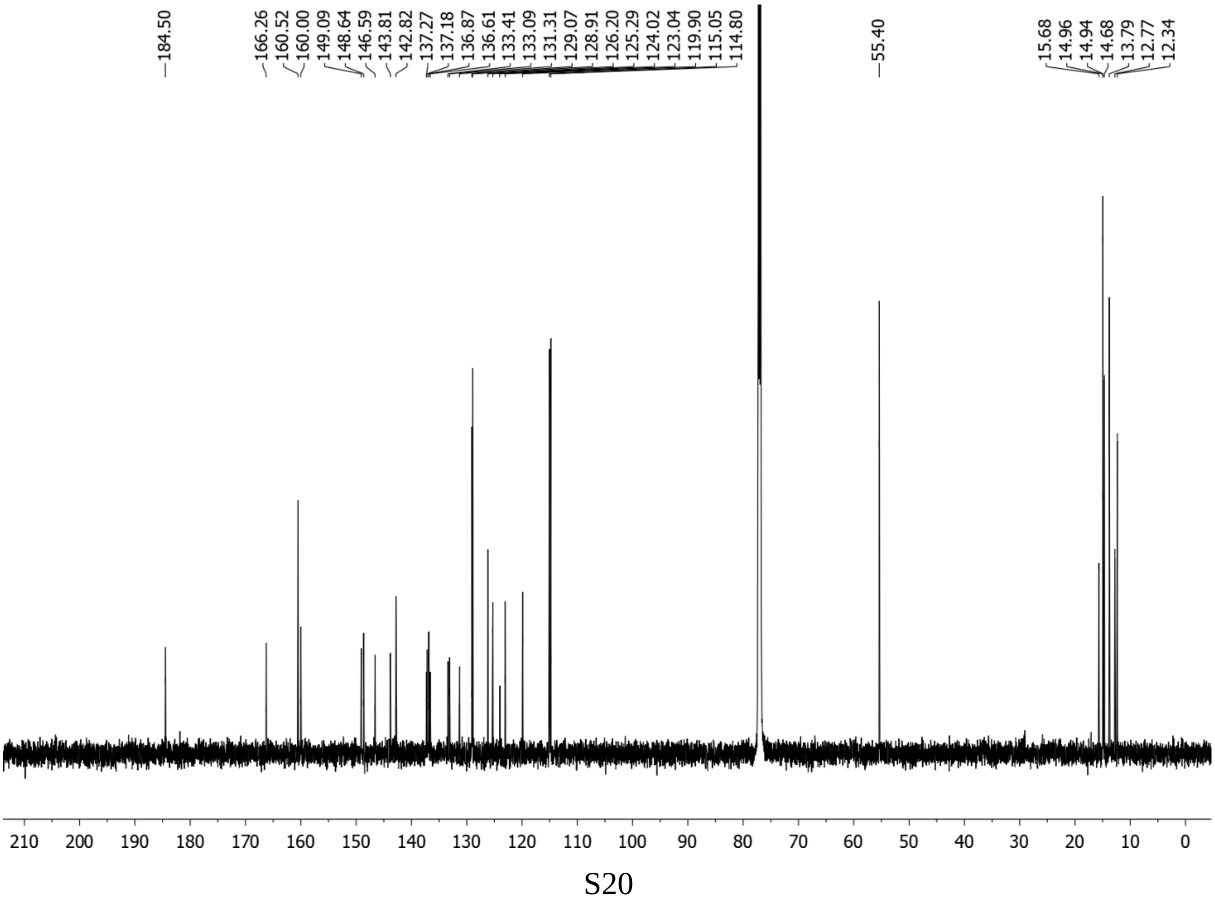

^{1}H NMR (700 MHz, $CDCl_3$) and ^{13}C NMR (176 MHz, $CDCl_3$) spectra of **2d**

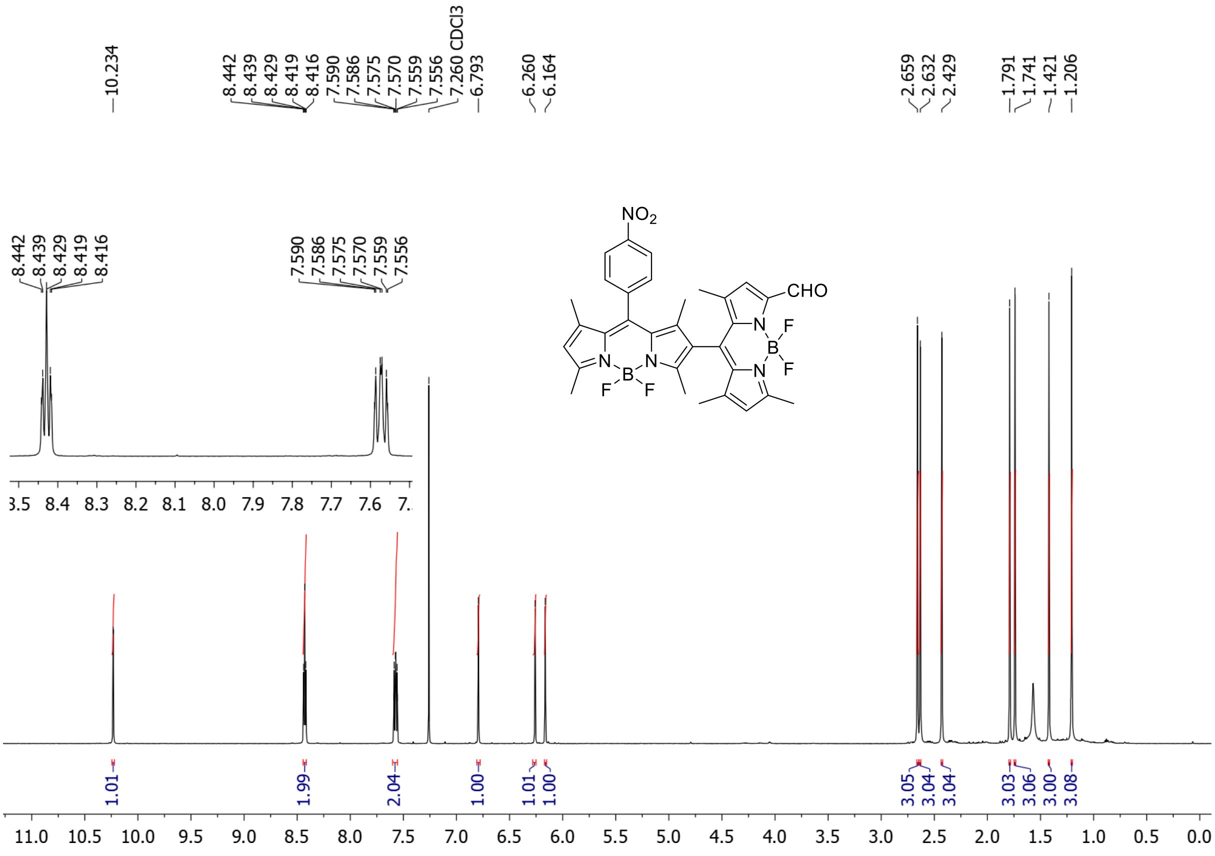

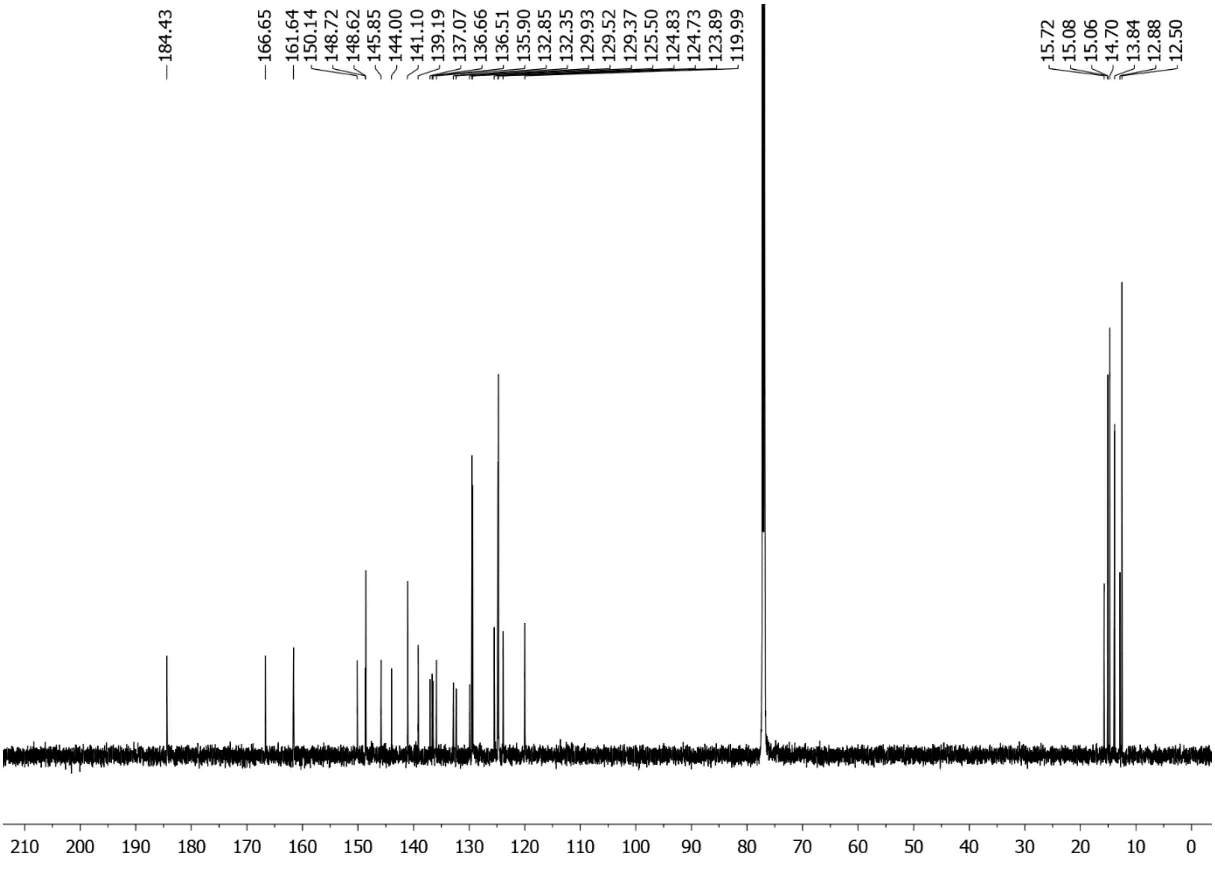

^{1}H NMR (300 MHz, $CDCl_3$) and ^{13}C NMR (75 MHz, $CDCl_3$) spectra of **3a**

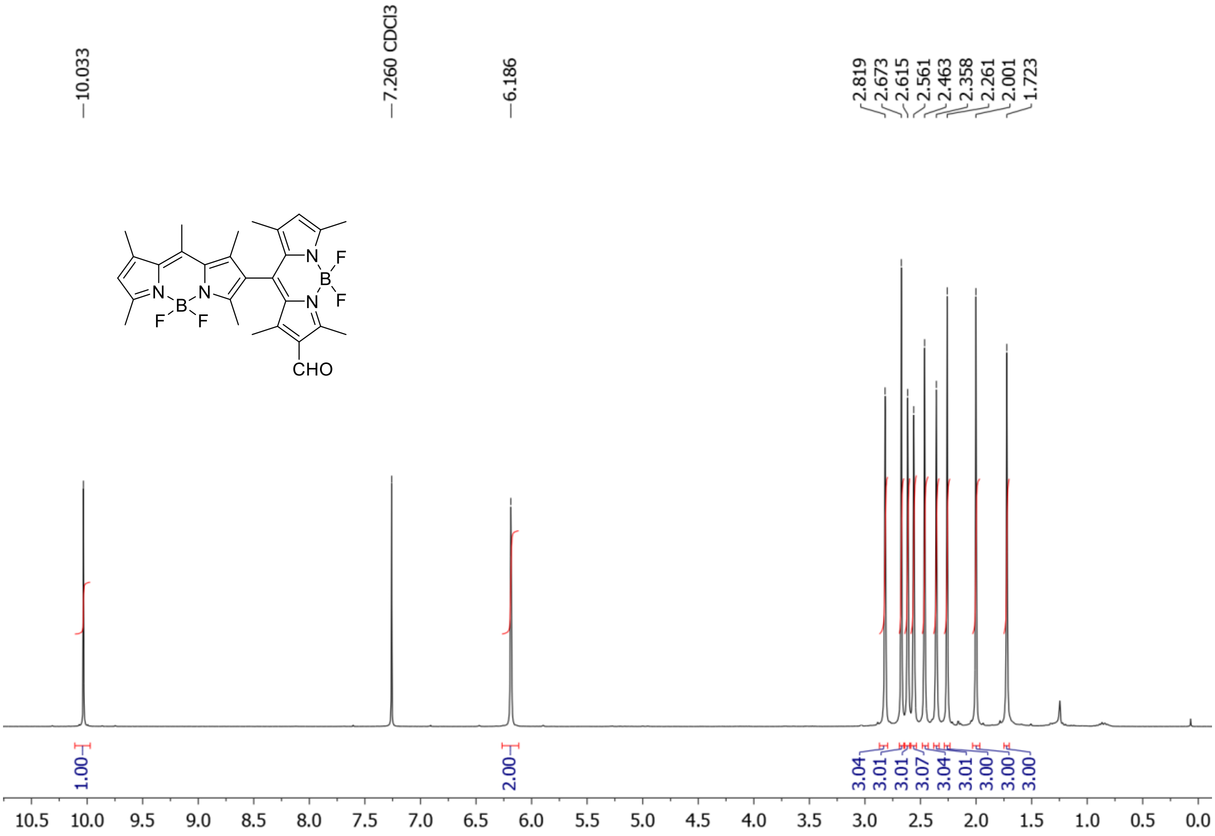

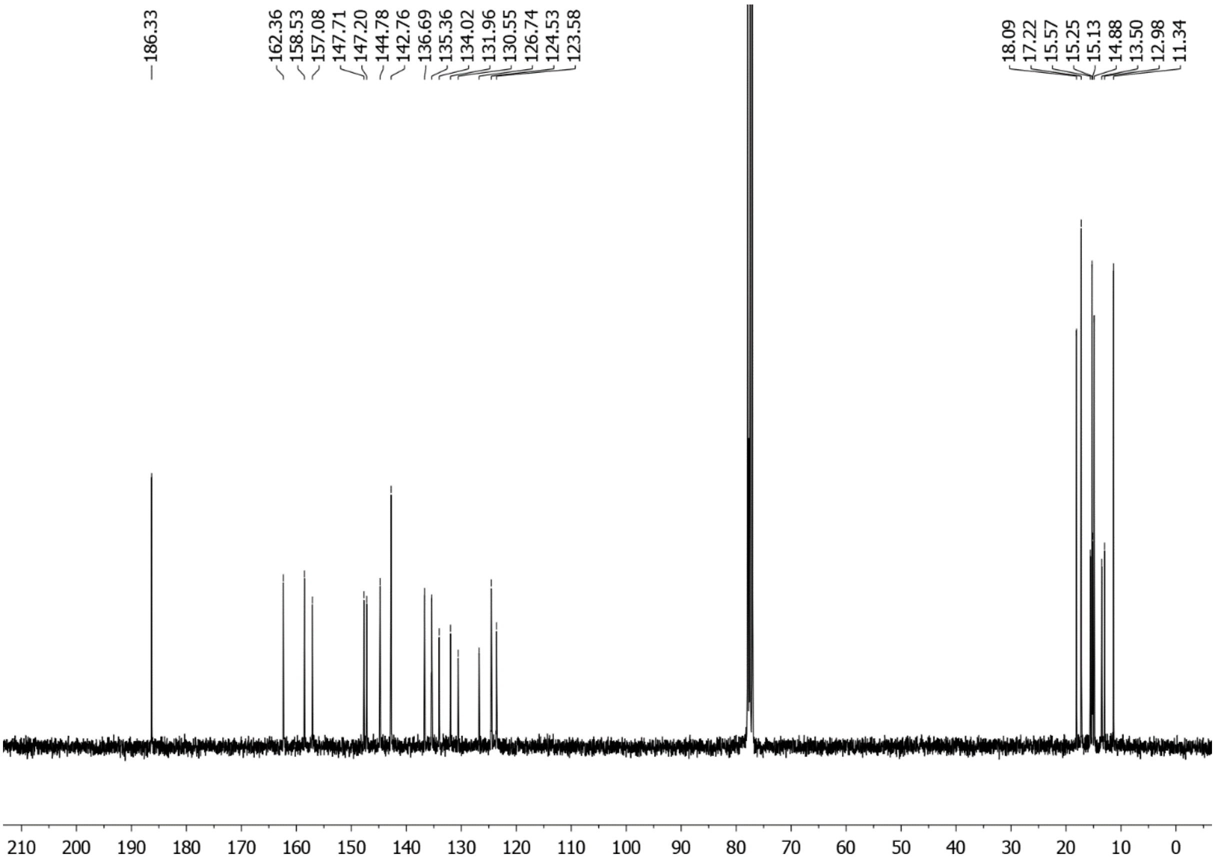

^{1}H NMR (700 MHz, $CDCl_3$) and ^{13}C NMR (176 MHz, $CDCl_3$) spectra of **3b**

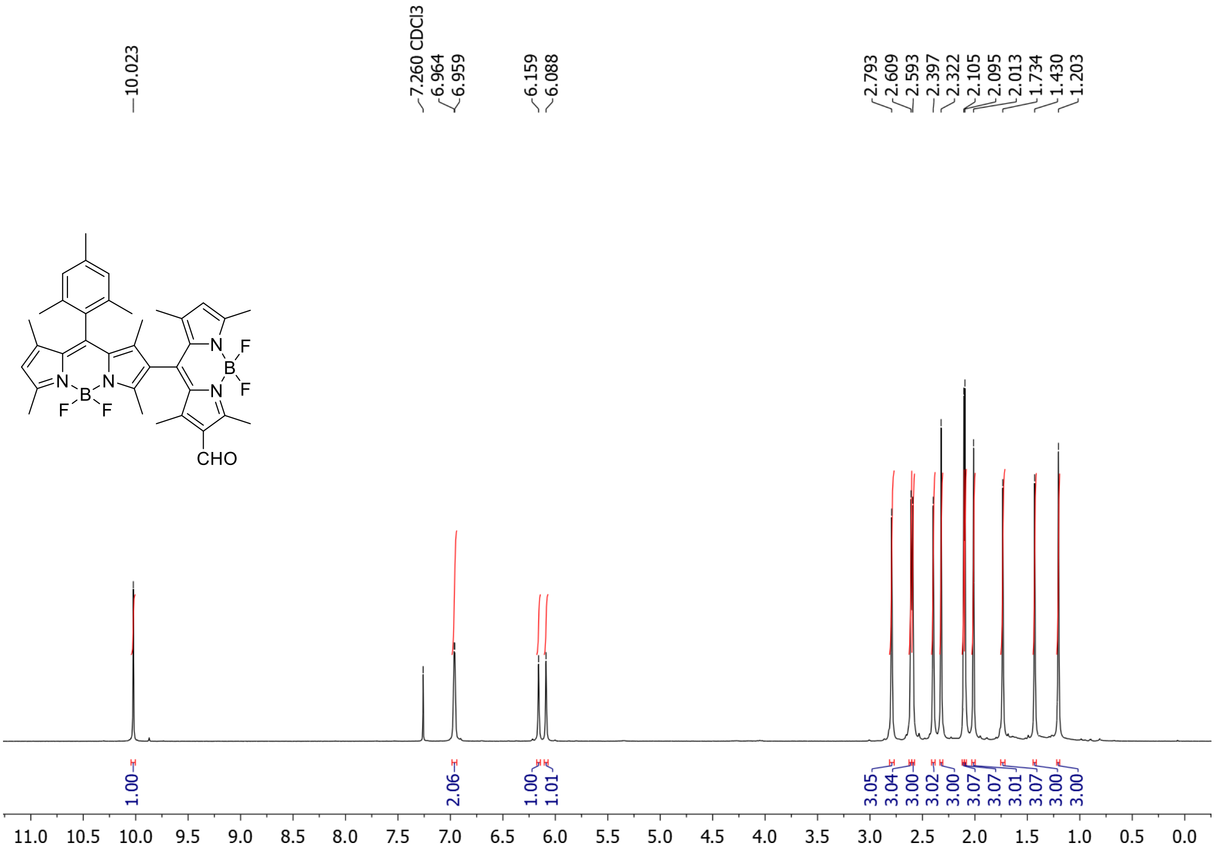

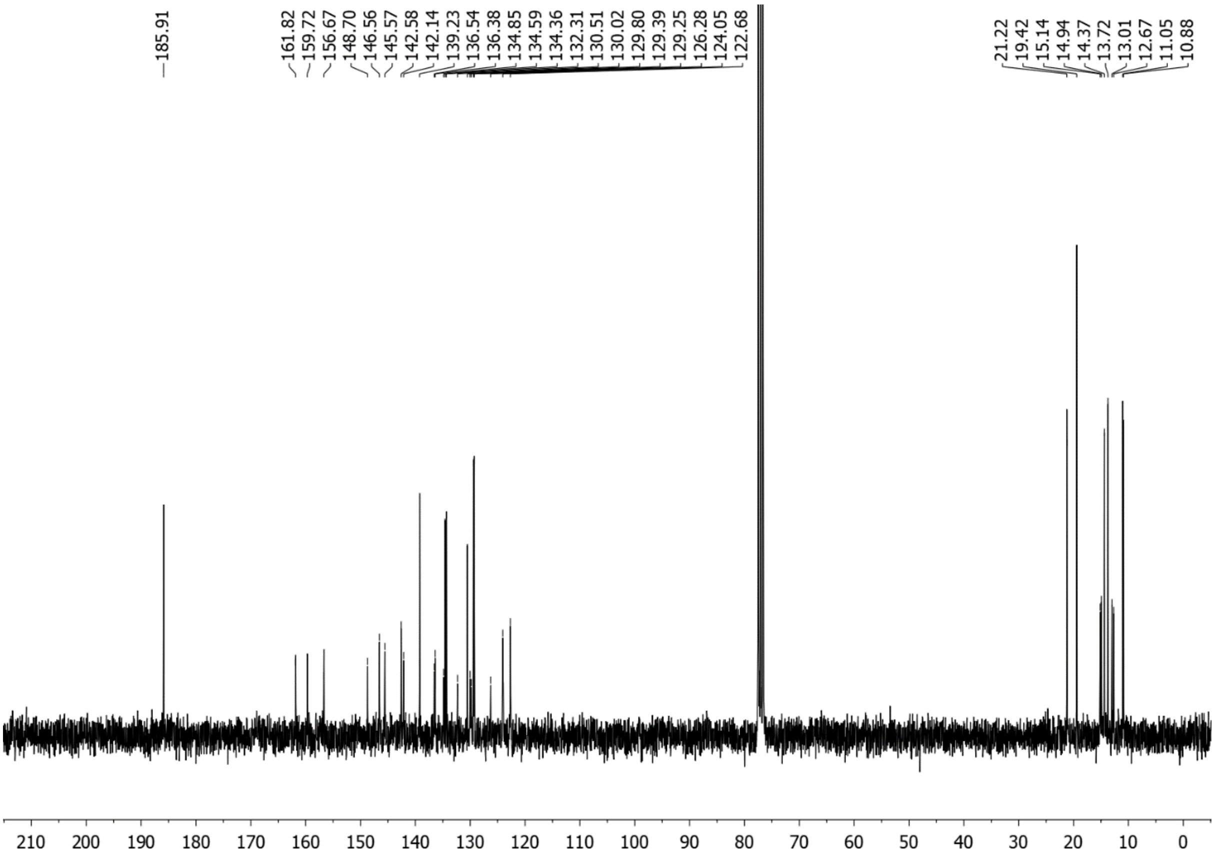

^{1}H NMR (700 MHz, $CDCl_3$) and ^{13}C NMR (176 MHz, $CDCl_3$) spectra of **3c**

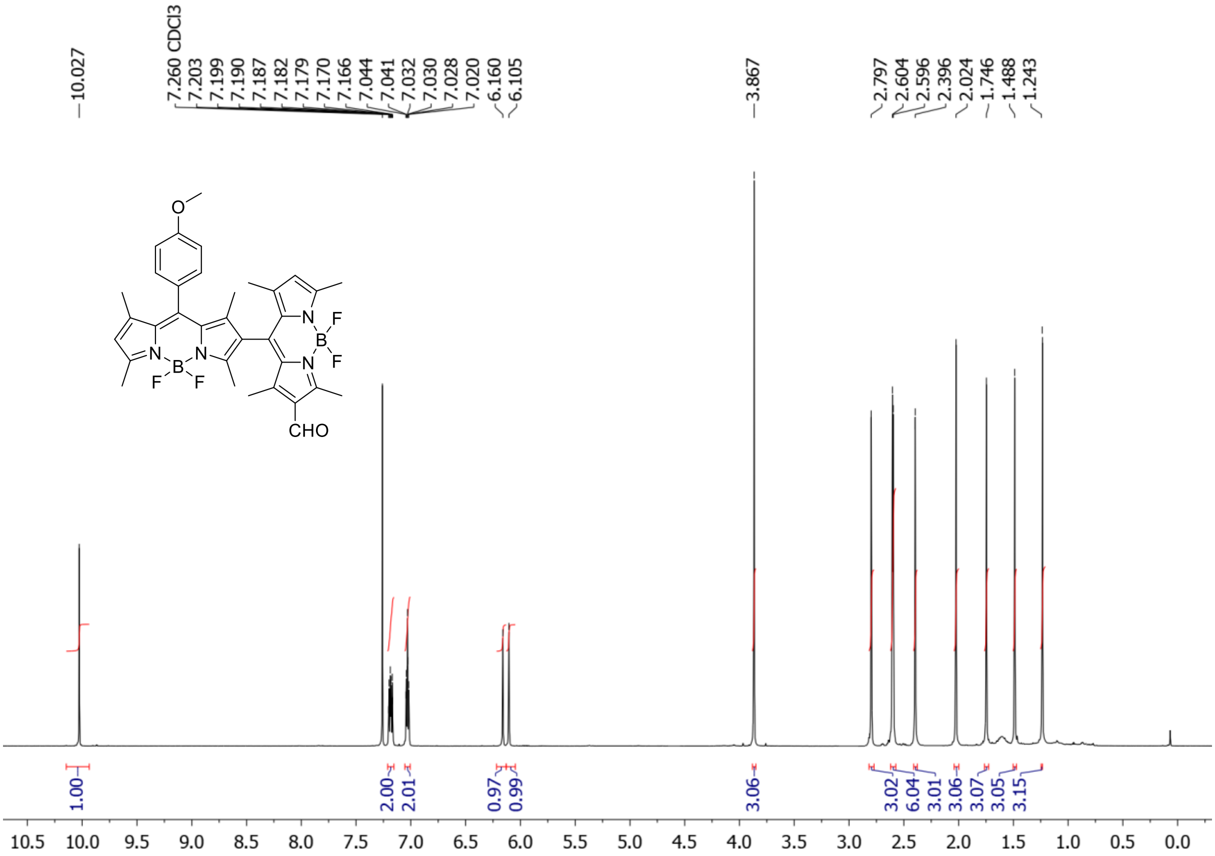

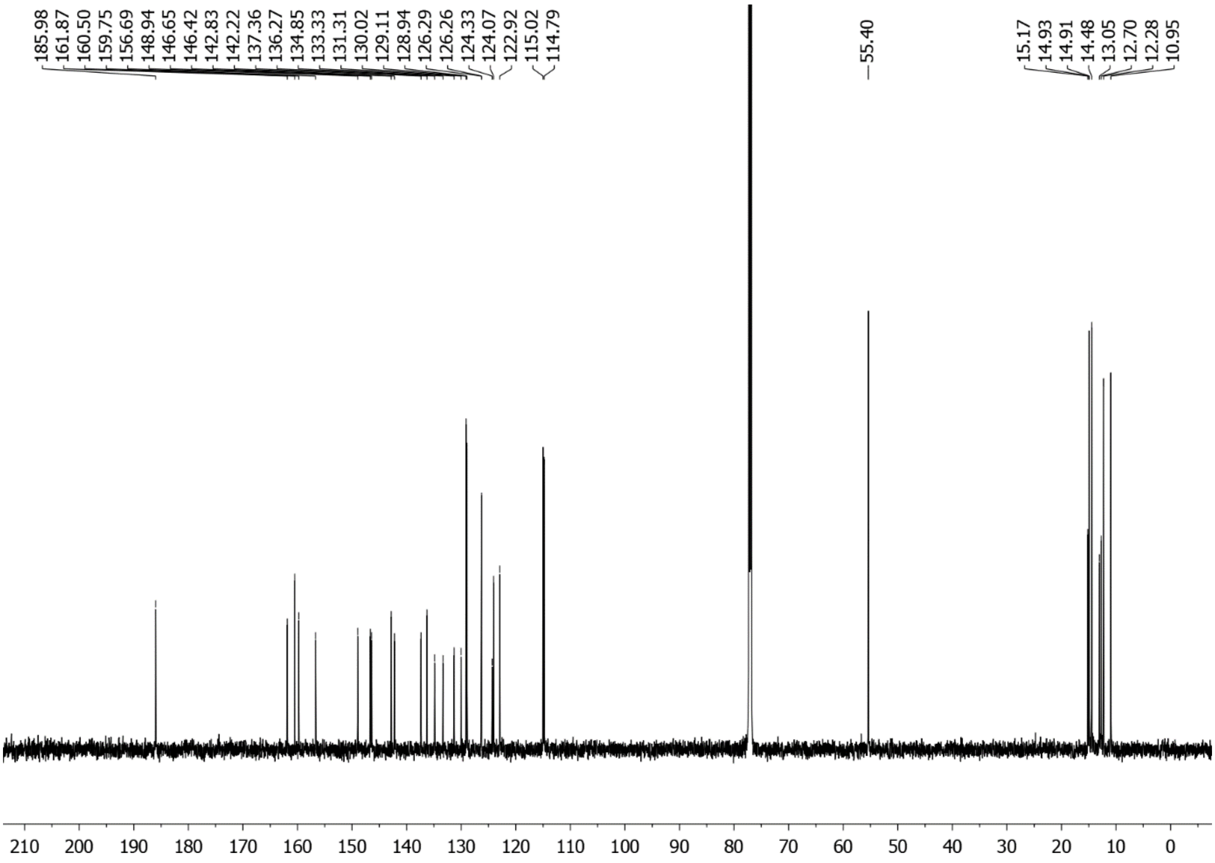

^{1}H NMR (700 MHz, $CDCl_3$) and ^{13}C NMR (176 MHz, $CDCl_3$) spectra of **3d**

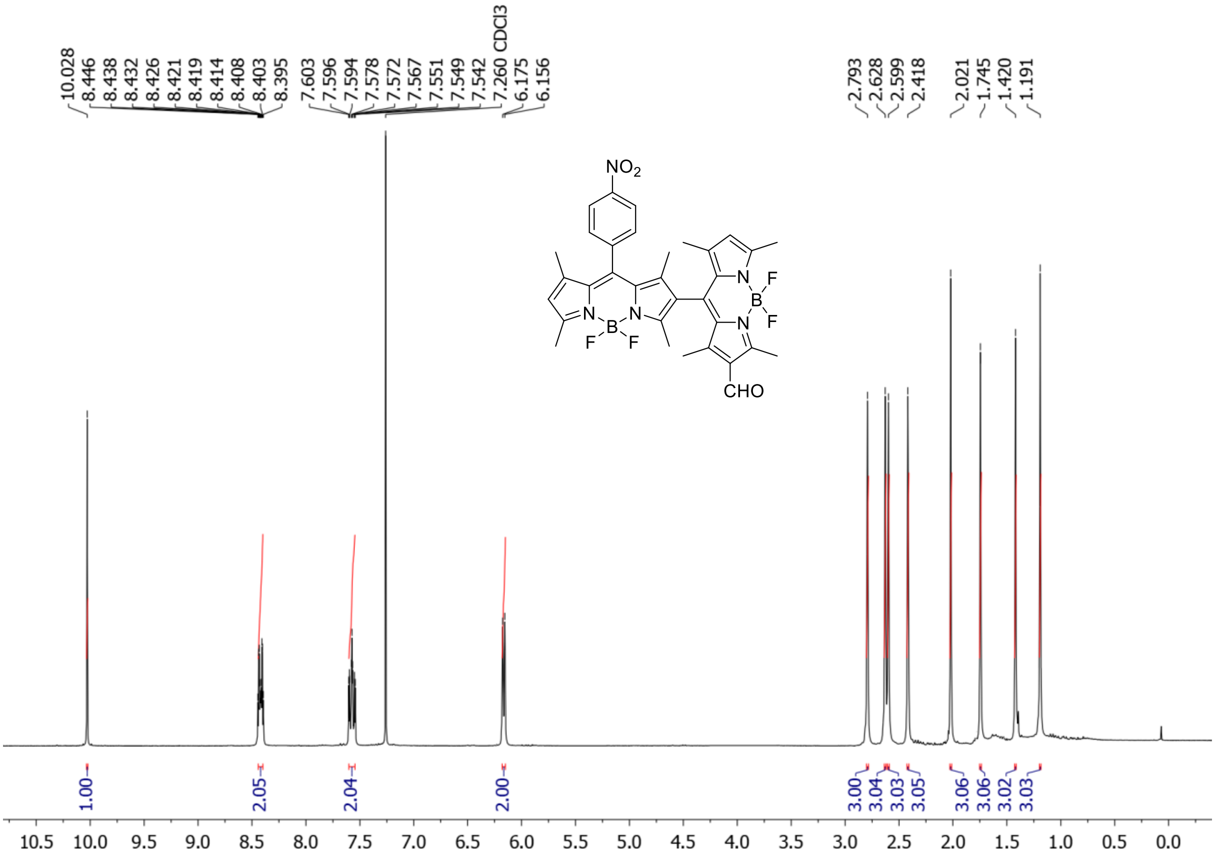

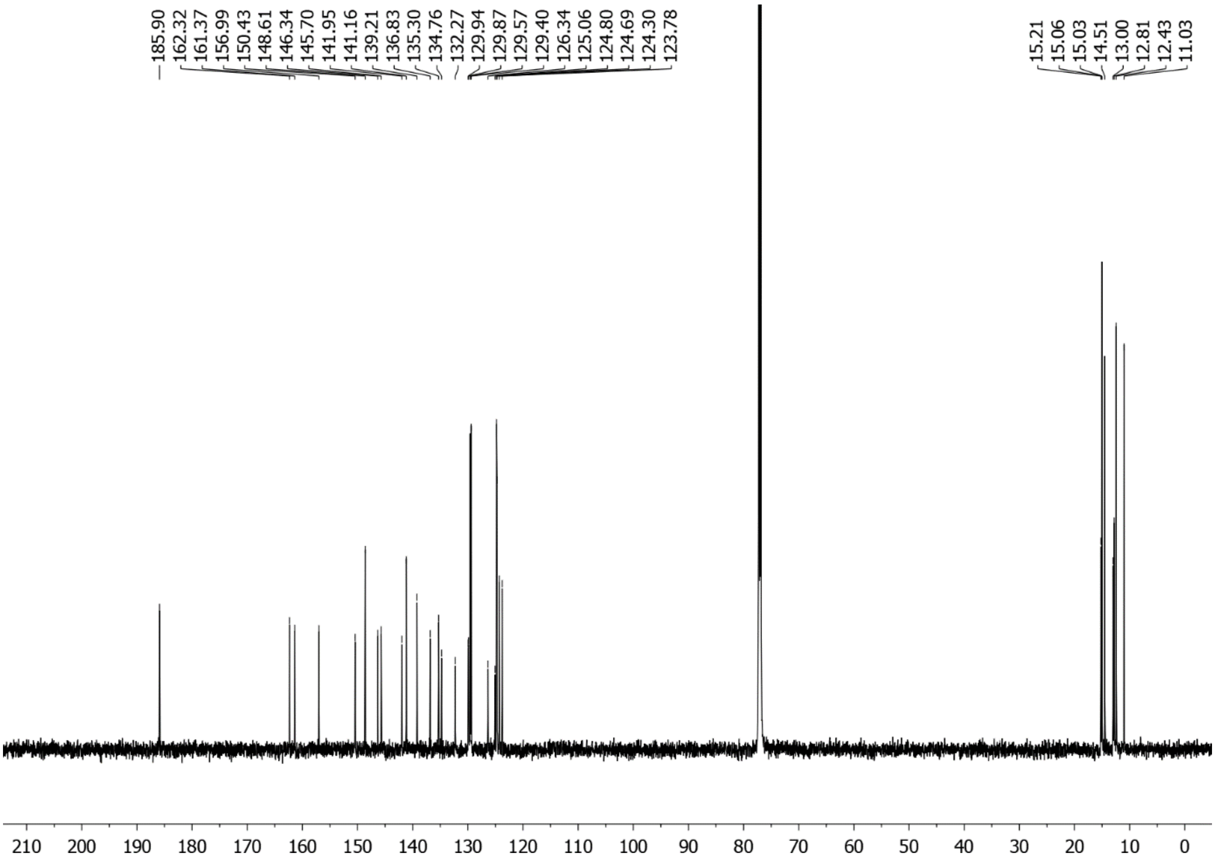

^{1}H NMR (300 MHz, $CDCl_3$) and ^{13}C NMR (75 MHz, $CDCl_3$) spectra of **4**

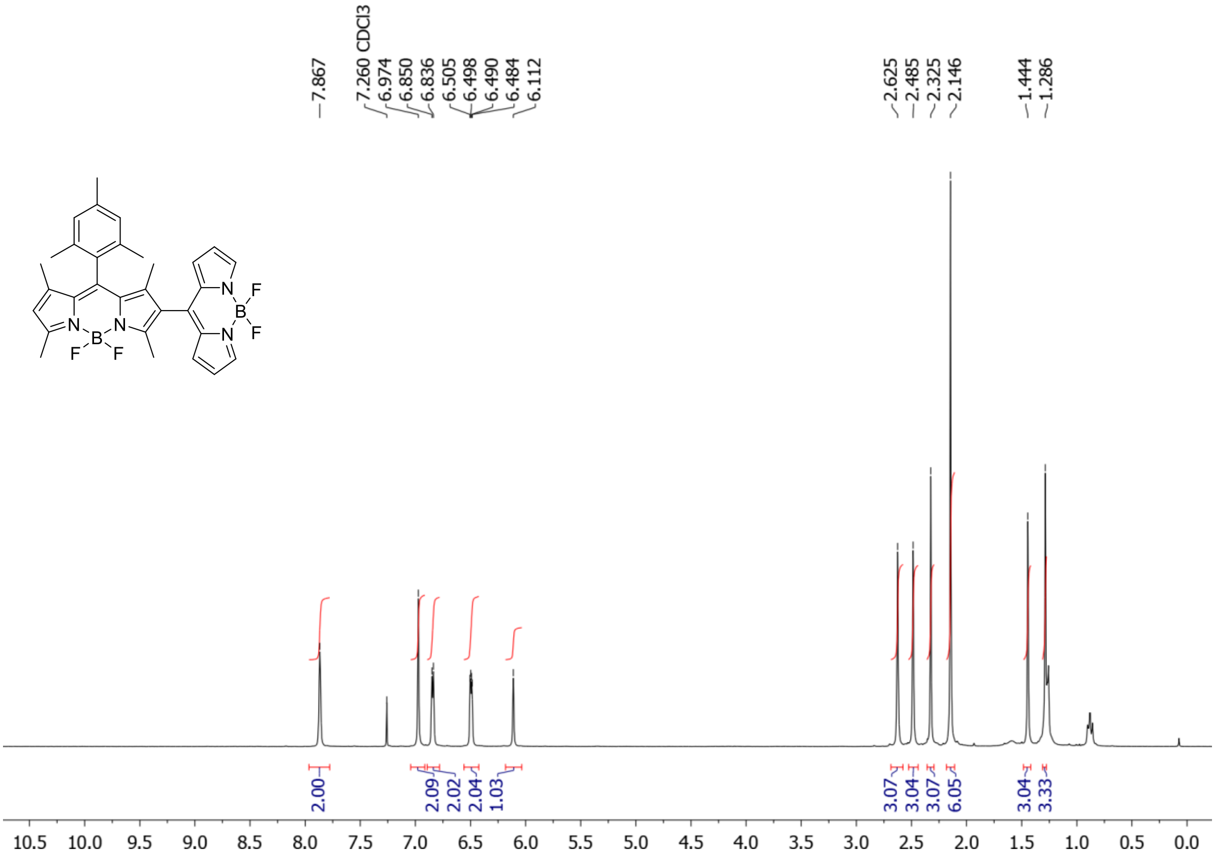

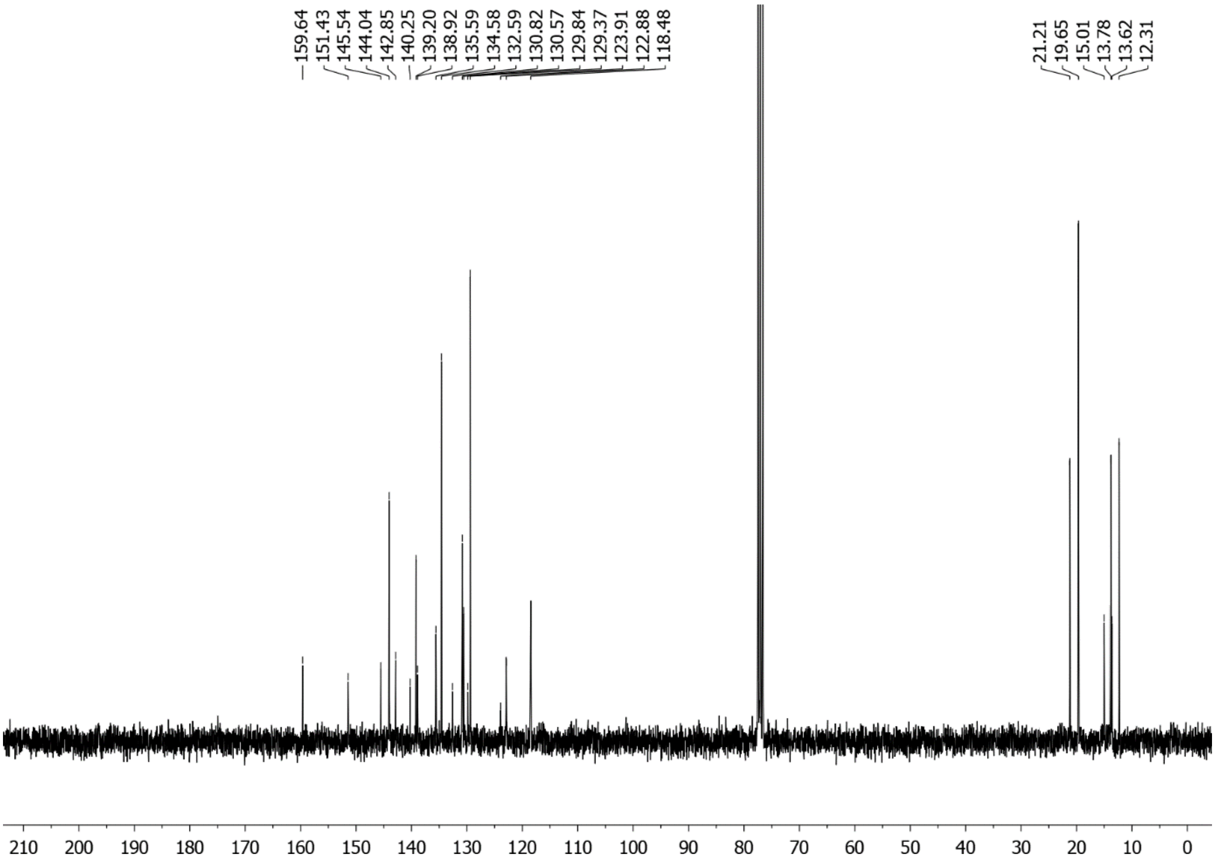

^{1}H NMR (700 MHz, $CDCl_3$) and ^{13}C NMR (176 MHz, $CDCl_3$) spectra of **5a**

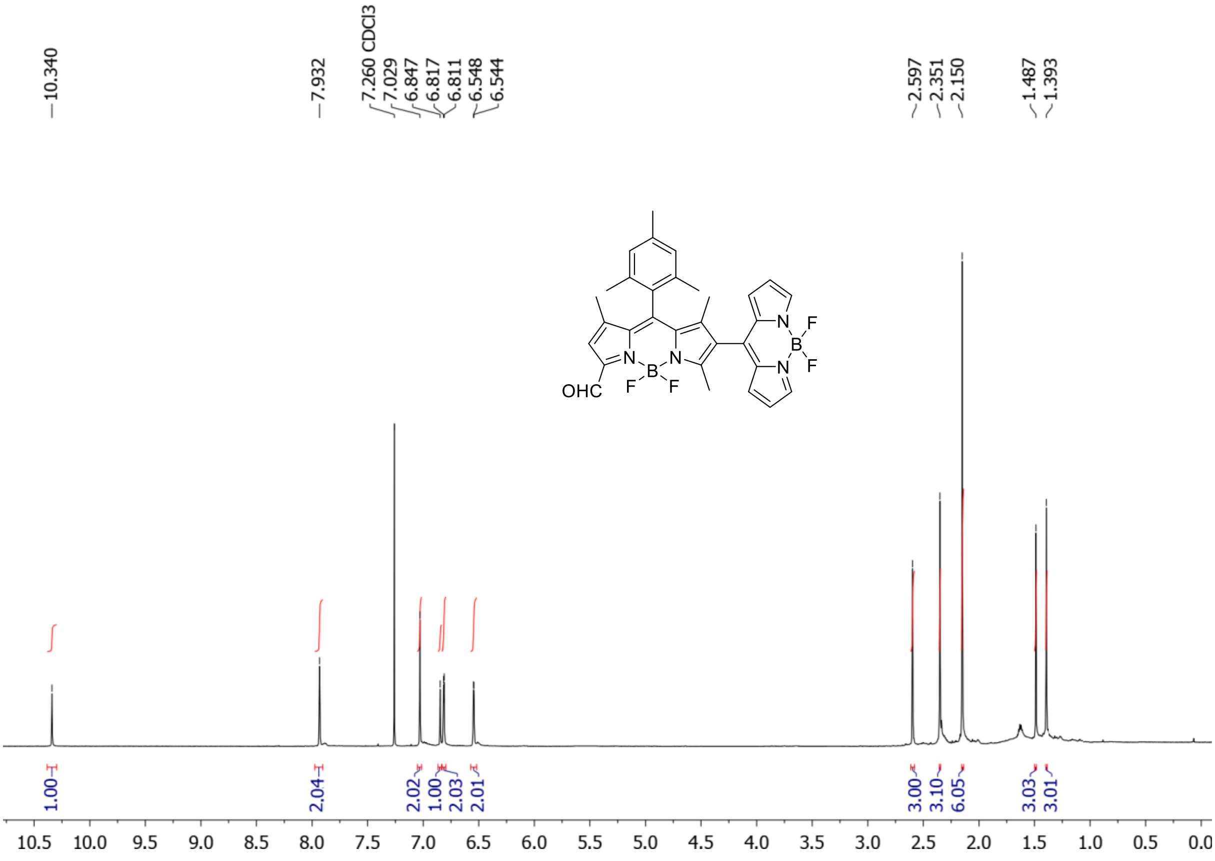

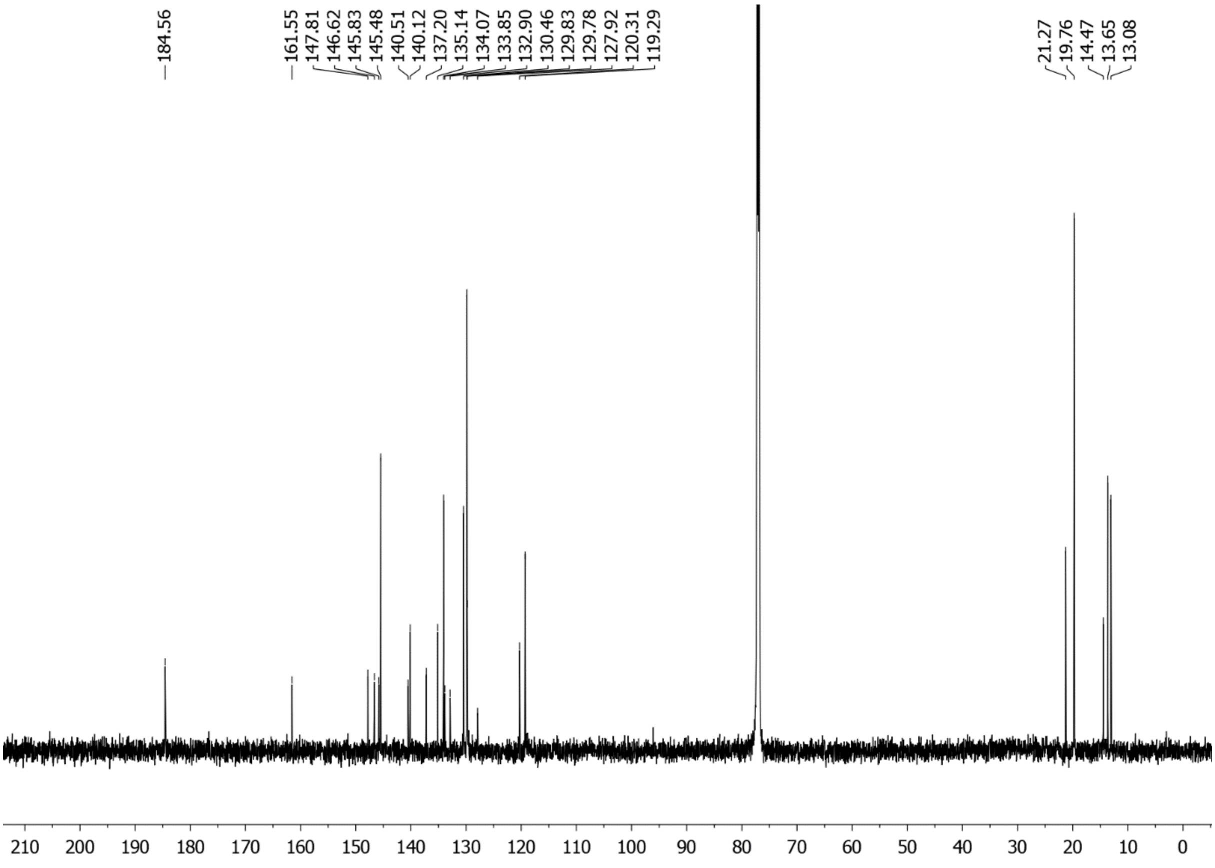

^{1}H NMR (700 MHz, $CDCl_3$) and ^{13}C NMR (176 MHz, $CDCl_3$) spectra of **5b**

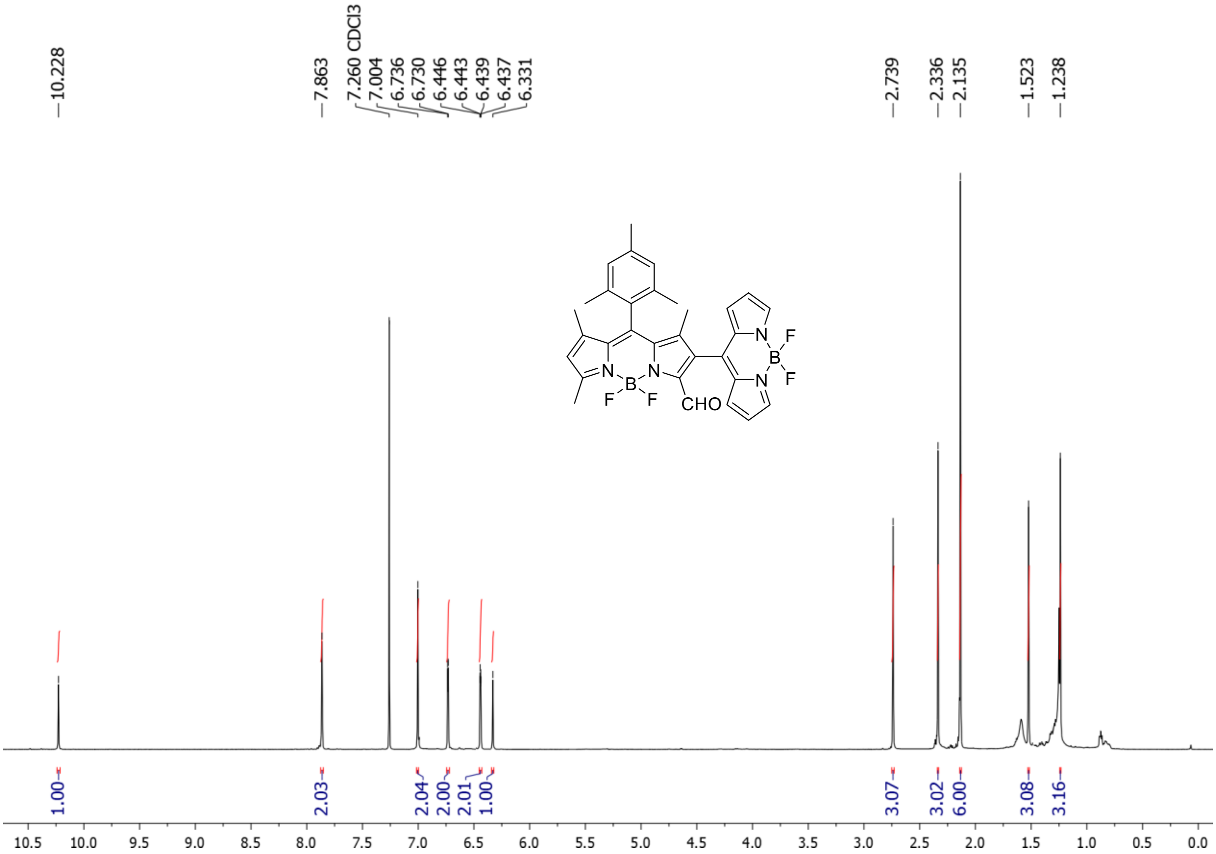

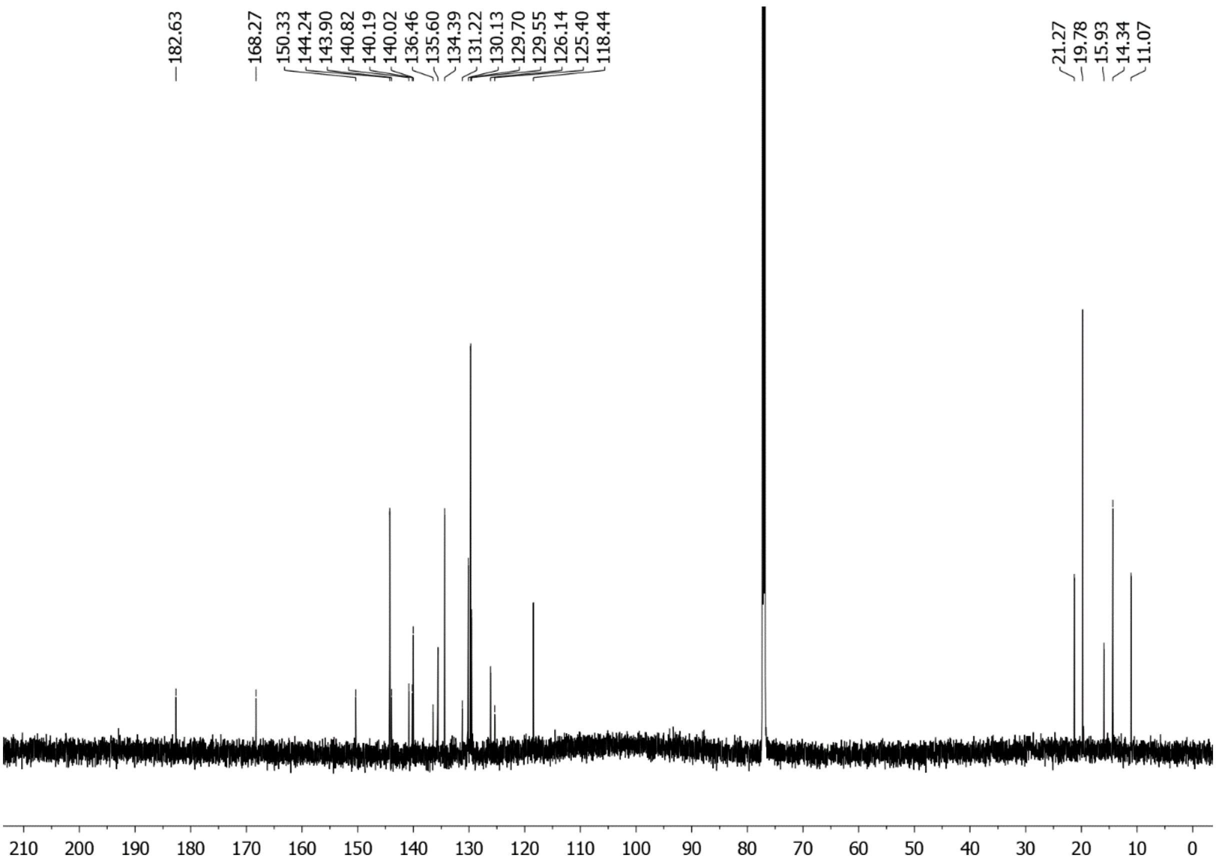

^{1}H NMR (700 MHz, $CDCl_3$) and ^{13}C NMR (176 MHz, $CDCl_3$) spectra of **6a**

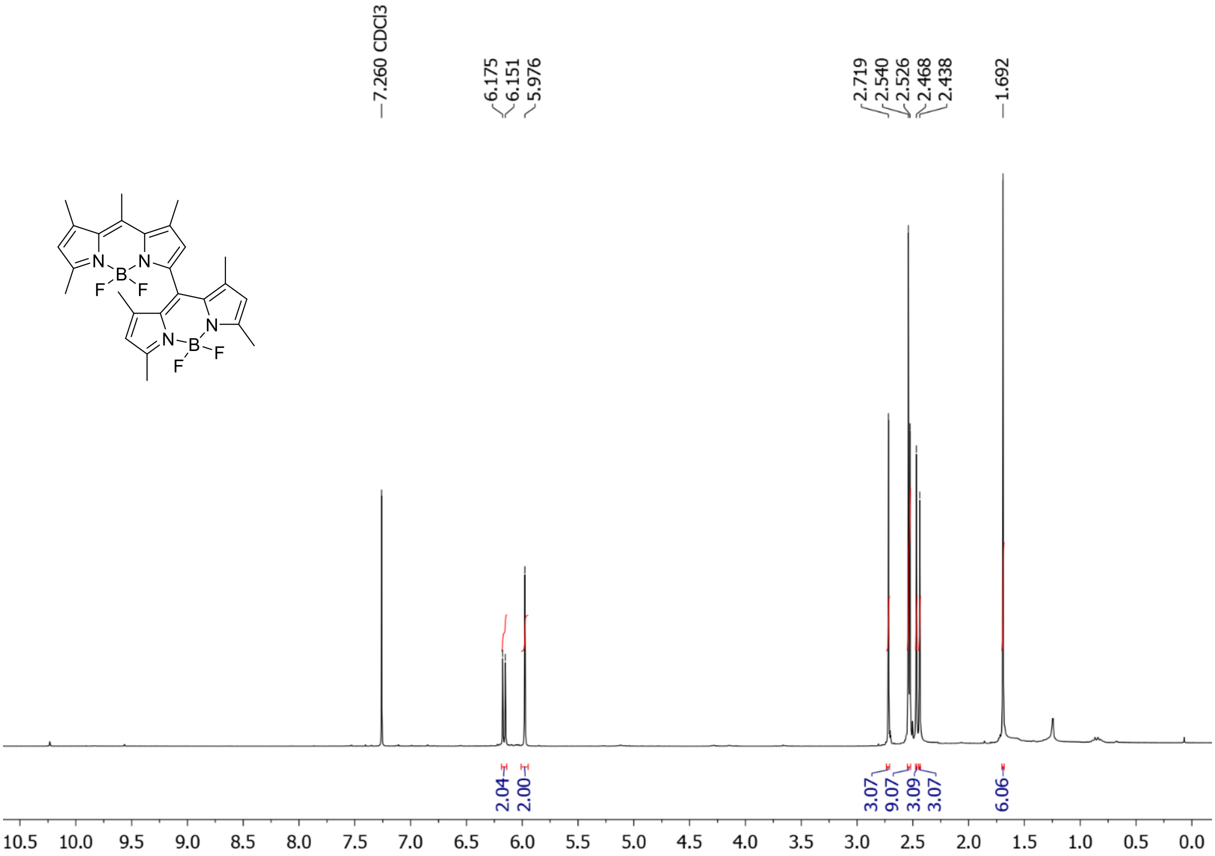

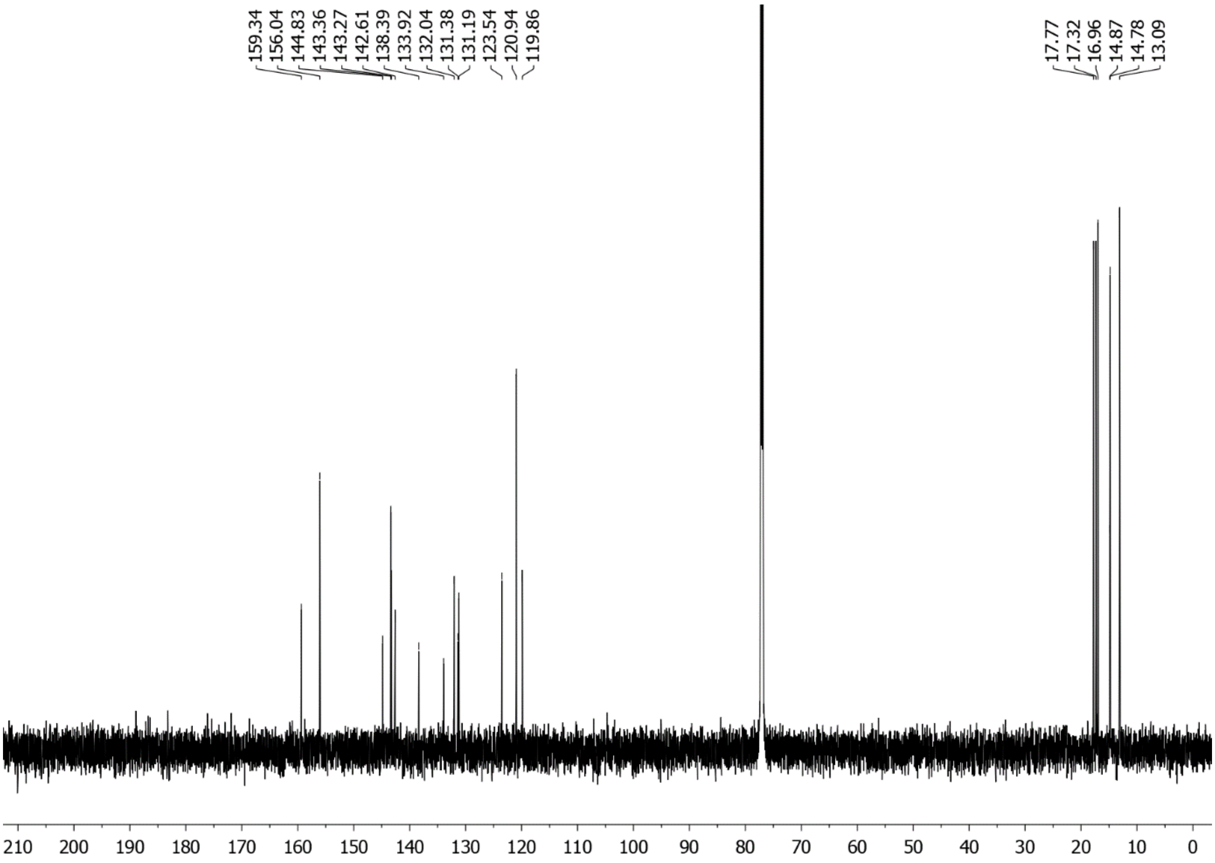

^{1}H NMR (300 MHz, $CDCl_3$) and ^{13}C NMR (75 MHz, $CDCl_3$) spectra of **6b**

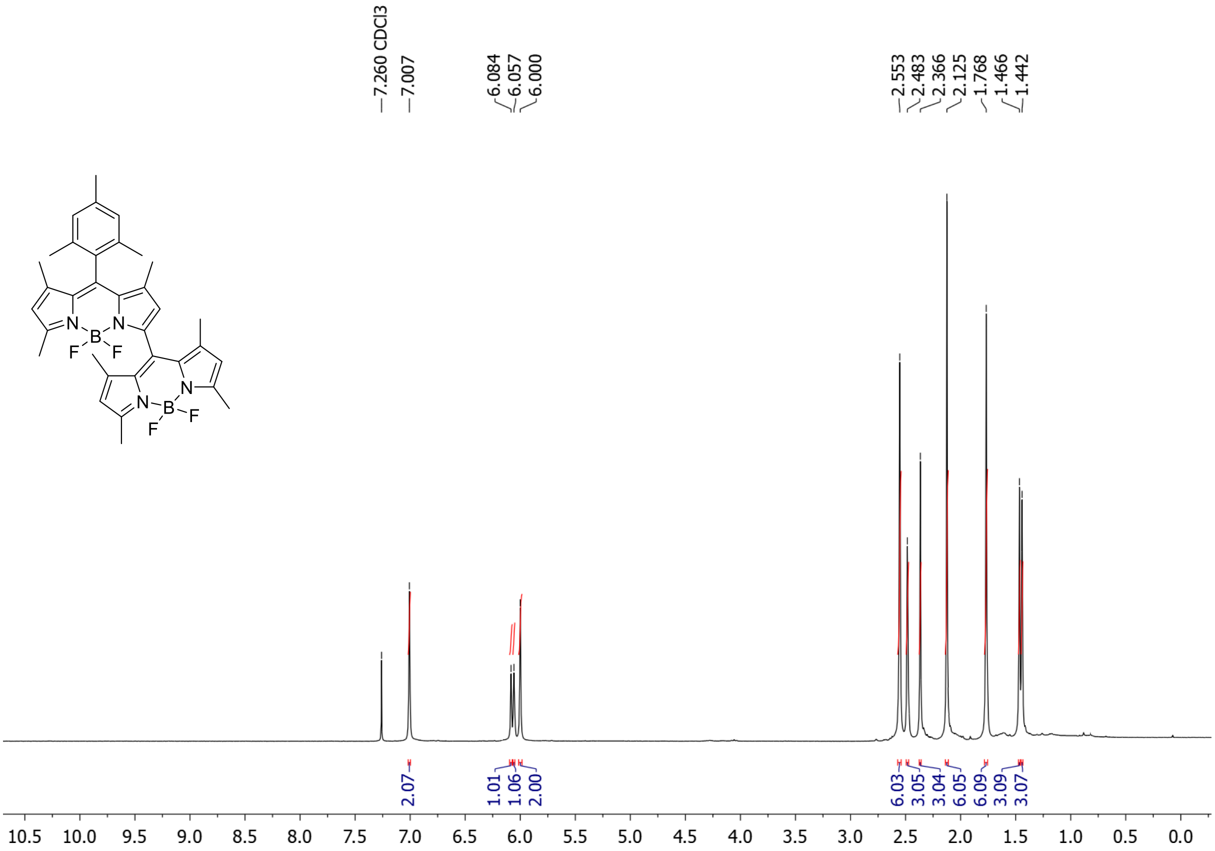

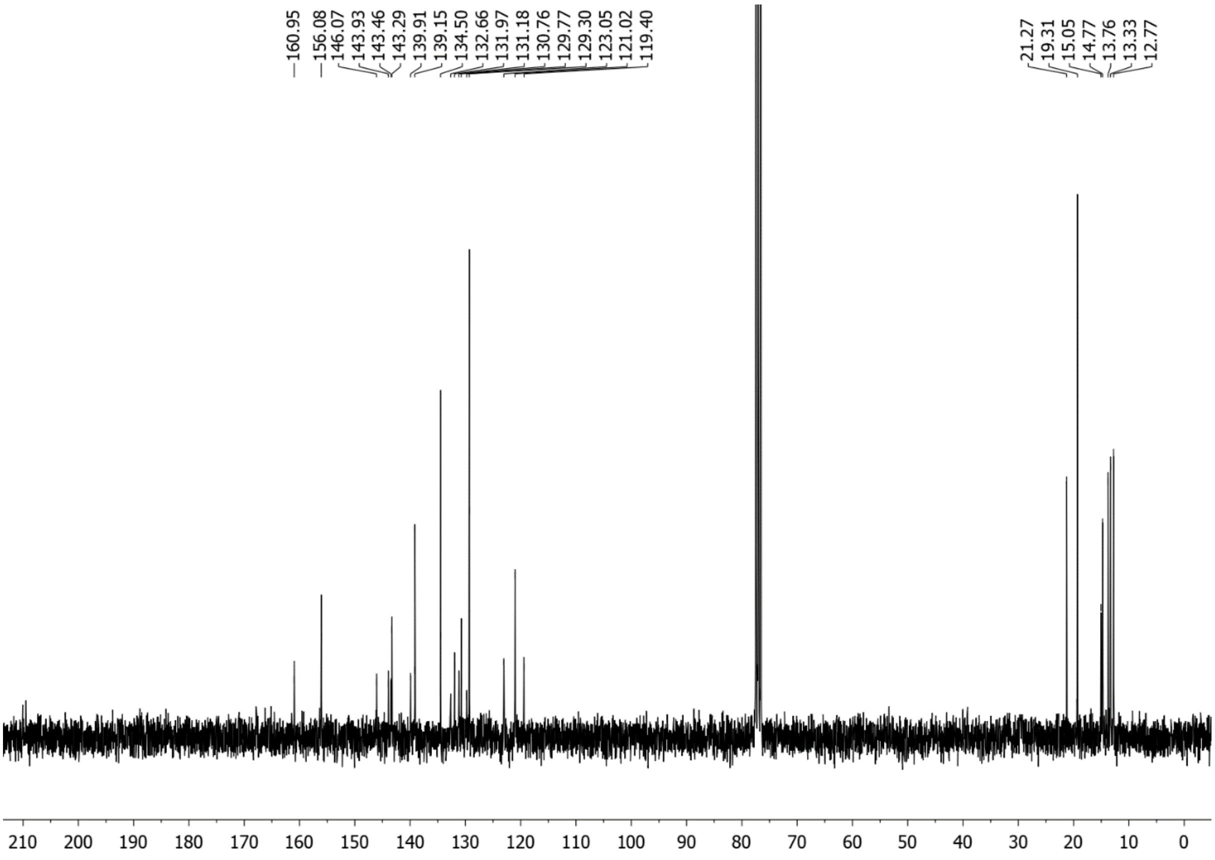

^{1}H NMR (700 MHz, $CDCl_3$) and ^{13}C NMR (176 MHz, $CDCl_3$) spectra of **7a**

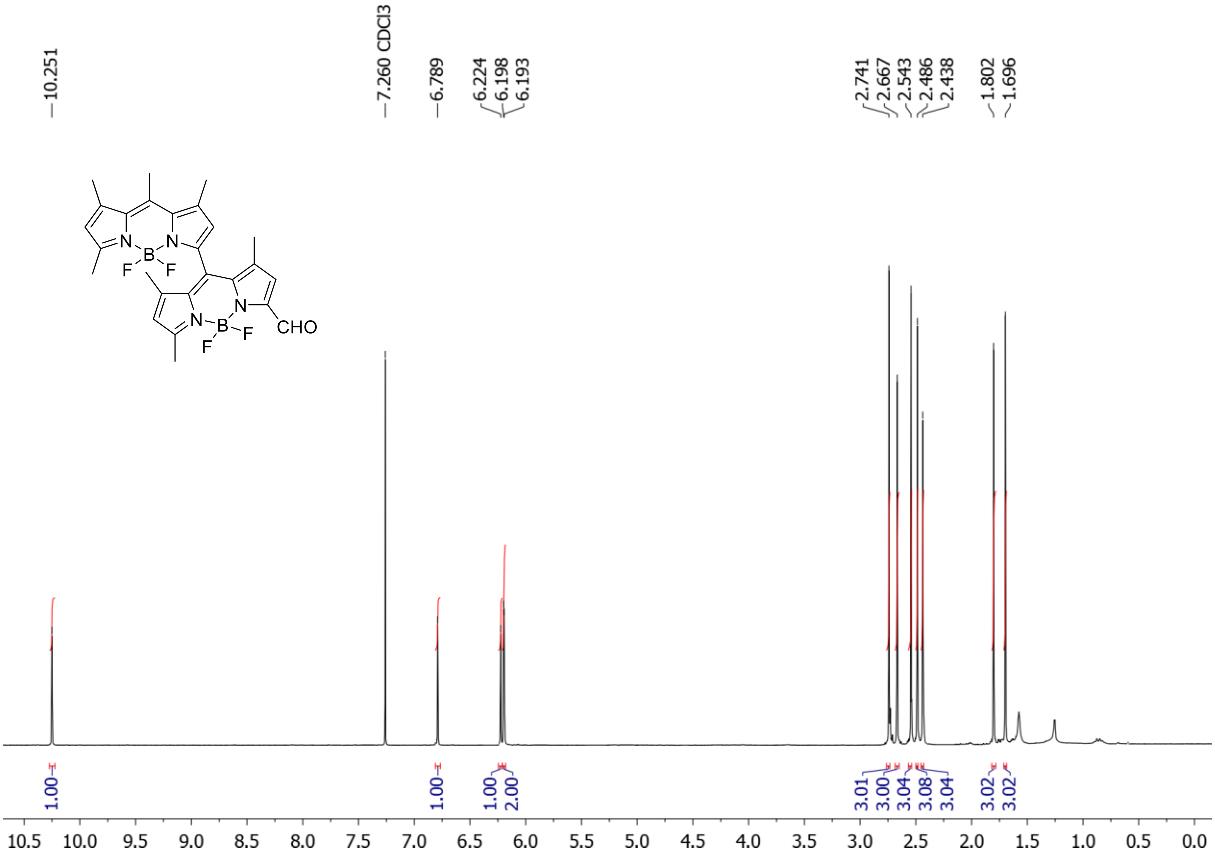

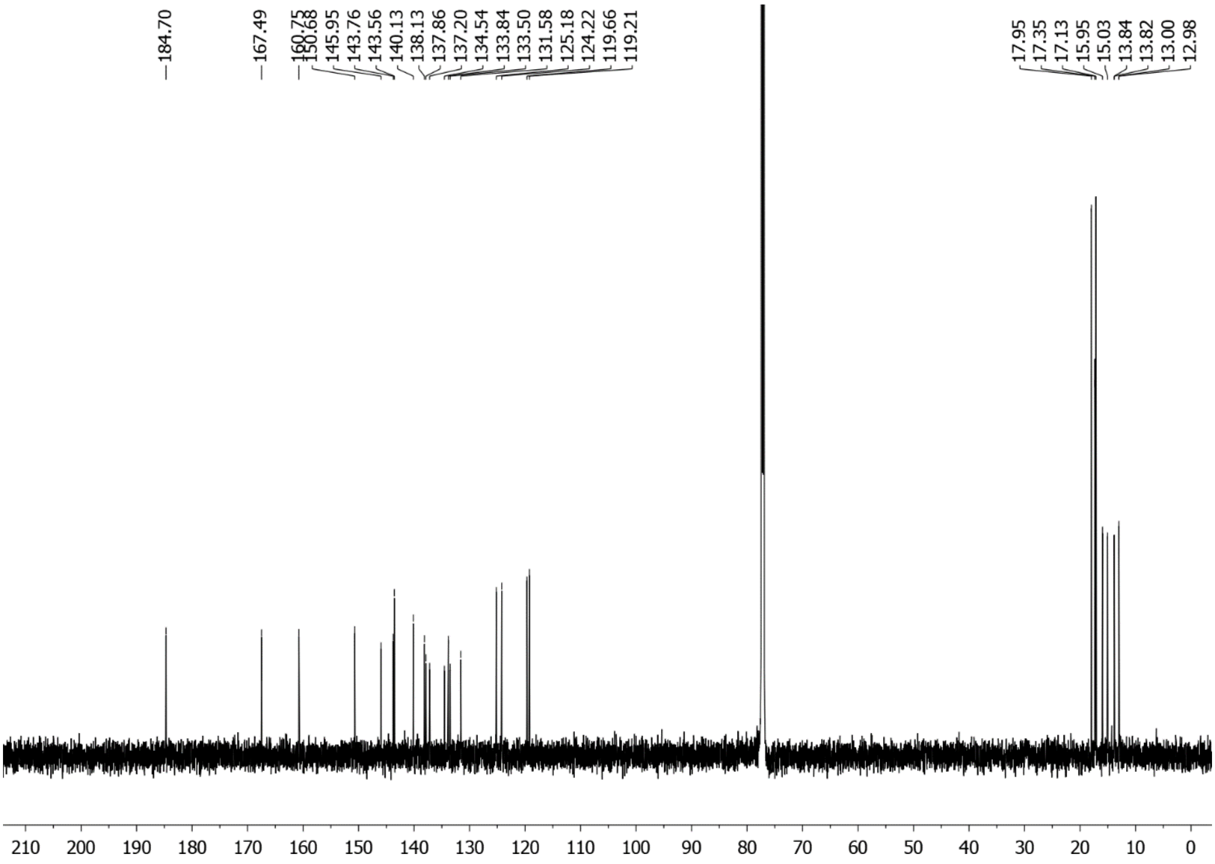

^{1}H NMR (700 MHz, $CDCl_3$) and ^{13}C NMR (176 MHz, $CDCl_3$) spectra of **7b**

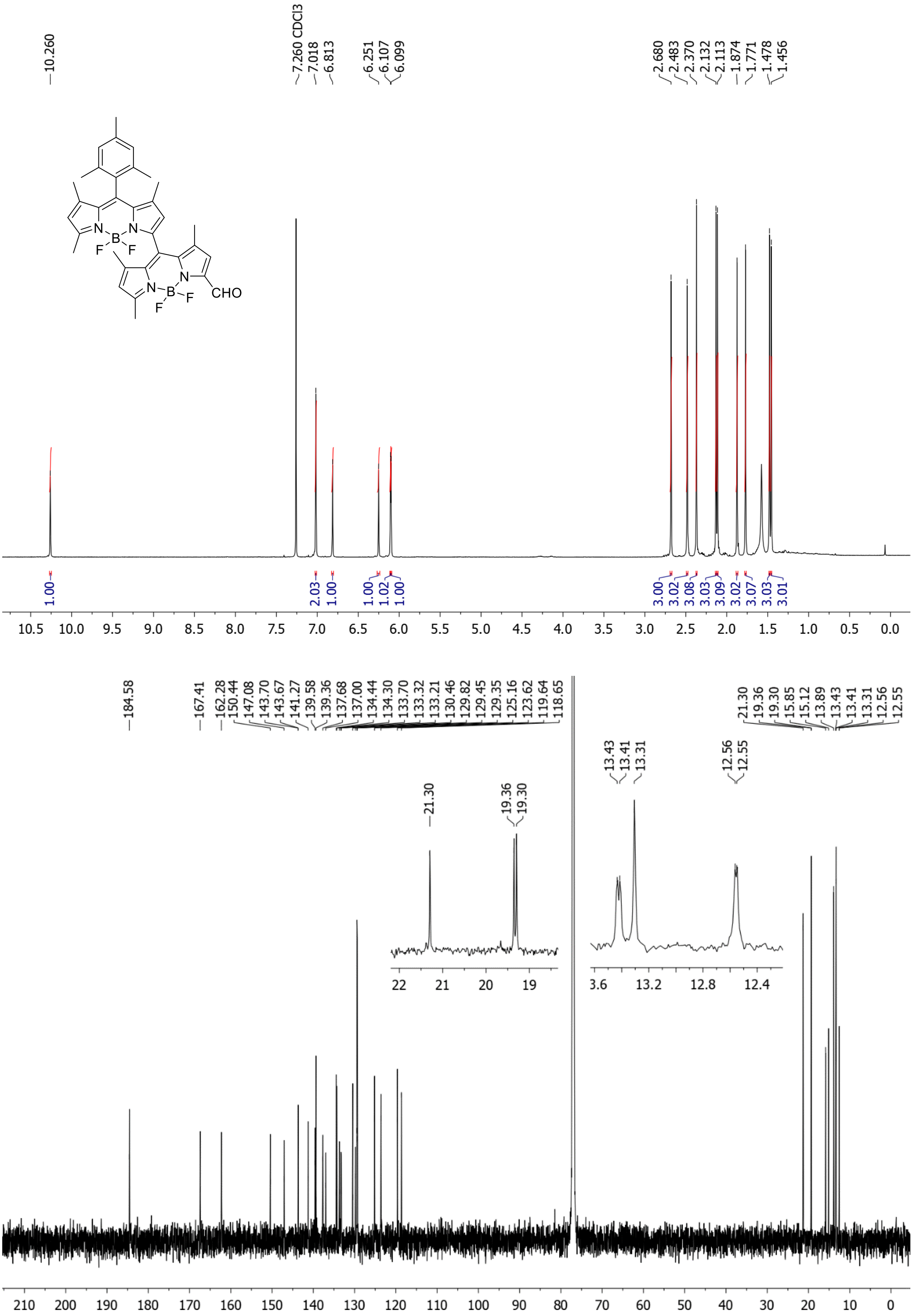

^{1}H NMR (700 MHz, $CDCl_3$) and ^{13}C NMR (176 MHz, $CDCl_3$) spectra of **8a**

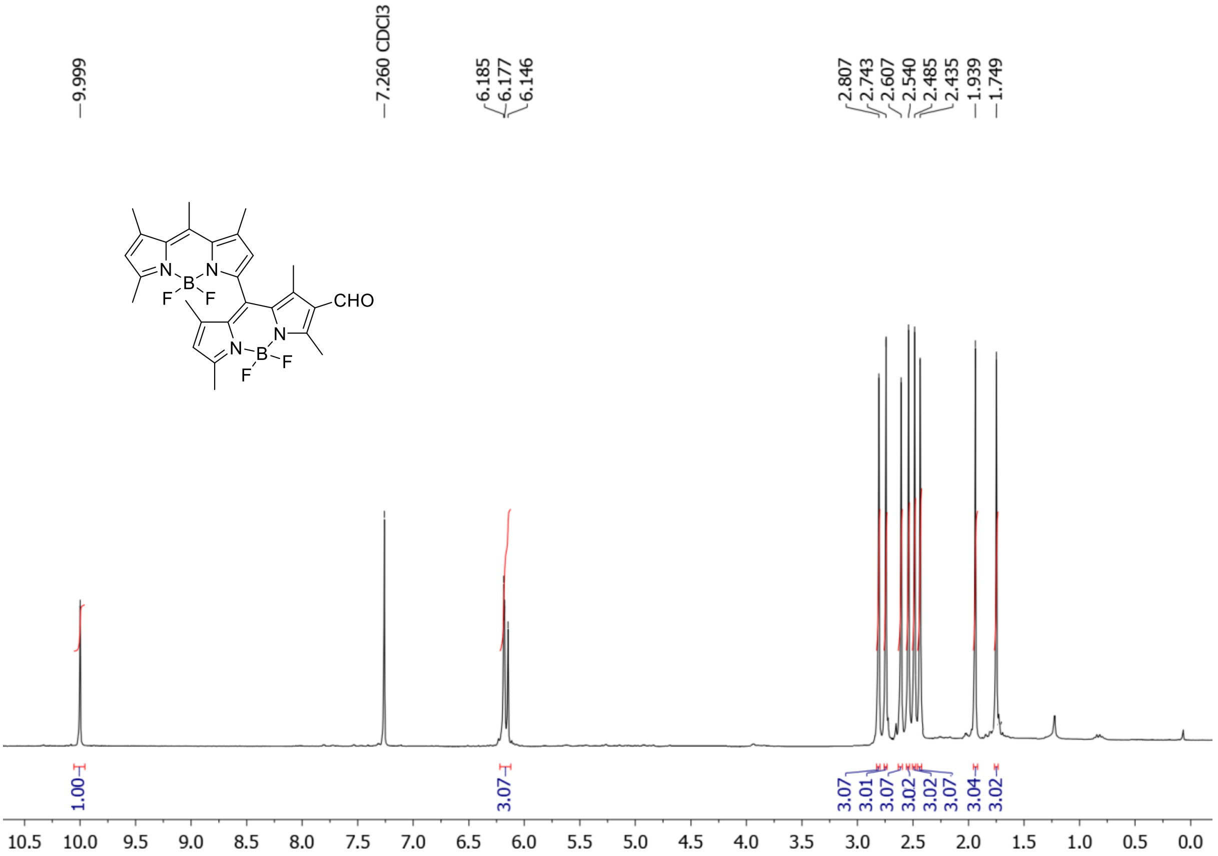

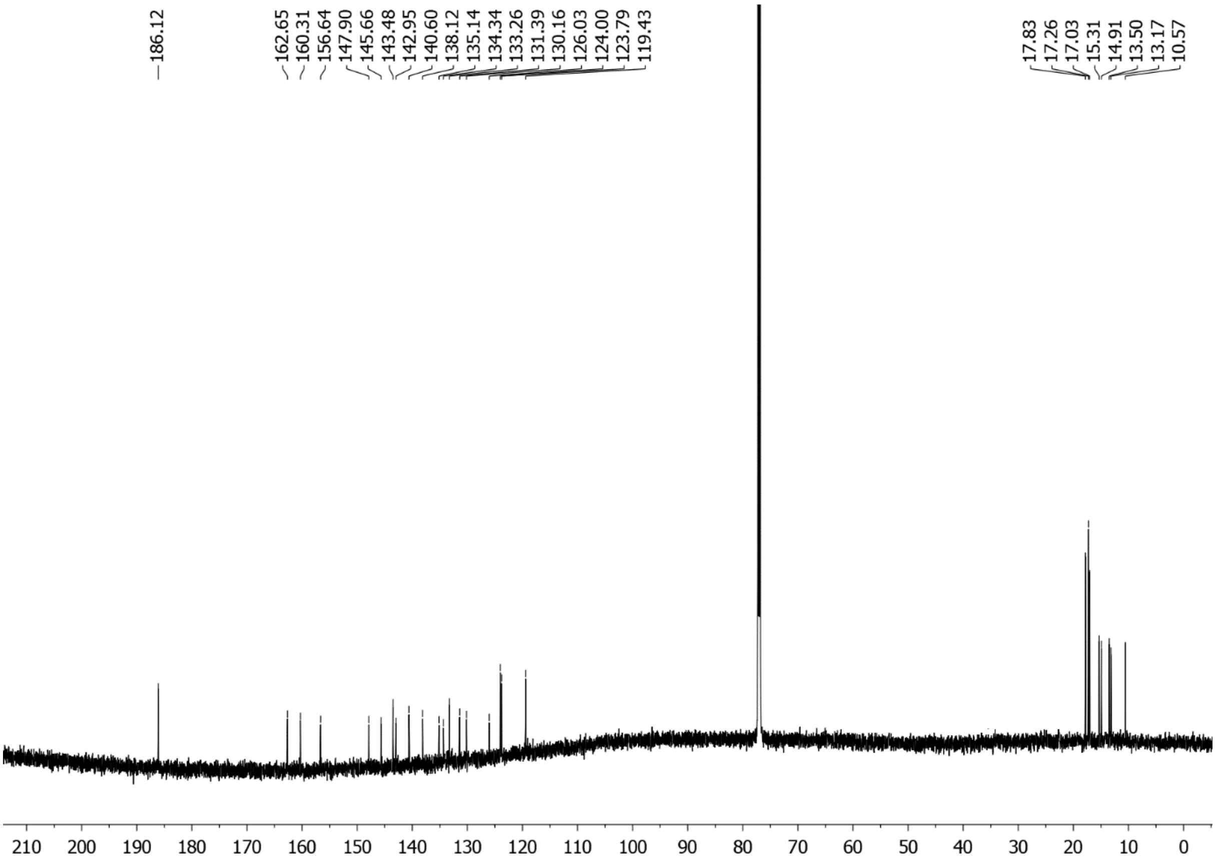

^{1}H NMR (700 MHz, $CDCl_3$) and ^{13}C NMR (176 MHz, $CDCl_3$) spectra of **8b**

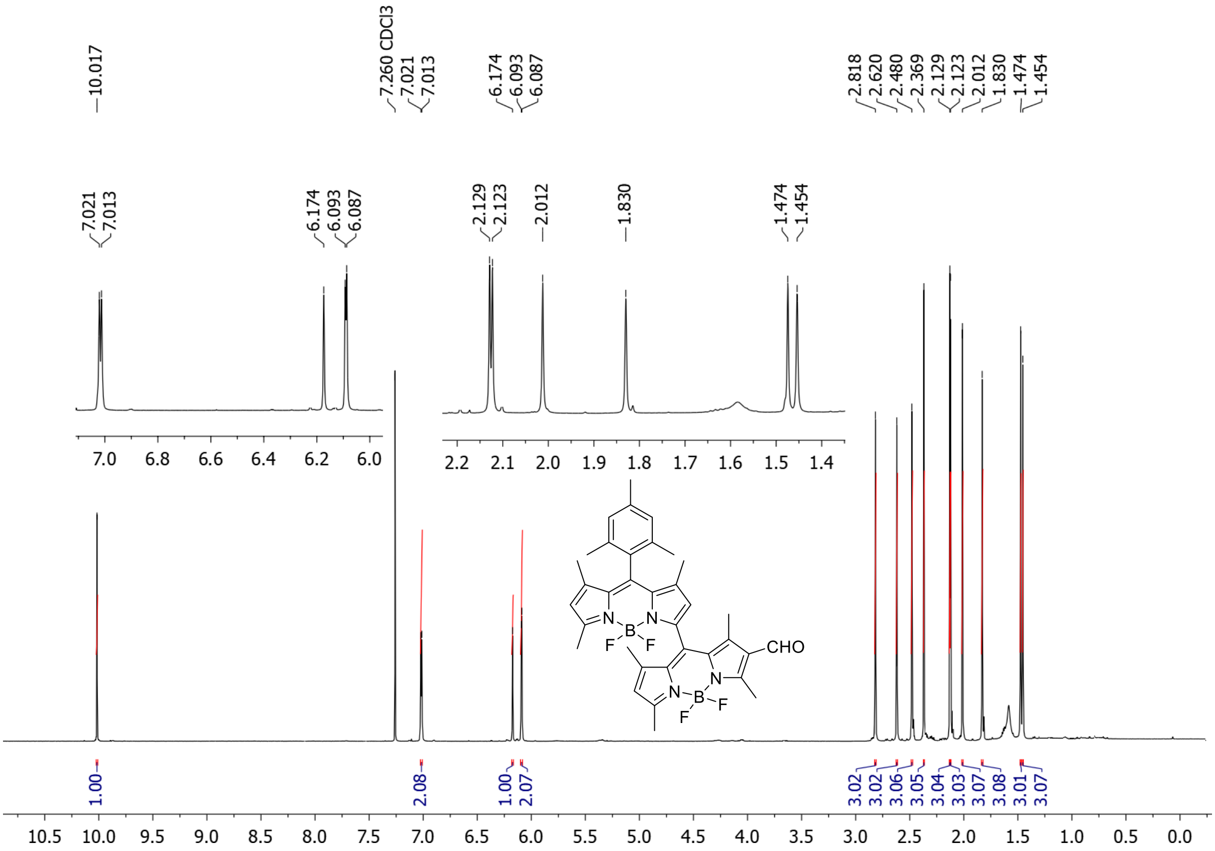

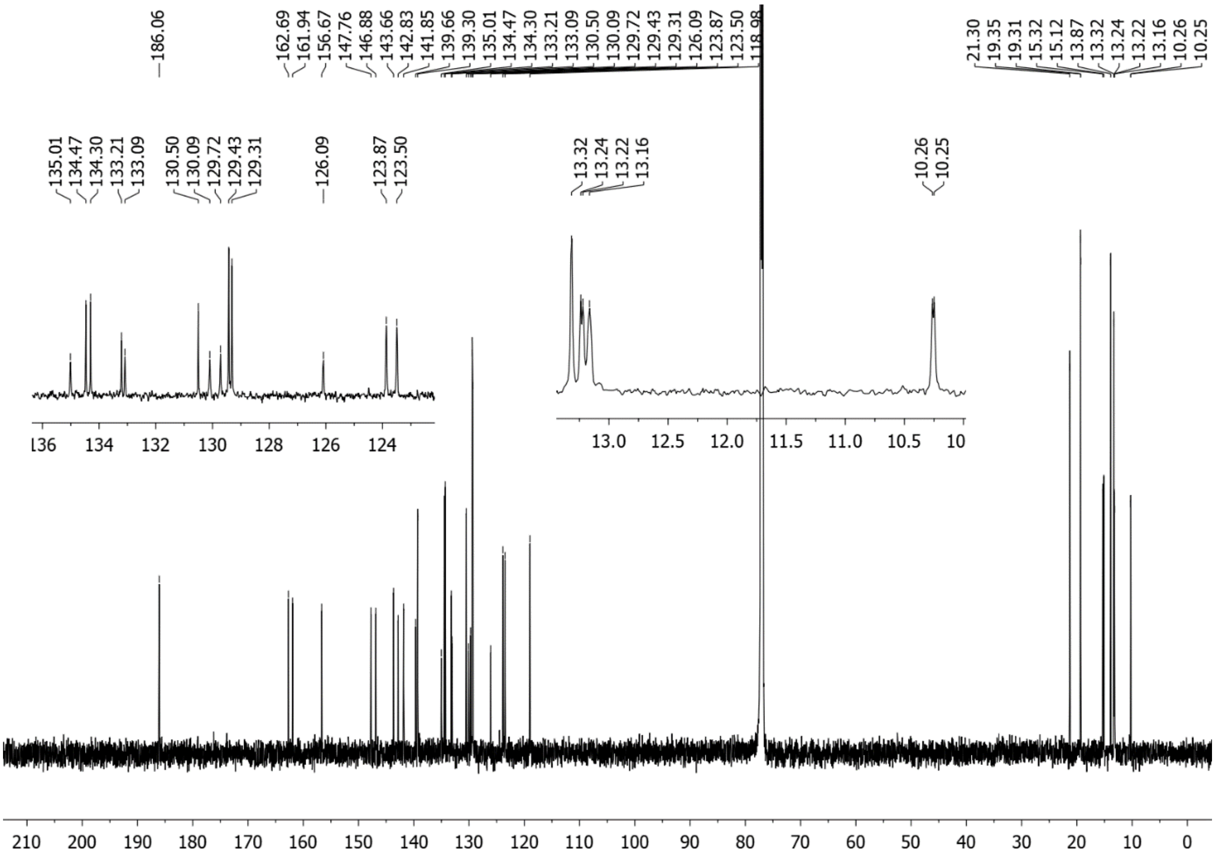

^{1}H NMR (700 MHz, $CDCl_3$) and ^{13}C NMR (176 MHz, $CDCl_3$) spectra of **9**

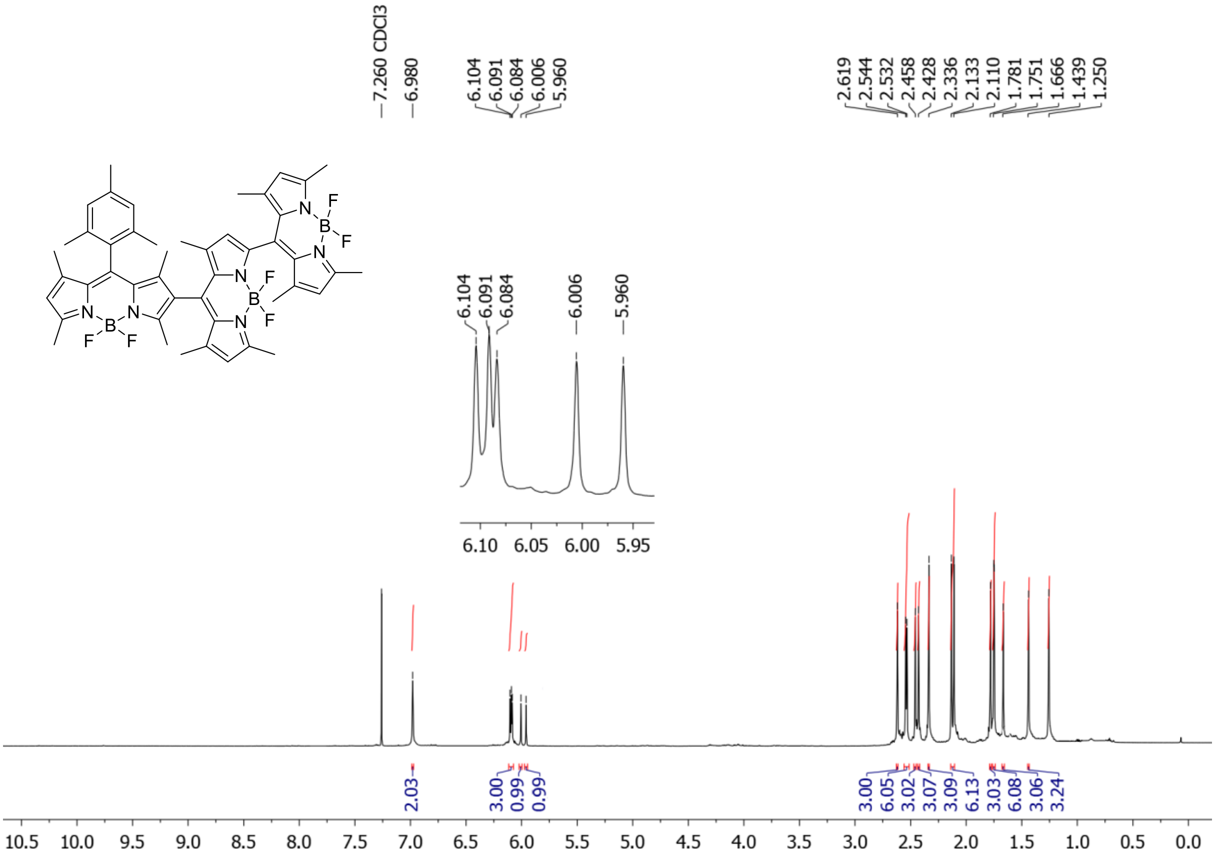

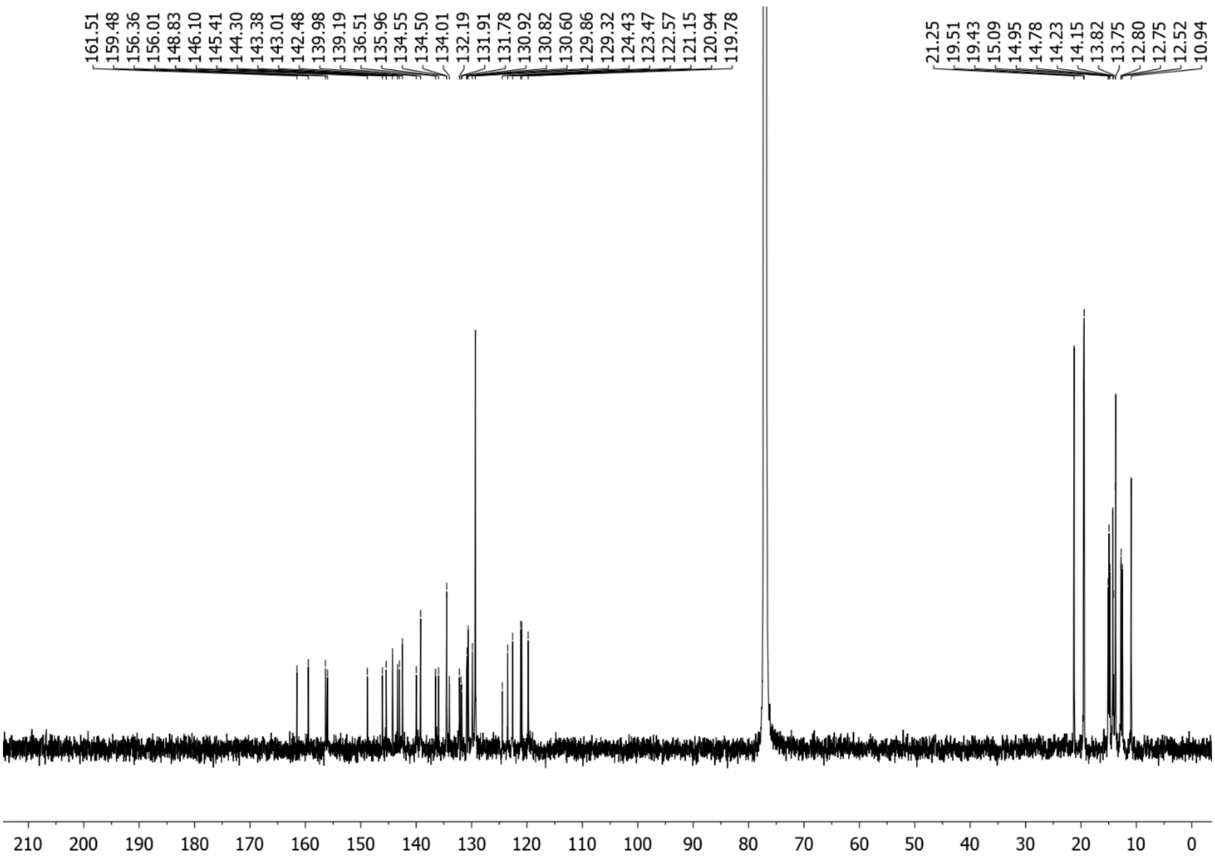

^{1}H NMR (700 MHz, $CDCl_3$) and ^{13}C NMR (176 MHz, $CDCl_3$) spectra of **10**

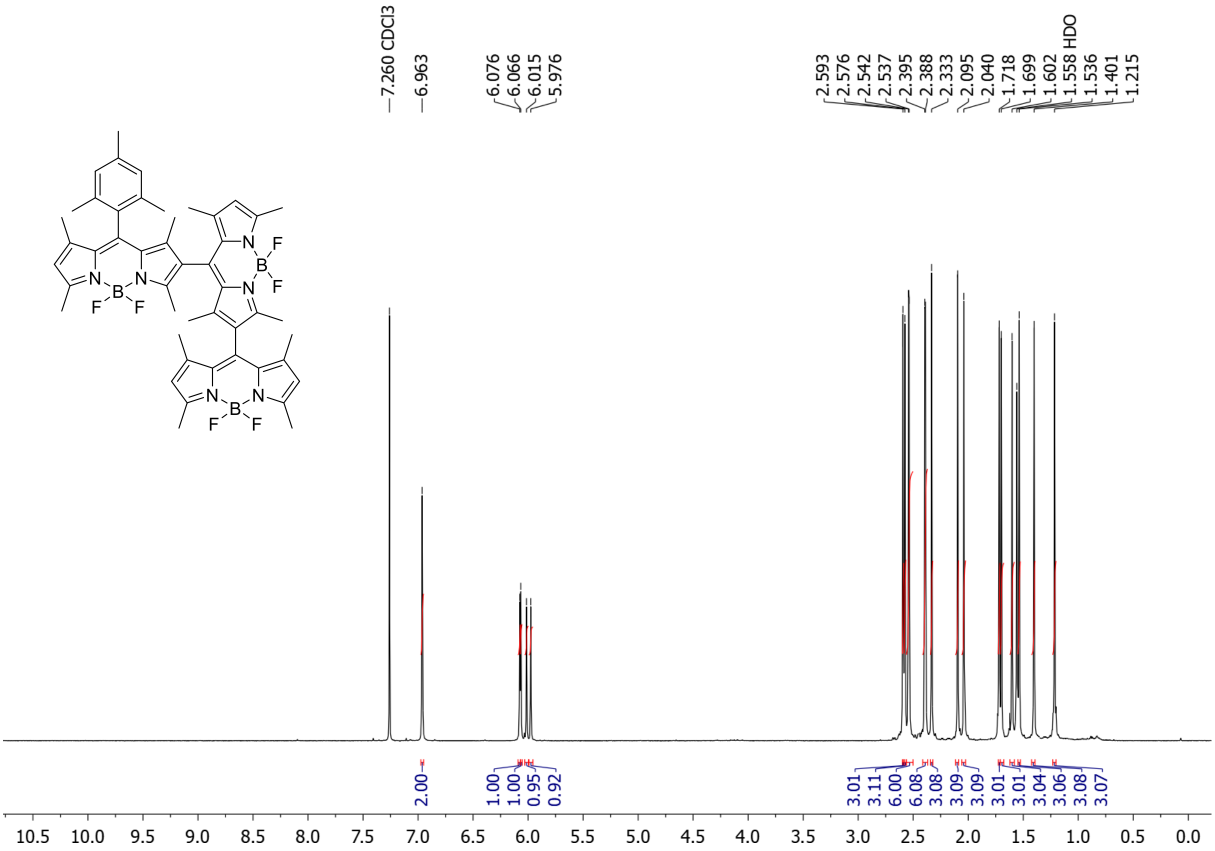

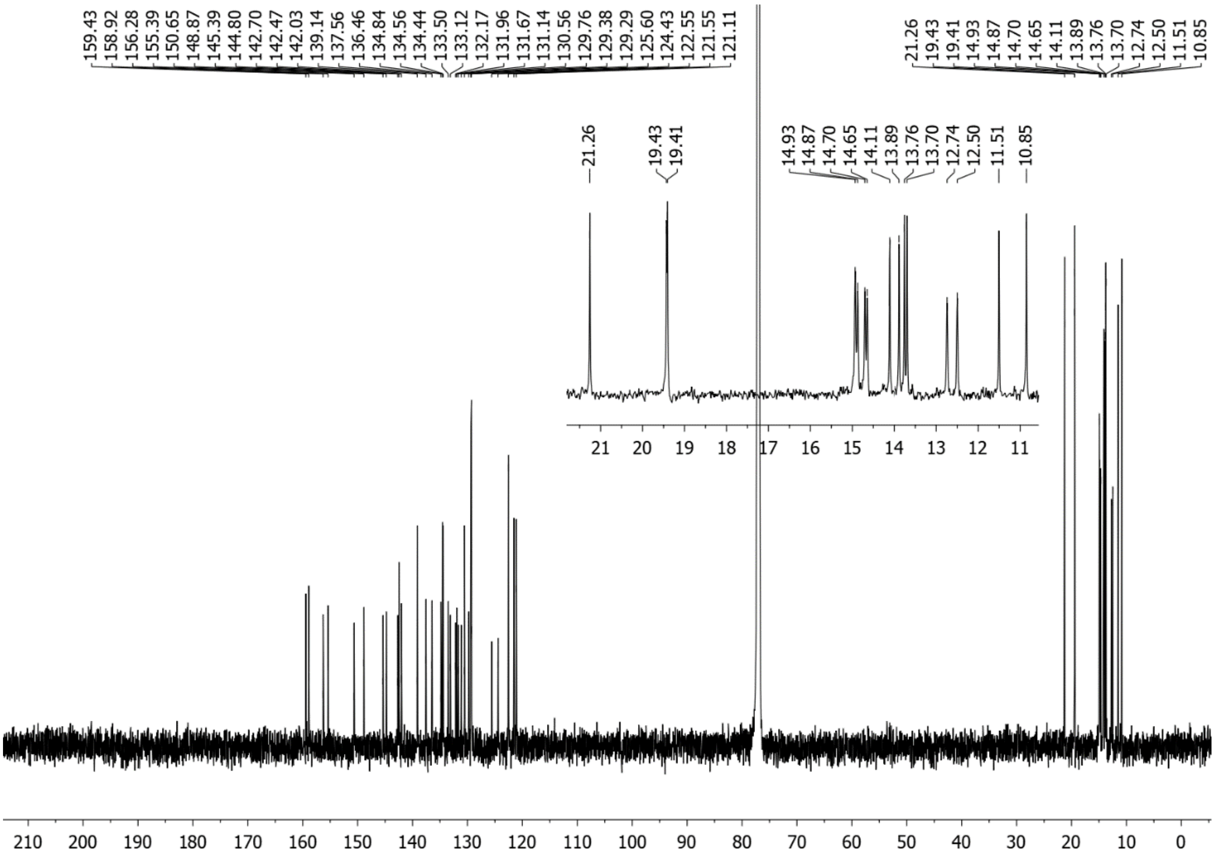

^{1}H NMR (700 MHz, $CDCl_3$) and ^{13}C NMR (176 MHz, $CDCl_3$) spectra of **11**

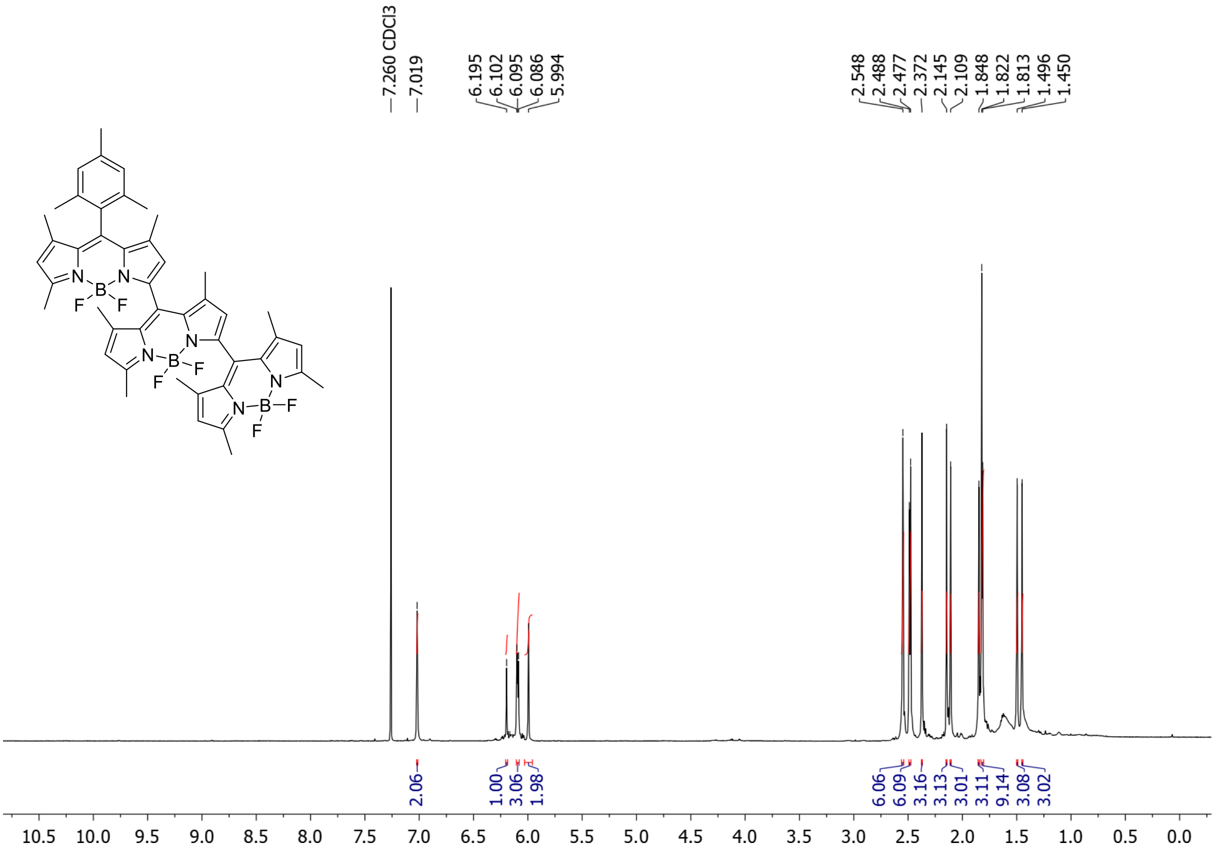

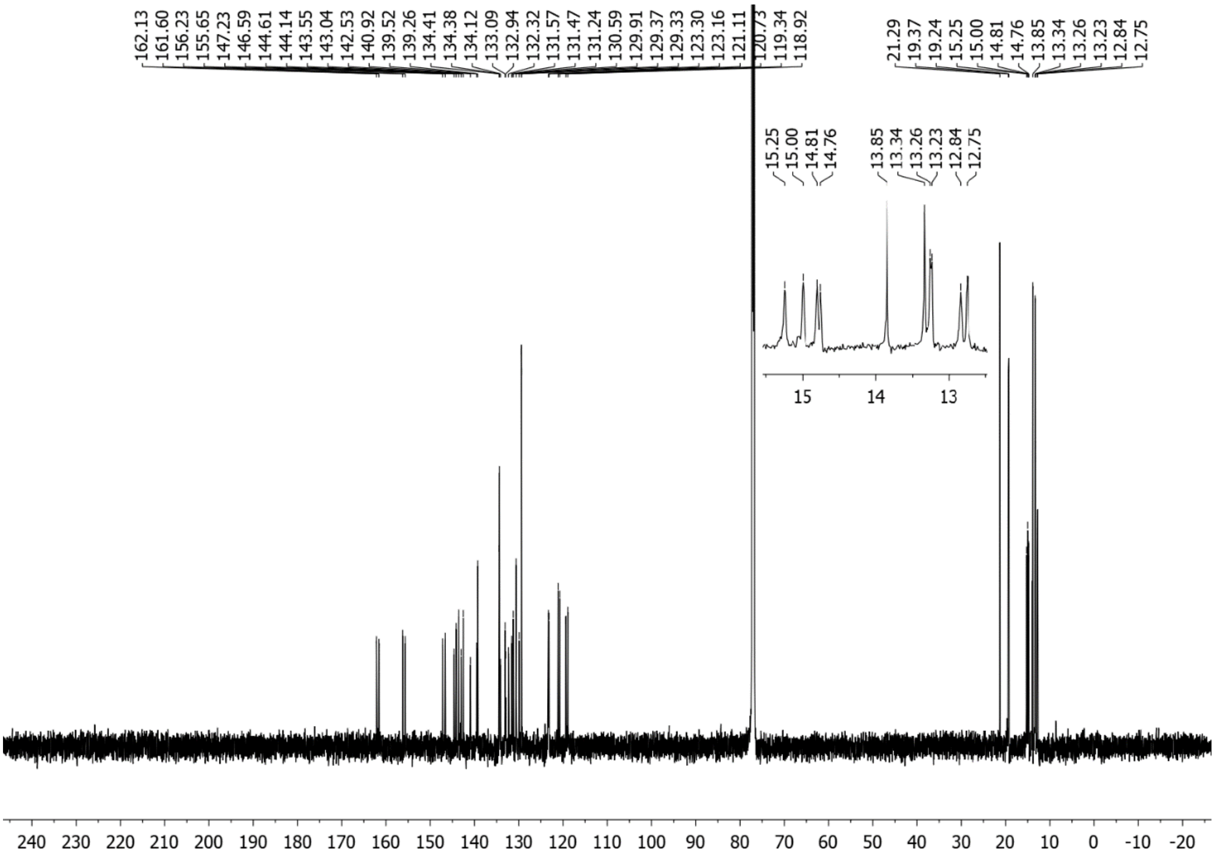

^{1}H NMR (700 MHz, $CDCl_3$) and ^{13}C NMR (176 MHz, $CDCl_3$) spectra of **12**

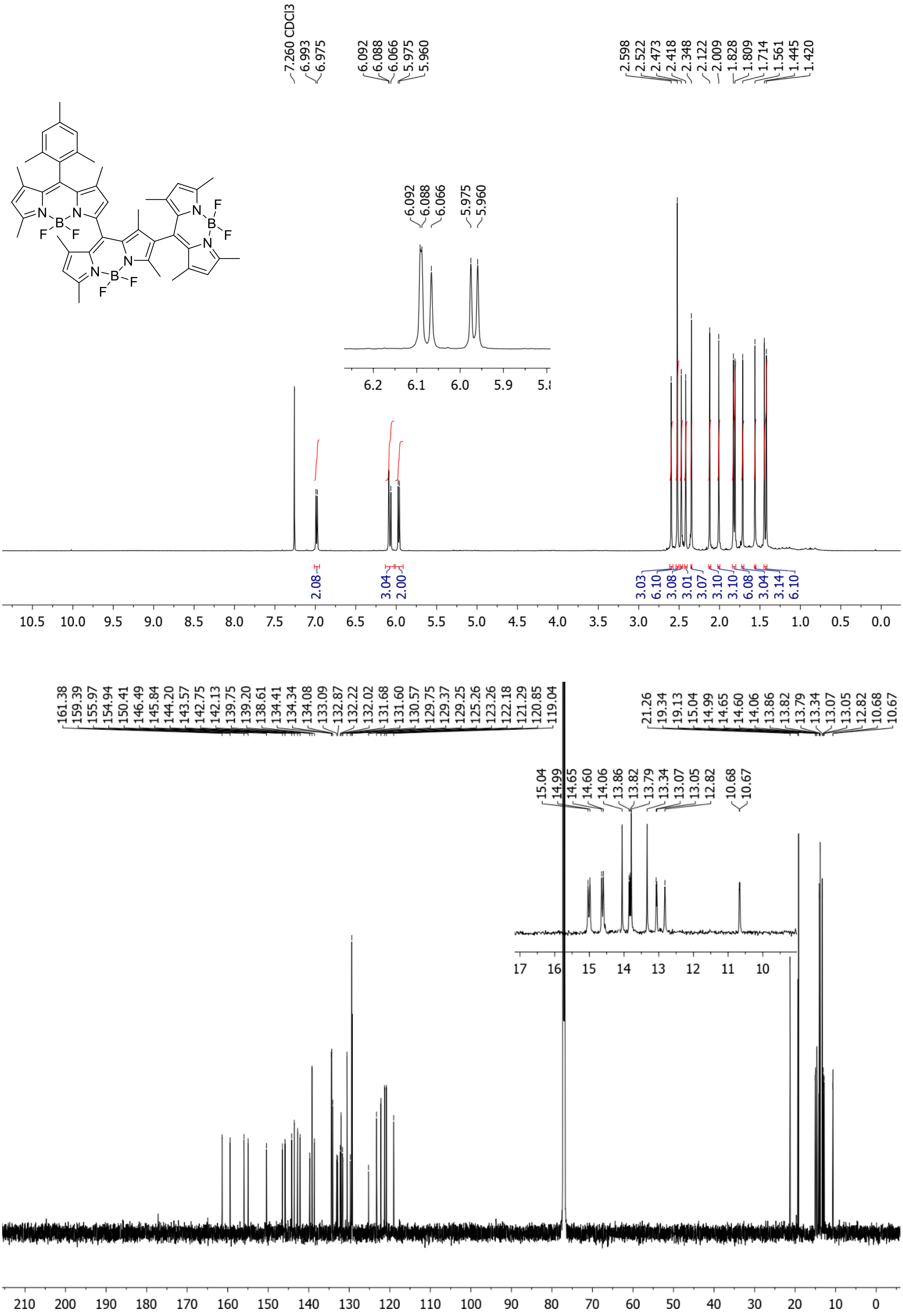

4. 1D-NOESY experiments

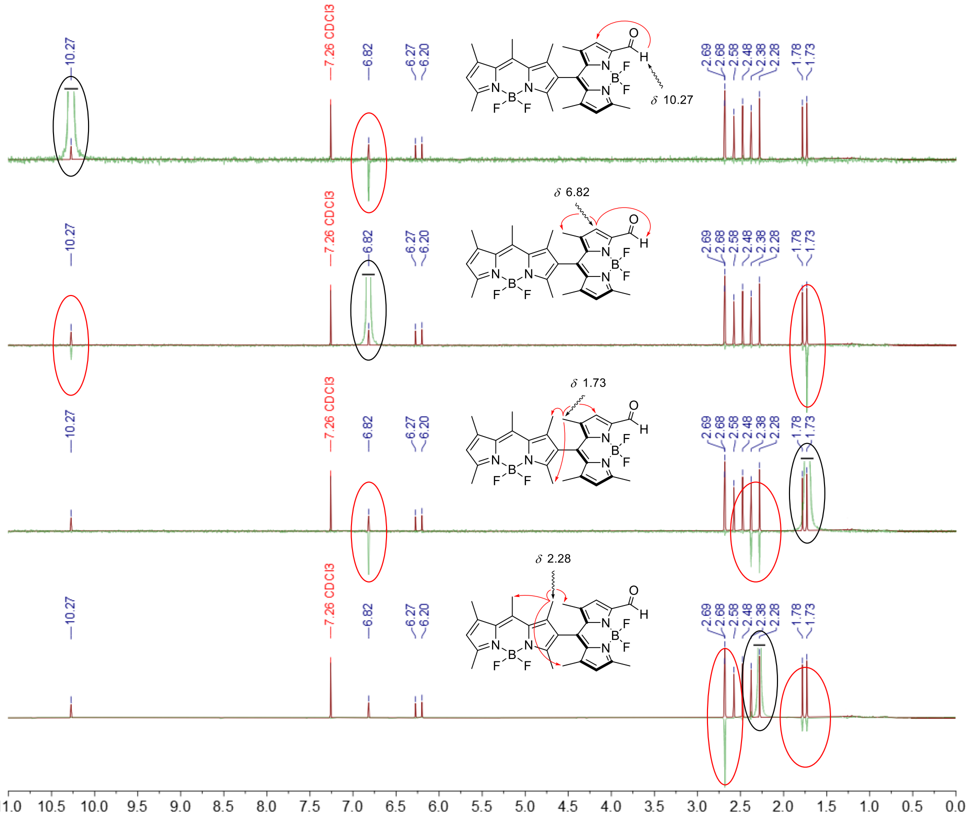

Figure S1. 1D-NOESY (green) referenced to ^{1}H-NMR (garnet) in $CDCl_3$ for **2a**: irradiated nucleus (black circle) and NOE signal (red circle). The structure of **2a** is depicted showing the effects observed in each experiment.

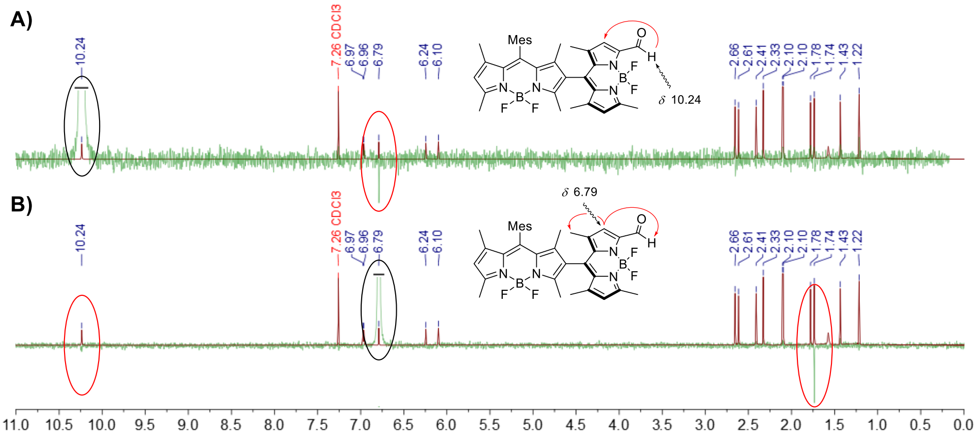

Figure S2. 1D-NOESY (green) referenced to ^{1}H-NMR (garnet) in $CDCl_3$ for **2b**: irradiated nucleus (black circle) and NOE signal (red circle). The structure of **2b** is depicted showing the effects observed in each experiment.

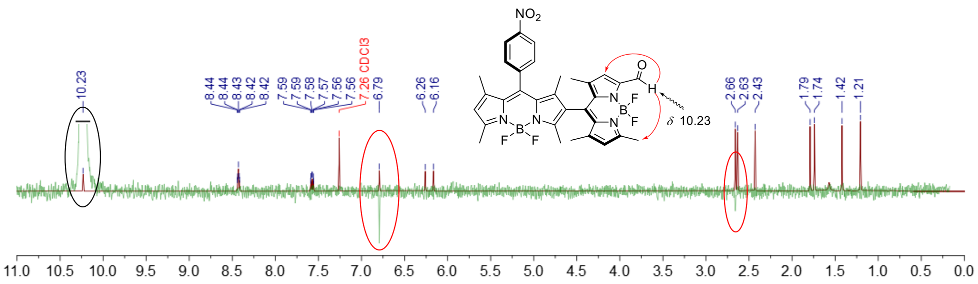

Figure S3. 1D-NOESY (green) referenced to ^{1}H-NMR (garnet) in $CDCl_3$ for **2d**: irradiated nucleus (black circle) and NOE signal (red circle). The structure of **2d** is depicted showing the effects observed in each experiment.

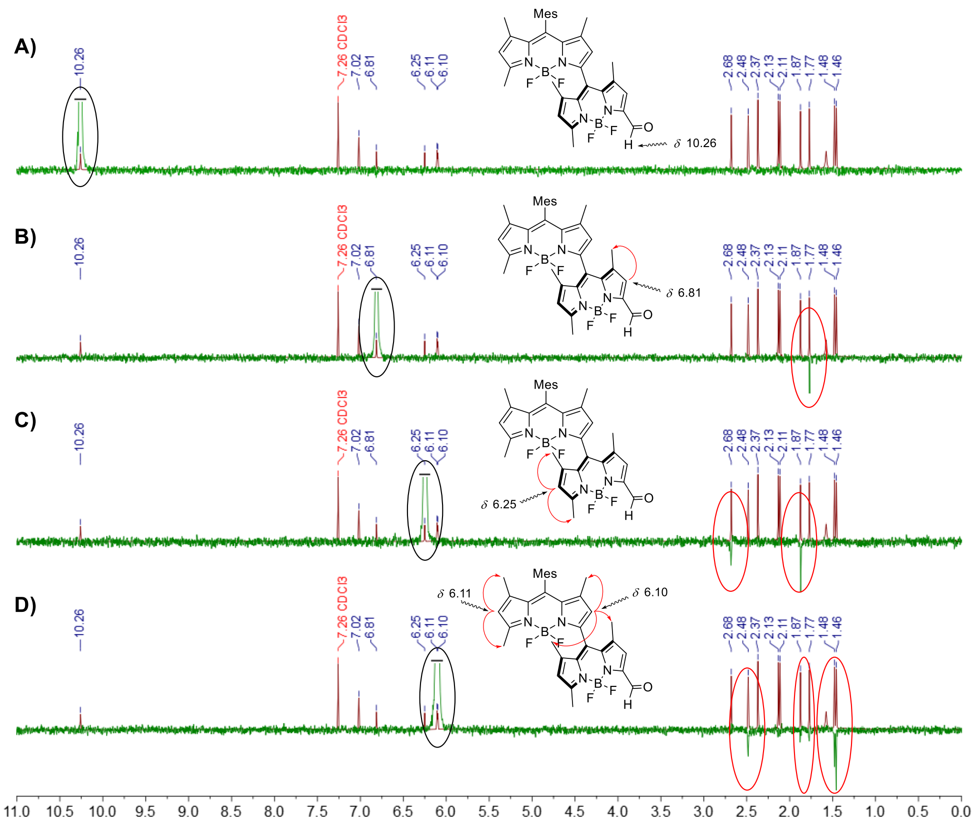

Figure S4. 1D-NOESY (green) referenced to ^{1}H-NMR (garnet) in $CDCl_3$ for **7b**: irradiated nucleus (black circle) and NOE signal (red circle). The structure of **7b** is depicted showing the effects observed in each experiment.

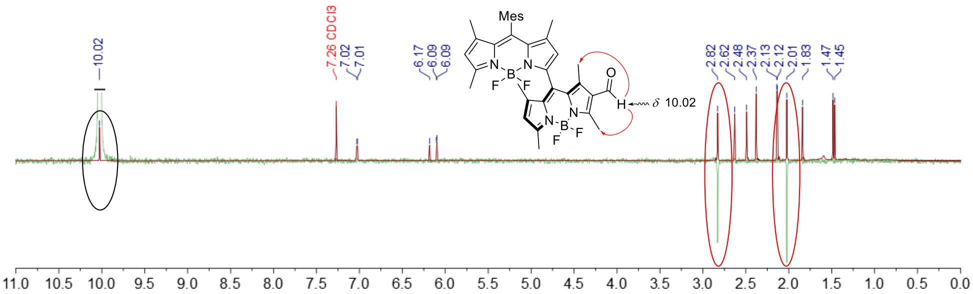

Figure S5. 1D-NOESY (green) referenced to ^{1}H-NMR (garnet) in $CDCl_3$ for **8b**: irradiated nucleus (black circle) and NOE signal (red circle). The structure of **8b** is depicted showing the effects observed in each experiment.

5. Photophysical and computational results

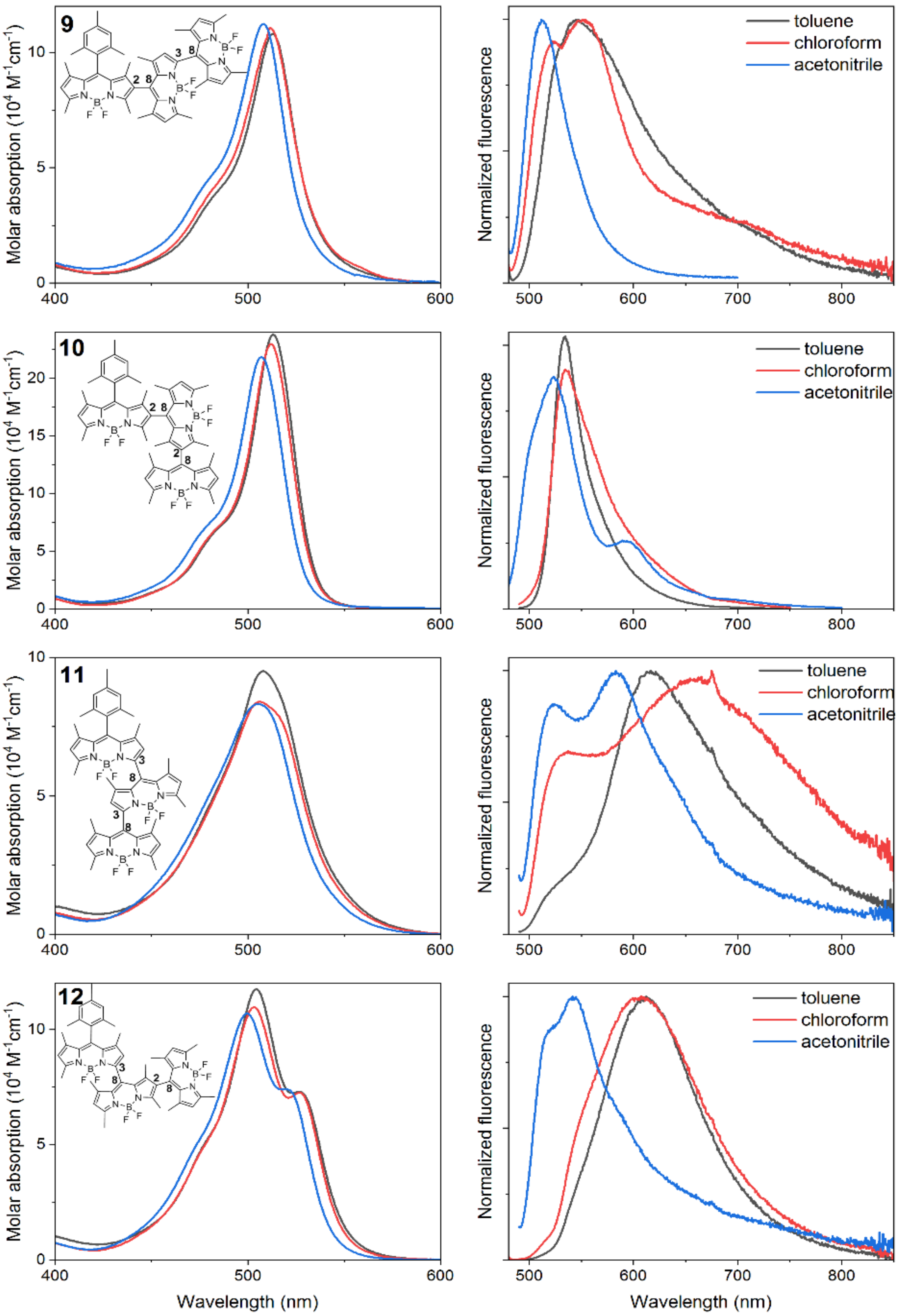

Figure S6. Absorption and normalized fluorescence spectra of the orthogonal trimers **9-12** in diluted solutions (2 mM).

**9**<br><br>2-8' → 88º<br>3-8' → 87º	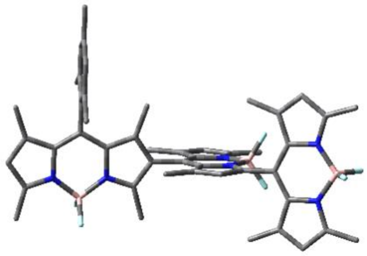	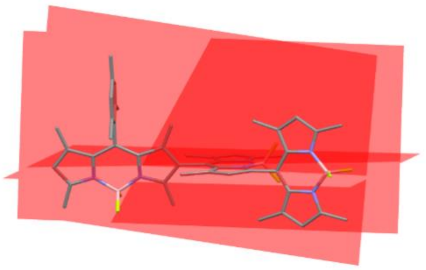
**10**<br><br>2-8' → 85º<br>2-8' → 86º	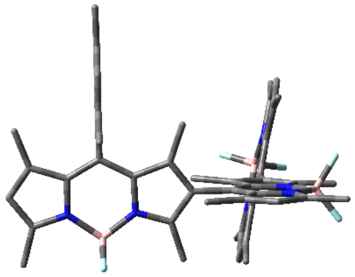	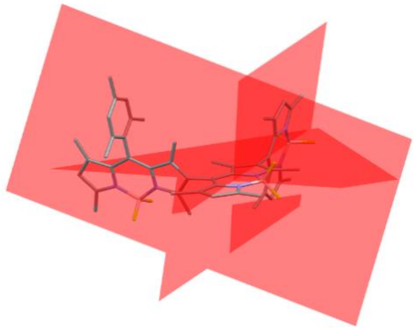
**11**<br><br>3-8' → 89º<br>3-8' → 88º	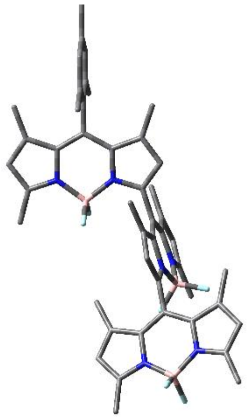	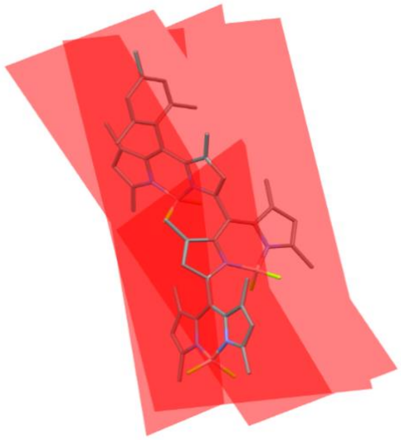
**12**<br><br>3-8' → 89º<br>2-8' → 89º	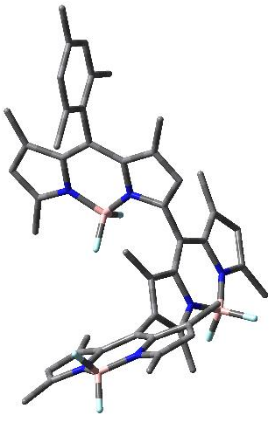	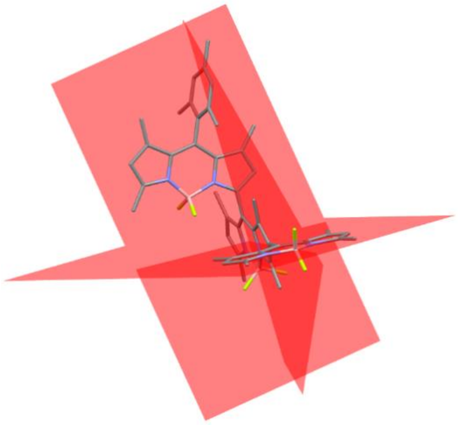

Figure S7. Theoretically optimized ground state geometry (wb97xd/6-311g*) of the trimers **9**-**12** in two different views to highlight the orthogonal arrangement of the chromophoric subunits. The corresponding dihedral angles between the chromophoric planes at the linkage positions are also given.

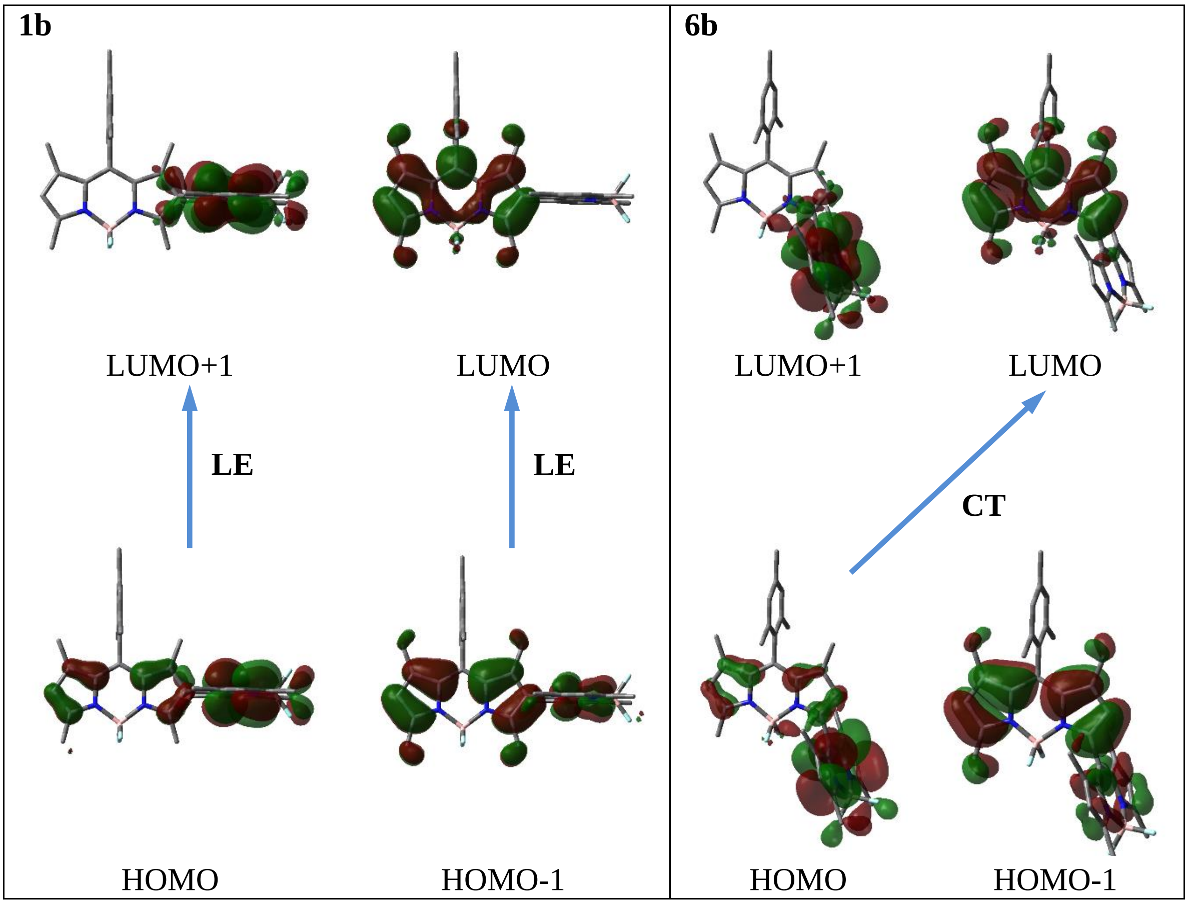

Figure S8. Contour maps of the molecular orbitals involved in the main electronic transitions predicted by TD CAM-B3LYP/6-311G* calculation for the orthogonal dimers **1b** and **6b**. The involved MOs in the transition to the lowest energy excited states, responsible of the recorded absorption spectra, are highlighted by arrows indicating the character of the transition according to the TD DFT method (local excitation of the BODIPY subunit, LE, or charge transfer character, CT).

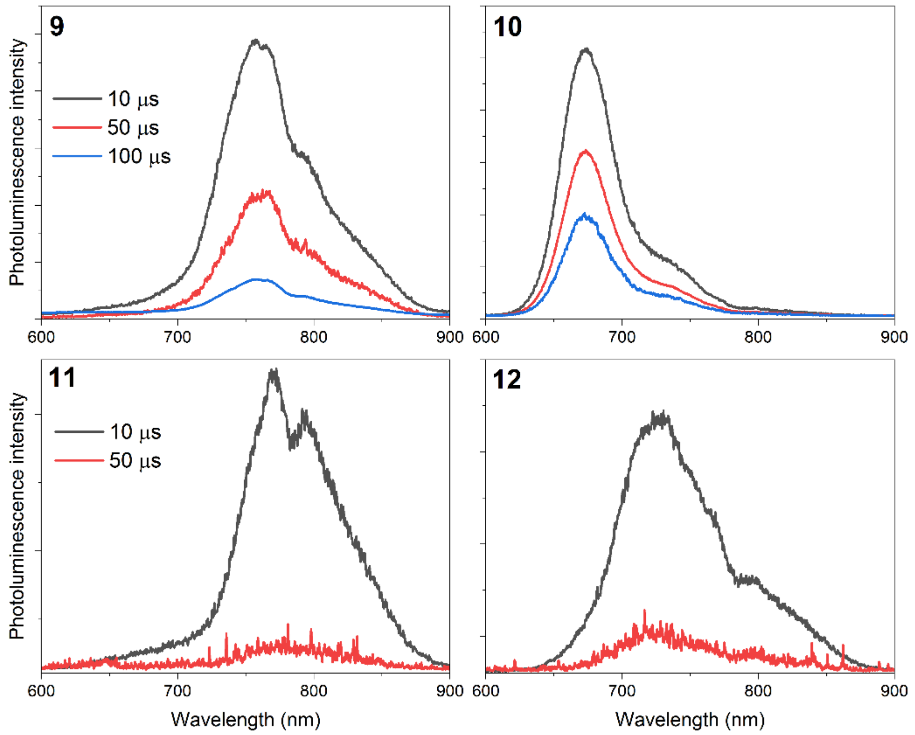

Figure S9. Room temperature phosphorescence emission of trimers **9-12** in chloroform. Optically matched solutions were used.

Table S1. Photophysical properties of the all-BODIPY based **9-12** orthogonal trimers in diluted solutions

		λ_{ab} (nm)	$\varepsilon_{max}\cdot10^{-4}$ $M^{-1}\cdot cm^{-1}$	λ_{fl} (nm)	ϕ	τ (ns)	ϕ^{Δ}
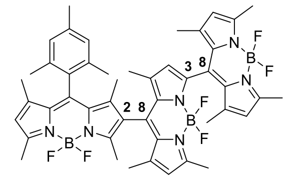	**9**						
	Toluene	513.0	10.8	544.5	0.091	0.59 (19%) - 3.20 (56%) - 4.52 (26%)	0.49
	Chloroform	511.5	11.1	553.0 (522.0)	0.042	0.18 (23%) - 2.24 (33%) - 4.02 (44%)	0.38
	Acetonitrile	508.0	11.2	511.5	0.007	0.28 (18%) - 1.86 (21%) - 4.68 (62%)	0.04
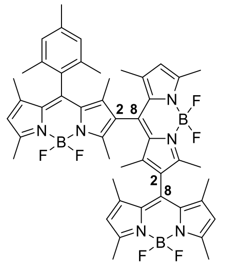	**10**						
	Toluene	513.0	23.5	534.5	0.232	0.02 (72%) - 6.43 (28%)	0.78
	Chloroform	512.0	22.7	534.0	0.038	0.02 (90%) - 3.86 (10%)	0.91
	Acetonitrile	507.0	21.6	522.0	0.003	0.02 (86%) - 1.06 (5%) - 4.76 (9%)	0.07
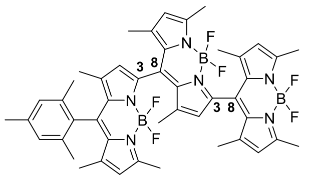	**11**						
	Toluene	507.5	9.5	616.5	0.019	0.25 (16%) - 1.69 (30%) - 3.89 (54%)	0.29
	Chloroform	506.0	8.4	660.0 (536.5)	0.010	0.05 (74%) - 1.45 (14%) - 4.55 (11%)	0.26
	Acetonitrile	505.0	8.3	583.0 (523.0)	0.002	0.10 (75%) - 2.61 (9%) - 9.45 (15%)	0.03
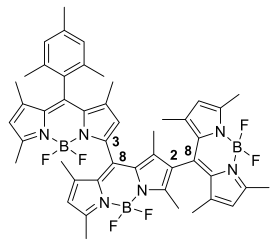	**12**						
	Toluene	504.5	11.7	612.0	0.179	5.42	0.72
	Chloroform	503.0	11.0	607.0	0.073	0.50 (16%) - 3.65 (84%)	0.58
	Acetonitrile	499.5	10.7	541.5	0.006	0.06 (81%) - 1.47 (6%) - 5.18 (13%)	0.05

Absorption (λ_{ab}) and fluorescence (λ_{fl}) wavelength, molar absorption coefficient (ε_{max}), fluorescence quantum yield (ϕ) and lifetime (τ), and singlet oxygen generation (ϕ^{Δ}).

Table S2. Simulation of the absorption properties (TD CAM-B3LYP/6-311G*) of orthogonal dimers **1b** and **6b**. The corresponding contour maps of the involved frontier MOs are depicted in Figure S8.

	State (MOs)	ΔE (eV)	*f*	Type
**1b**	S_1 (H-1 → L)	2.93	0.68	LE
	S_2 (H → L+1)	2.97	0.49	LE
	S_3 (H → L)	3.31	0.01	CT
**6b**	S_1 (H → L 78%; H-1 → L 22%)	2.83	0.10	CT
	S_2 (H → L+1)	2.91	0.48	LE
	S_3 (H-1 → L)	3.00	0.43	LE

ΔE = energy gap; *f* = oscillator strength; H = HOMO; L = LUMO

Table S3. Photophysical properties of the 8-mesitylBODIPY based orthogonal dimers **1b** and **6b** in diluted solutions

		λ_{ab} (nm)	$\varepsilon_{max}\cdot10^{-4}$ $M^{-1}\cdot cm^{-1}$	λ_{fl} (nm)	ϕ	τ (ns)	ϕ^{Δ}
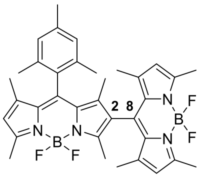	**1b**						
	Toluene	511.0	17.3	525.0	0.46	1.56 (5%) - 5.87 (95%)	0.41
	Chloroform	511.0	16.5	525.0	0.19	0.02 (78%) - 5.02 (22%)	0.94
	Acetonitrile	505.0	15.4	512.5	0.03	0.63 (10%) - 4.67 (90%)	0.65
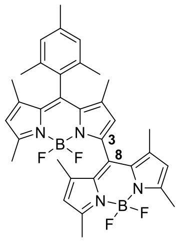	**6b**						
	Toluene	509.0	7.3	529.0 (596.0)	0.021	1.27 (17%) - 4.49 (83%)	0.84
	Chloroform	507.0	6.7	522.5	0.011	1.59 (25%) - 4.84 (75%)	0.64
	Acetonitrile	506.0	6.5	518.0	0.004	-	0.07

6. Biological studies

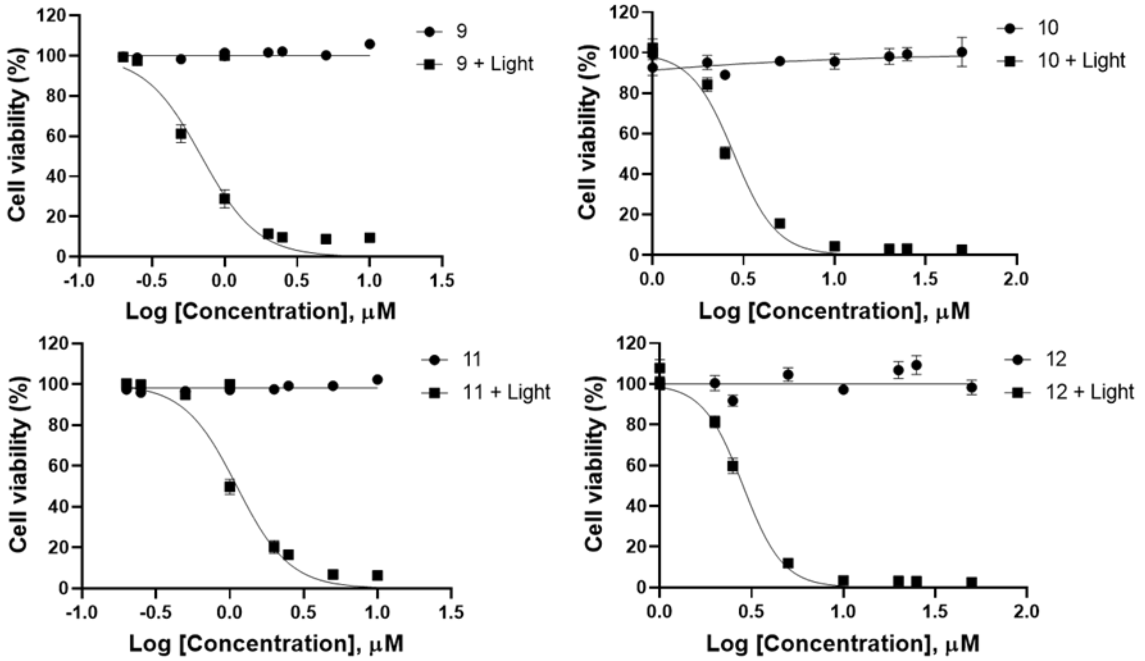

Figure S10. Sigmoidal fitting of concentration-cell viability curve for all-orthogonal BODIPY trimers **9-12** in the absence (circle) and presence (square) of visible-light (λ > 475 nm) for 0.5 h. Cell viability was determined 24 h after the irradiation step and in dark with WST-1 method. Values are expressed as mean ± SEM (n=4).

Table S4. IC_{50} values for all-orthogonal BODIPY trimers **9-12** against SK-Mel-103 cells in the presence of light-irradiation for 0.5 h. Light conditions were carried out using a 36 W LED device at a distance of 10 cm with a light filter to allow passage of only suitable wavelengths (Newport, λ > 475 nm).

**BODIPY Trimers**	IC_{50}^{Light} **(μM)**
**9**	0.69
**10**	2.80
**11**	1.12
**12**	2.84

Table S5. Pearson's correlation coefficient (Rr) values of subcellular co-localization studies of trimers **10** and **12** in SK-Mel-103 stained with LysoTracker® Deep Red, MitoTracker® Deep Red FM and ER-Tracker Blue-White DPX by CLMS. Values are expressed as mean ± SEM of at least three independents experiments. (The parameter of CLMS: trimer **10** and **12** were excited at 488 nm and the emission was collected at 513-567 nm; LysoTracker® Deep Red and MitoTracker® Deep Red FM were excited at 638 nm and the emission was collected at 640-780 nm, and ER-Tracker® Blue-White DPX was excited at 405 nm and the emission was collected at 410-480 nm).

**BODIPY Trimers**	**LysoTracker® Deep Red**	**MitoTracker® Deep Red FM**	**ER-Tracker Blue-White DPX**
**10**	0.71 ± 0.06	0.18 ± 0.05	0.48 ± 0.08
**12**	0.70 ± 0.06	0.28 ± 0.04	0.51 ± 0.04

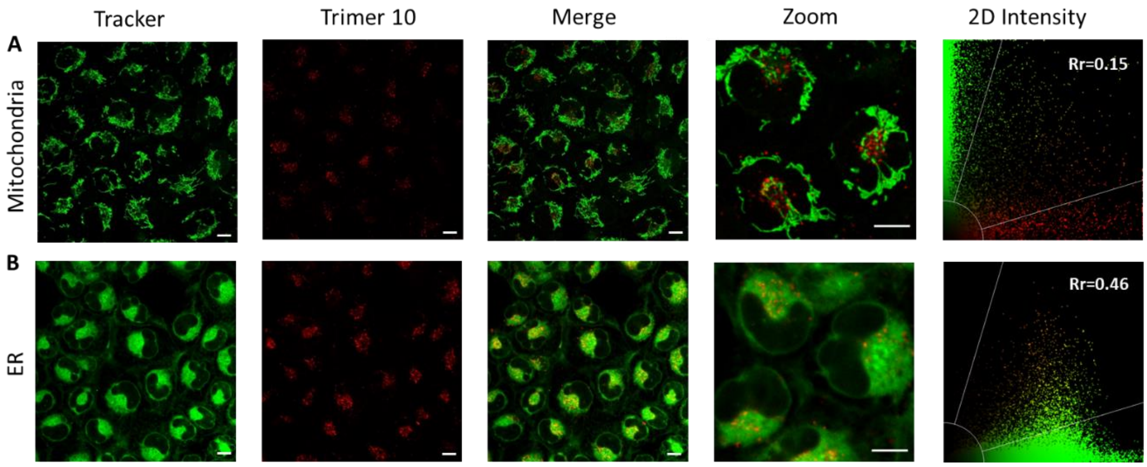

Figure S11. Confocal fluorescence images of subcellular co-localization studies of trimer **10** (2.5 μM) in SK-Mel-103 stained with MitoTracker® Deep Red FM (A) and ER-Tracker® Blue-White DPX (B). Areas of co-localization appear in yellow/orange in Merged. The Pearson's co-localization coefficient (Rr) for trimer **10** and trackers channel in the zoomed image is provided in the column of 2D intensity scatterplot diagram which represents correlation between pixel intensities in both channels. Scale bar: 10 µm. (The parameter of CLMS: trimer **10** was excited at 488 nm and the emission was collected at 513-567 nm; MitoTracker® Deep Red FM was excited at 638 nm and the emission was collected at 640-780 nm, and ER-Tracker Blue-White DPX was excited at 405 nm and the emission was collected at 410-480 nm).

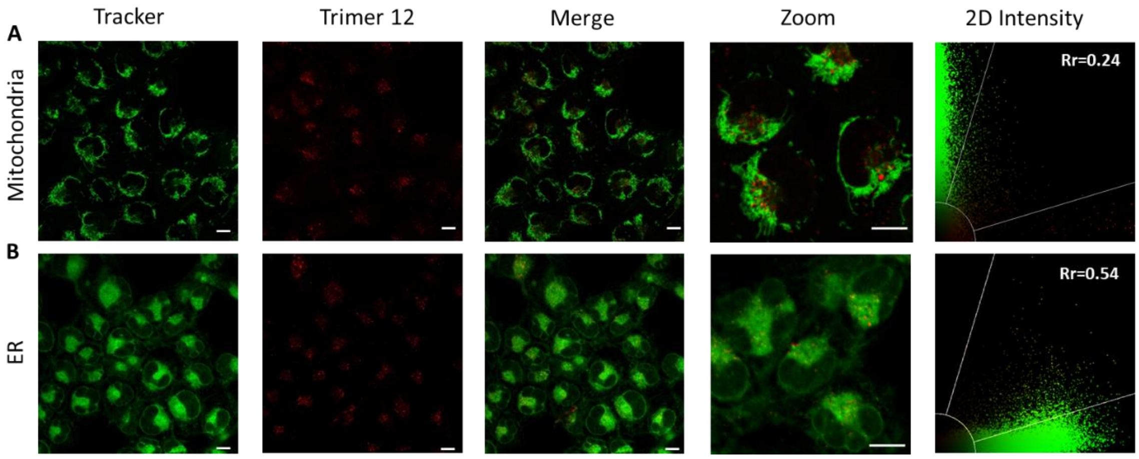

Figure S12. Confocal fluorescence images of subcellular co-localization studies of trimer **12** (5.0 μM) in SK-Mel-103 stained with MitoTracker® Deep Red FM (A) and ER-Tracker® Blue-White DPX (B). Areas of co-localization appear in yellow/orange in Merged. The Pearson's co-localization coefficient (Rr) for trimer **12** and trackers channel in the zoomed image is provided in the column of 2D intensity scatterplot diagram which represents correlation between pixel intensities in both channels. Scale bar: 10 µm. (The parameter of CLMS: trimer **12** was excited at 488 nm and the emission was collected at 513-567 nm; MitoTracker® Deep Red FM was excited at 638 nm and the emission was collected at 640-780 nm, and ER-Tracker® Blue-White DPX was excited at 405 nm and the emission was collected at 410-480 nm).

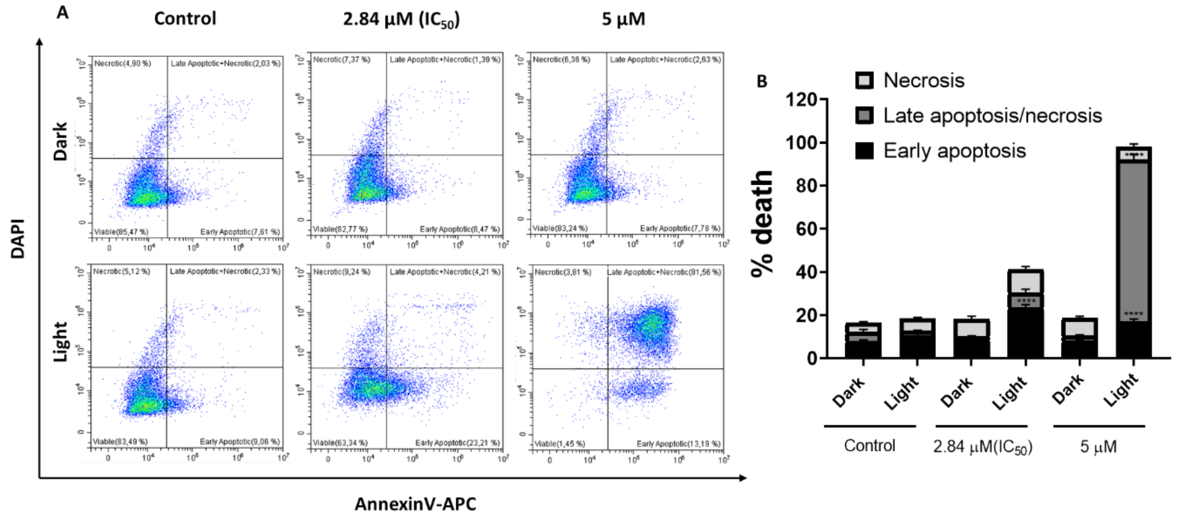

Figure S13. Cell apoptosis/necrosis assay by flow cytometry in SK-Mel-103 cells after PDT treatment with trimer **12** at various doses. (A) Dot plot diagrams for apoptotic cell population with Annexin V-APC and DAPI staining. (B) Quantification of early apoptotic, late apoptotic/necrotic, and necrotic cells. Percentages are expressed as mean ± SEM of at least three independents experiments, and statistical significance was assessed by two-way ANOVA and Tukey's post-test relative to the same treatment without irradiation. **** <0.0001 indicate statistically significant changes.

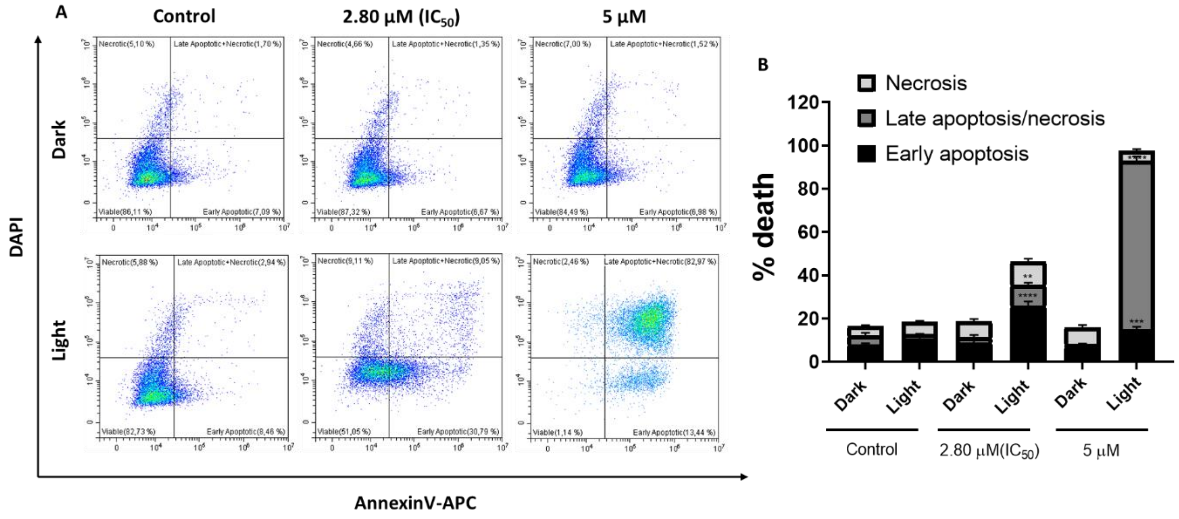

Figure S14. Cell apoptosis/necrosis assay by flow cytometry in SK-Mel-103 cells after PDT treatment with trimer **10** at various doses. (A) Dot plot diagrams for apoptotic cell population with Annexin V-APC and DAPI staining. (B) Quantification of early apoptotic, late apoptotic/necrotic, and necrotic cells. Percentages are expressed as mean ± SEM of at least three independents experiments, and statistical significance was assessed by two-way ANOVA and Tukey's post-test relative to the same treatment without irradiation. ** $p<0.01$, *** $p<0.001$ and **** $p<0.0001$ indicate statistically significant changes.

7. References

(1) López Arbeloa, F.; Bañuelos, J.; Martínez, V.; Arbeloa, T.; López Arbeloa, I. *Int. Rev. Phys. Chem*. **2005**, *24*, 339-374.

(2) Wu, W.; Cui, X.; Zhao, *J. Chem. Commun*. **2013**, *49*, 9009-9011.

(3) Epelde-Elezcano, N.; Palao, E.; Manzano, H.; Prieto-Castañeda, A.; Agarrabeitia, A. R.; Tabero, A.; Villanueva, A.; De la Moya, S.; López-Arbelos, I.; Martínez-Martínez, V.; Ortiz, M. J., *Chem. Eur. J.* **2017**, *23*, 4837-4848.

(4) Durán-Sampedro, G.; Epelde-Elezcano, N.; Martínez-Martínez, V.; Esnal, I.; Bañuelos, J.; García-Moreno, I.; Agarrabeitia, A. R.; De la Moya, S.; Tabero, A.; Lazaro-Carrillo, A.; Villanueva, A.; Ortiz, M. J.; López-Arbeloa, I., *Dyes Pigm.* **2017**, *142*, 77-87.

(5) Jiao, L.; Yu, C.; Wang, Z.; Wu, M.; Hao, E., *J. Org. Chem.* **2009**, *74*, 7525-7528.

(6) Ramos-Torres, A.; Avellanal-Zaballa, E.; Prieto-Castañeda, A.; García-Garrido, F.; Bañuelos, J.; Agarrabeitia, A. R.; Ortiz, M. J., *Org. Lett.* **2019**, *21*, 4563-4566
